# Supplementary material for: Approaches to improving patient safety in integrated care: a scoping review
Source: BMJ Open. 2023 Apr 4;13(4):e067441. doi: 10.1136/bmjopen-2022-067441 (PMC10083780; doi:10.1136/bmjopen-2022-067441)
Supplement: Supplementary data [file bmjopen-2022-067441supp002.pdf]

Additional File 1 – search strategy

Search terms (including MeSH) used were:

elderly (elder*) OR older OR older adults OR geriatric OR aged

vulnerable adults

mental health OR disorder

learning disabilities (*disability)

(-term OR long term) conditions

complex care

chronic conditions

multi-morbidity

AND

integrated care

care pathways

multidisciplinary OR multi-disciplinary

multiprofessional OR multi-professional

interprofessional OR inter-professional

transitional (W#) care (transitio*)

care (W#) coordination

AND

patient safety

preventable (w#) harm

risk reduction

adverse events
