# Supplementary material for: Approaches to improving patient safety in integrated care: a scoping review
Source: BMJ Open. 2023 Apr 4;13(4):e067441. doi: 10.1136/bmjopen-2022-067441 (PMC10083780; doi:10.1136/bmjopen-2022-067441)
Supplement: Supplementary data [file bmjopen-2022-067441supp001.pdf]

Supplementary table: Characteristics and description of included studies

Author and year	Country	Aim	Population	Study design	Integrated care approach				Approaches to measuring safety
					Type of approach	Description of approach	Setting	Level	
**Akbarov 2015 (33)**	England	Investigate the feasibility of wide ranging medication safety surveillance using a linked database	Adults with prescribing hazards	Cross-sectional study	Integrated digital record	Linked records of patients served by one hospital system and primary care centres - prevalence of 22 medication safety indicators, investigation of associations with patient and practice characteristics, and variation between primary care practices	Hospitals and primary care centres	Macro	Outcome measure: Proportion of patients with at least one medication safety hazard
**Amacher 2016 (34)**	Switzerland	Explore the perceived benefits and barriers of an evidence-based, home-based pilot falls prevention programme	Elderly	Mixed methods	MDT approach to care delivery in community (home) or primary care setting	Multi-factorial falls prevention programme (FPP)	Community (home)	Micro	Qualitative outcome: Usefulness of MDT home-based fall prevention programme
**Bell 2017 (35)**	Norway	Investigate nurses' and pharmacists' perceived learning experience after participating in an Integrated Medicines Management Review in primary health care	Elderly	Qualitative study	MDT approach to care delivery in community (home) or primary care setting	Integrated Medicines Management model – centred on inter-professional medication reviews	Community (home based services and nursing homes)	Macro	Qualitative outcome: Extent of professional learning experiences to support medicines management

**Brito 2017 (36)**	Portugal	Managing medication, in a patient-centred process and multidisciplinary context, by gathering information about the drug therapy of patients before admission, during hospitalization and after hospital discharge	Elderly	Observation study	Care pathway	Pharmacist working within domiciliary hospitalisation unit (DHU) alongside a multi-disciplinary team – medication reconciliation and review	Community (home) – domiciliary hospitalisatio n	Micro	Qualitative outcome: Perceived quality of DHU delivery and its impact on drug safety
**Di Monaco 2008 (37)**	Italy	Assessment of the effectiveness of a follow up home visit by an occupational therapist after a fall. MDT prevention intervention to reduce falls risk after hip fracture in elderly women	60 years or older	Quasi-RCT	Care pathway	MDT approach to prevent falls; exercise, advice and training on use of assistive devices, and activities of daily living	Hospital and community (home)	Micro	Outcome measure: Occurrence of falls in six months post-discharge from hospital
**Di Pollina 2017 (38)**	Switzerland	Testing the efficacy of providing integrated care at home to reduce unnecessary hospitalisations, emergency room visits, institutionalisation, and mortality in community	Frail and dependent people over 60 years	Prospective RCT	MDT approach to care delivery in community (home) or primary care setting	Integrated in-home geriatric assessments, long-term coordinated follow-up, and availability of a round-the-clock geriatric call service	Community (home)	Micro	Outcome measure: Falls over 3 years as a secondary outcome (stated as reason for hospitalisation)

		dwelling frail older adults							
**Eckstrom 2016 (27)**	US	Establishing changes in team behaviours after the intervention communicate and enhance adherence to Geriatrics Society Falls Prevention Guidelines by inter-professional teams in practice) and highlight lessons learned during the practice change process	Elderly patients	Mixed methods	Educational approach	Falls prevention strategy training workshops to clinical inter-professional teams – content based on guidelines developed by American Geriatric Society and British Geriatric Society	Hospitals and community or primary care clinics	Macro	Process measure: Adherence to falls prevention strategies<br><br>Outcome measure: Rate of falls, emergency department visits for falls, and hospitalisations for falls over a three month period
**Foged 2018 (39)**	Denmark	Describe nurse perspectives on how an e-message system supports communication on medication administration between hospital and home care nurses	Elderly	Qualitative	Integrated digital record	E-message system - admission report, care plan, discharge report, discharge notification and a message platform to bolster communication between hospital nurses and nurses providing home-based care	Hospital and community (home)	Macro	Qualitative outcome: The ability of an E-message system to support information exchange to assure medication safety
**Hendriks 2008 (44)**	Netherlands	Assess whether a MDT fall-prevention program was more effective than standard care in preventing new falls and functional decline in elderly community-dwelling people	Adults, 65 years and over	RCT	Care pathway	MDT in hospital providing a falls risk assessment, with referral to GP depending on risk and occupational therapy visits for a short period at home	Hospital and community (home)	Micro	Outcome, Primary measure: number of falls experienced in 12 month follow up period

		who attended an emergency department after a fall							
**Hiem 2016 (46)**	Netherlands	Decrease the risk of functional decline and loss of independence after hospitalisation in frail older patients	Elderly patients	Action research	Care pathway	Various approaches across primary and acute care to support frail patients. Approaches address (i) improved risk management; (ii) delivery of integrated, function-oriented care; (iii) specific geriatric approaches; and (iv) optimisation of transfers	Primary care, nursing home, rehabilitation centres and hospitals	Macro	Outcome: Incidence of adverse outcomes including falls in the last six months
**Johansen 2018 (40)**	Norway	Explore the effects of the intervention on healthcare use, health-related quality of life and medication appropriateness in elderly patients	70 yrs and over	Protocol RCT	Care pathway	Integrated medicines management model adapted to improve both medication safety in hospital and communication across primary and secondary care. Medication reconciliation (admission), medication review (during hospital stay), patient counselling (medicines use), medication list with explanations in discharge summary and post-discharge phone calls to primary care	Secondary and primary care	Meso	Secondary process measure: medication appropriateness, medication changes,<br><br>Outcome measure: hip fracture rate, medication-related readmissions

**Khoo 2013 (47)**	Singapore	Reduce adverse drug events resulting from high-alert medication	Adults on 'high risk of adverse events' medications	QI case study	Care pathway	Multicentre multidisciplinary collaborative focused on improving safe use of high-alert medication. QI approach with tracking of improvement conducted with an adapted IHI Trigger Tool method	Primary and acute care institutions	Macro	Outcome measure: Preventable or potentially preventable adverse drug events
**Laugaland 2012 (15)**	Global	Identify and evaluate the effects of the interventions in terms of effectiveness and efficiency of care processes in the context of effects of discharge interventions on patient safety	Patients 65 years and over	Review	Care pathway	Several studies that employed MDT approaches to transitional care to improve patient safety	Hospital, community and primary care	Meso	Process and outcome measures associated with adverse events
**Lenander 2015 (45)**	Sweden	Evaluate participants' perceptions of the SÄKLÄK project, which aims to enhance medication safety, especially for elderly patients, in primary care	Elderly	Qualitative study	MDT approach to care delivery in community (home) or primary care setting	SÄKLÄK intervention model involves a multidisciplinary group with self-assessment, peer review, feedback and agreement for change	Primary care	Meso	Qualitative outcome: Usefulness of self-assessment tool in identifying areas of improvement for medication safety

**Mckenzie 2017 (28)**	US	Describe an inter-professional team-based training approach to falls prevention for older adults and the related educational outcomes	Older adults	Service evaluation	Educational approach	Project that involves an inter-professional teaching team to support practice teams to reduce falls in older adults via implementation of evidence-based practice guidelines	Ambulatory, long term care, hospital, and home health practice	Macro	Process measure: Associated with educational outcomes resulting from falls training workshops; knowledge, learner confidence, intention for collaborative practice change and change to practice (falls screening rates and falls risk assessments)
**McQuaid-Bascon 2018 (32)**	Canada	An overview of a recently integrated health care system's experience in evolving their multi-component inter –professional approach to falls prevention	Older patients susceptible to falls	Service evaluation	Care pathway	Establishment of multidisciplinary falls committee to provide leadership, direction and coordination in the inter-professional advancement of quality improvement, evidence-informed practices and research associated with falls prevention and injury minimisation	Hospital	Meso	Process measure: i) sustain falls risk assessment and falls communication bundle across all areas; ii) monitor and report falls assessment rates and bundle implementation on inpatient areas; and iii) explore patient video monitoring and soft cell flooring<br><br>Outcome measure: improve falls with injury rates by 25%;
**Mikolaizak 2016 (49)**	Australia	Determine whether an approach involving timely assessment and tailored interventions, resulted in a meaningful reduction in subsequent falls, fall-related injuries and associated emergency health service use in older people who were	Older adults 65 years and over	RCT	MDT approach to care delivery in community (home) or primary care setting	Targeted identified risk factors and used existing services to implement physiotherapy, occupational therapy, geriatric assessment, optometry and medication management approaches to prevent falls	Community (home)	Micro	Outcome measure: Rate of falls and fall-related injuries during the 12-month follow-up period

		not transported to hospital following a fall							
**Peeters 2007 (41)**	Netherlands	Reduce the fall risk in older persons with a high risk of falling. The intervention consists of a systematic assessment of the putative causes of falling and subsequent targeted individualised preventive measures	Older adults >65	RCT	Care pathway	Assessment by geriatric outpatient clinic for falls risk factors in collaboration with a MDT that provides multifactorial treatment that may include physiotherapy, ophthalmic assessment and medicines management	Community (home)	Micro	Process measure: proportion assessed for; risk of recurrent falling, fall history, medical history, medication use, independence in activities of daily living Outcome measure: Time to first and second fall
**Robbins 2013 (29)**	US	To describe the process followed and outcomes achieved from participating in a pharmacy clinical services and patient safety collaborative and integrating pharmacy services into a high-risk, ambulatory population	Adults	QI case study	Care pathway	Patient Safety and Clinical Pharmacy Services Collaborative change package involving a MDT	Health centre	Macro	Outcome measure: Percentage of adverse drug events detected in the population of focus that received clinical pharmacy services during the month of follow up
**Sze 2008 (48)**	Hong Kong	To investigate the efficacy of a falls prevention clinic and a community step-down programme in	Elderly patients	Prospective cohort	MDT approach to care delivery in community (home) or	12 week falls clinic provided by a MDT - including fall evaluation, balance training, home hazard	Community (home)	Micro	Primary outcome measure: Number of falls per person per year<br><br>Secondary outcome measure: Fall-related injuries

		reducing the number of falls among community-dwelling elderly at high risk of a fall			primary care setting	management program, and medical referrals. Followed by a community step-down (nine months) program, including falls prevention education, a weekly exercise class, and two home visitations			
**Toivo 2019 (42)**	Finland	To assess the impact of a care coordination intervention on medication risks identified in drug regimens of older home care clients over a one-year period	Elderly patients	Cluster RCT	MDT approach to care delivery in community (home) or primary care setting	Coordinated medication risk management (CoMM) model which involves a multi-stage approach; triage, medication risk screening and reviews, actions plans delivered by a MDT	Community (home)	Micro	Process measure: Several including use of harmful medications<br><br>Outcome: clinically significant drug interactions
**Van Melle 2018 (43)**	Netherlands	To pilot a review of medical records to identify transitional safety incidents (TSIs) for use in a large intervention study and assess its reliability and validity.	Adults under cardiology and gastro service care	Retrospective medical record review study	Integrated digital record	Transitional medical record system: establish inter-rater reliability of a measurement tool designed to assess transitional safety incidents	Hospital and primary care	Macro	Outcome measures: Transitional safety incidents which include but not restricted to adverse drug events leading to unplanned hospital admission
**Wallace 2018 (30)**	US	To evaluate the effects of a multidisciplinary hip fracture care pathway on the outcomes of the elderly patients	Elderly	Retrospective analysis	Care pathway	Multidisciplinary care pathway for reduce hip fractures in the elderly	Hospital (Trauma centre)	Micro	Outcome measures: Several – includes mechanism of injury (such as falls), hip fracture characteristics and hospital length of stay

**Willis 2011 (31)**	US	To determine if trained undergraduate students, in conjunction with pharmacists, could provide in home medication reviews, and demonstrate benefit to the health and welfare of a senior population affiliated Patient-Centred Medical Home	Patients aged 65 years and over	Service evaluation	MDT approach to care delivery in community (home) or primary care setting	Patient-Centred Medical Home model - In-home medication reviews, assessing a home for fall risk, and performing blood pressures	Community (home)	Meso	Process measures: Proportion of patients prescribed falls risk medication and medicines that have a high potential for major drug interaction. Proportion of patients that have had a medication change made to their clinical medication list
