# Supplementary material for: Approaches to improving patient safety in integrated care: a scoping review
Source: BMJ Open. 2023 Apr 4;13(4):e067441. doi: 10.1136/bmjopen-2022-067441 (PMC10083780; doi:10.1136/bmjopen-2022-067441)
Supplement: Author's manuscript [file bmjopen-2022-067441.draft_revisions.pdf]

BMJ Open

BMJ Open is committed to open peer review. As part of this commitment we make the peer review history of every article we publish publicly available.

When an article is published we post the peer reviewers' comments and the authors' responses online. We also post the versions of the paper that were used during peer review. These are the versions that the peer review comments apply to.

The versions of the paper that follow are the versions that were submitted during the peer review process. They are not the versions of record or the final published versions. They should not be cited or distributed as the published version of this manuscript.

BMJ Open is an open access journal and the full, final, typeset and author-corrected version of record of the manuscript is available on our site with no access controls, subscription charges or pay-per-view fees (http://bmjopen.bmj.com).

If you have any questions on BMJ Open's open peer review process please email info.bmjopen@bmj.com

BMJ Open

Approaches to improving patient safety in integrated care: a scoping review

Journal:	*BMJ Open*
Manuscript ID	bmjopen-2022-067441
Article Type:	Original research
Date Submitted by the Author:	05-Sep-2022

Complete List of Authors:	Lalani, Mirza ; London School of Hygiene and Tropical Medicine Department of Health Services Research and Policy<br>Wytrykowski, Sarah; Medical Research Network<br>Hogan, Helen; London School of Hygiene & Tropical Medicine, Health Services Research and Policy

Keywords:	Organisation of health services < HEALTH SERVICES ADMINISTRATION & MANAGEMENT, Quality in health care < HEALTH SERVICES ADMINISTRATION & MANAGEMENT, HEALTH SERVICES ADMINISTRATION & MANAGEMENT

SCHOLARONE™
Manuscripts

I, the Submitting Author has the right to grant and does grant on behalf of all authors of the Work (as defined in the below author licence), an exclusive licence and/or a non-exclusive licence for contributions from authors who are: i) UK Crown employees; ii) where BMJ has agreed a CC-BY licence shall apply, and/or iii) in accordance with the terms applicable for US Federal Government officers or employees acting as part of their official duties; on a worldwide, perpetual, irrevocable, royalty-free basis to BMJ Publishing Group Ltd ("BMJ") its licensees and where the relevant Journal is co-owned by BMJ to the co-owners of the Journal, to publish the Work in this journal and any other BMJ products and to exploit all rights, as set out in our licence.

The Submitting Author accepts and understands that any supply made under these terms is made by BMJ to the Submitting Author unless you are acting as an employee on behalf of your employer or a postgraduate student of an affiliated institution which is paying any applicable article publishing charge ("APC") for Open Access articles. Where the Submitting Author wishes to make the Work available on an Open Access basis (and intends to pay the relevant APC), the terms of reuse of such Open Access shall be governed by a Creative Commons licence – details of these licences and which Creative Commons licence will apply to this Work are set out in our licence referred to above.

Other than as permitted in any relevant BMJ Author's Self Archiving Policies, I confirm this Work has not been accepted for publication elsewhere, is not being considered for publication elsewhere and does not duplicate material already published. I confirm all authors consent to publication of this Work and authorise the granting of this licence.

For peer review only

Approaches to improving patient safety in integrated care: a scoping review

Mirza Lalani,[1*] Sarah Wytrykowski,[2] Helen Hogan[1]

[1] Department of Health Services Research and Policy, London School of Hygiene and Tropical Medicine

[2] Medical Research Network

*Corresponding author: Mirza Lalani, mirza.lalani@lshtm.ac.uk

For peer review only

Abstract

Objectives: This scoping review aimed to establish the approaches employed to manage patient safety in integrated care for community dwelling people with long term conditions

Design: Scoping Review

Setting: All care settings

Search strategy: Systematic searches of seven academic and grey literature databases for studies published between 2000-2021. At the full text review stage both the first and second reviewer (SW) independently assessed full texts against the eligibility criteria and any discrepancies were discussed

Results: Overall, 24 studies were included in the review. Two key priorities for safety across care boundaries for people with LTC were falls and medication safety. Interventions for these priorities were implemented at different levels of an integrated care system (ICS). At the micro level, interventions involved patient-centred care primarily in the home setting provided by multi-disciplinary teams. At the meso-level, the focus was on planning and designing approaches at the managerial/organisational level to deliver multi-disciplinary care. At the macro level, system-wide approaches included integrated care records, training and education and the development of care pathways involving multiple organisations.

Conclusions: Evaluation of these interventions was undertaken using a wide range of process and outcome measures to capture patient harm and contributory factors associated with falls and

For peer review only - http://bmjopen.bmj.com/site/about/guidelines.xhtml

medication safety. For integrated care initiatives to fulfil their promise of improving care for people with LTC, interventions to improve safety need to be instituted across the system, at all levels to support the structural and relational aspects of integrated care as well as specific risk-related safety improvements.

Keywords: Patient safety, integrated care, falls, medication safety

Word limit: 4438

Strengths and limitations:

- Highlighting how key components of integrated care can also improve safety across care boundaries particularly in reducing falls and medication safety management
- Another strength of the study was the rigorous approach to scoping and selecting the literature including the comprehensive searching of several academic and grey literature databases and sources
- Nonetheless, the identification of relevant studies was possibly hindered by the lack of consistency and interchangeability of the terms ‘integrated care and patient safety’ and their use in the literature.
- While the review was able to identify the different types of safety interventions (scope) it was more challenging to describe their scale across care boundaries.

For peer review only - http://bmjopen.bmj.com/site/about/guidelines.xhtml

Introduction

Health and social care systems globally are facing the challenges of an ageing population with complex co-morbidities that require multi-faceted care provision to better manage their health and care needs. [1] Care provision for people (particularly older and frail) with long term conditions (LTC) can be fragmented, impacting the quality of care, with a greater potential for adverse outcomes. [2] The integration of health and social care systems and services is seen as integral to tackling these challenges. [3] The term 'integration' broadly represents a 'joining up' of traditional silos of care across (horizontal) and within (vertical) systems, organisations and services. Integrated care transcends longstanding professional, organisational and regulatory boundaries to provide more coordinated, collaborative and person-centred care. [4-6] Integrated care may contribute to improving the safety of care for people with LTC by addressing the risks posed by multiple referrals, handovers and discharges that arise as the responsibility for care crosses care boundaries and involves different practitioners, teams and organisations. [7] Many high-income countries have taken steps to delivery integrated care through integrated care systems (ICS) which involve the collaboration of sectors and care providers, bringing together primary and secondary healthcare, social care, community and mental health services and voluntary organisations. [8]

The World Health Organisation (WHO) defines patient safety as 'the absence of preventable harm to a patient during the process of health care and reduction of risk of unnecessary harm associated with health care to an acceptable minimum.'[9] Risks to patient safety can be mitigated within ICS by strengthening some of the key structural and relational aspects of service provision such as communication, partnership working, care coordination and person-centred care as well as intervening to reduce specific harms. [6]

For peer review only - http://bmjopen.bmj.com/site/about/guidelines.xhtml

In recent years, many integrated care initiatives have focussed on shifting care away from hospitals and closer to home, an approach that is not without risk. Adverse events in the community setting cause between 8-12% of hospital admissions, around a half of which are thought to be preventable. [10,11]. Harm in this setting may result from medication mismanagement, poor infection control, failure to ensure safe mobilisation or a lack of prevention of and care for pressure areas. [12] Studies have also highlighted that the 'spaces' between care settings are prone to problems of communication and coordination which affect the safety of care as patients transition across care boundaries and receive care from different professionals. [13,14]

Recent approaches to better management, assurance and improvement of safety have advocated for developing safety governance through a whole systems approach addressing safety issues at the micro (clinical team), meso (organisation) and macro (system) levels of a care system. [15] Similarly, models of integrated care have developed structures to support care delivery at these different levels. [16]

Currently, there is a lack of knowledge on the scale and scope of safety interventions for older people in ICS and how these operate at different levels. This scoping review aims to address this gap by establishing the approaches employed to manage patient safety in integrated care for community dwelling people with LTC and how these are evaluated. Such assessment will gauge current key priorities for safety within integrated care and the types of safety measures that may be useful for ongoing monitoring of system safety.

For peer review only

Methods

This scoping review was conducted in accordance with the PRISMA extension for scoping reviews (PRISMA-ScR) guidance.[17] A protocol was developed but not published. We used the first five stages of Arksey and O'Malley's framework in undertaking this scoping review; (i) identifying the research

For peer review only - http://bmjopen.bmj.com/site/about/guidelines.xhtml

question; (ii) identifying relevant studies; (iii) selecting studies; (iv) charting the data; (v) collating, summarising and reporting the results. As the relevant literature was likely to be extensive, complex and potentially heterogeneous, a scoping review was selected over a systematic review. [18]

Stage 1: Identifying the research questions

The scoping review explored the breadth of literature to answer the research question, 'What type of integrated care approaches exist at different system levels to improve safety for community-dwelling people with LTC and how have they been assessed?'

Stage 2: Identifying relevant studies

Free text search terms were developed based on our research question. The search term strategy (supplementary file 1) was broadly based on three blocks of terms; 1) population, 2) intervention (associated with integrated care), and c) outcome (related to patient safety).

A comprehensive search strategy was employed to include both the peer-reviewed and grey literature. Seven electronic databases were searched including MEDLINE, Social Policy and Practice, Web of Science, CINAHL, Google Scholar, Proquest and OpenGrey. These were supplemented by forward and ancestry citation searches using reference lists of several relevant studies.

Stage 3: Selecting studies

Study selection was based on the inclusion and exclusion criteria listed in table 1.

Table 1: Overview of exclusion and inclusion criteria

**Inclusion criteria**	
Organisational setting	All types i.e. acute, primary, community, mental health and social care
Population	People aged 65 and over
Intervention	Involved at least two different types of organisations e.g. hospital and community i.e the initiative under assessment spanned different sectors
Outcome	Associated with patient safety as defined by the WHO
Study design	Primary and/or secondary data (research studies, quality improvement project reports, service evaluations and reviews)

For peer review only - http://bmjopen.bmj.com/site/about/guidelines.xhtml

Time interval	2000-2021 - We selected 2000 as a start date because it coincides with a proliferation in safety-related research stimulated by the publication of the seminal report; To Err is Human: Building a Safer Health System. (21)
	Studies published in English only
**Exclusion criteria**	
Organisational setting	Transitions of care within the same sector e.g. hospital only<br>Studies in which it is challenging to discern whether staff in an MDT were employed by different providers and therefore part of an integrated care team
Type of study	We also excluded commentaries and editorials.

The first reviewer (ML) undertook initial searches and based on information in the title and abstract selected those meeting criteria for full text review. At the full text review stage both the first and second reviewer (SW) independently assessed full texts against the eligibility criteria and any discrepancies were discussed.

Stage 4: Charting the data

The first reviewer extracted and tabulated the data from the included studies in Microsoft Excel. Details of the studies extracted included the overall aim, population, study design, level of system, intervention approaches, setting and approach to measuring safety. The second reviewer reviewed and verified the data extraction table. Descriptions of the levels of the system i.e. micro, meso and macro and the approach to measuring safety (outcome, process and qualitative) [19] are outlined in box 1. The nomenclature for different levels of a system (micro, meso and macro) can be ascribed to both ICS and safety system governance. [15 16] The Donebedian framework for measuring quality in health care applies to identifying measures for safety i.e. outcome and process, in the case of this review. [20] We have also added a further measure, 'qualitative' to capture perceptions of study participants regarding service improvements associated with safety through implementation of interventions.

For peer review only - http://bmjopen.bmj.com/site/about/guidelines.xhtml

Box 1: Description of the different levels of a care system

<u>Levels of care system:</u>

Micro-level: Typically comprises an approach at the patient level where multiple professionals from different types of organisations provide care commonly in the home setting but sometimes in primary care alone or in primary and secondary care if linked by a common pathway.

Meso level: Interventions at management/organisational level that involve multi-professionals in planning or designing local structures and services that span two or more different types of organisations.

Macro level: System wide interventions that may involve several providers with a typical focus on strategy, infrastructure development and other wide-ranging cultural and educational initiatives. Interventions that are typically implemented over a wider a geography within a country e.g. region, county, state etc.

<u>Approaches to measuring safety:</u>

Outcome measures: Indicate the impact of a health care service or intervention on the patient's health status.

Process measures: Practices and approaches delivered in order to achieve the desired outcome of an intervention.

Qualitative outcomes: Perspectives of study participants on the implementation and/or impact of an intervention (primarily from qualitative studies only).

Quality assessment

Due to the heterogeneity of the studies in terms of their methodological design, settings in which an intervention was implemented as well the design and scope of the intervention itself, and in line with similar scoping reviews, formal quality assessment was not undertaken. [21 22]

Patient and Public Involvement

No patients or members of the public were directly involved in this work. However, this study is part of a wider programme of research for which there is a research advisory group whose membership includes patients/public representatives. In the conceptualisation phase of the study, the research advisory group provided advice and guidance on the study's aims and purpose.

For peer review only - http://bmjopen.bmj.com/site/about/guidelines.xhtml

Results

Stage five: collation, summarising and reporting

The original searches yielded 2957 records. After removing duplicate records and screening of titles and abstracts, 86 were allocated for full text review. After application of eligibility criteria, 24 studies were included in the review. The flow of studies from initial identification to final inclusion are presented in figure 1.

Figure 1: Flowchart of study selection

General characteristics of included studies

All included studies were published between 2007 and 2019. We identified one review [12] and 23 empirical studies. A breakdown of the study characteristics is provided in the supplementary table. In terms of geographical distribution, empirical studies were undertaken in North America [23-28], Europe [29-41] Asia [42 43] and Australia [44]. All studies involved participants described as elderly, older adults or adults over the age of 65, although four studies also included other adults aged over 18. In all studies, the population in receipt of interventions were reported to have long-term, chronic physical or mental health conditions. However, in a few of these studies, a health condition(s) was not specified and instead participants subject to the intervention were described as frail or vulnerable. Empirical studies comprised the majority of study types and included randomised control trials (RCT), [33 34 36 38 39 44 45] qualitative studies, [31 32 35 46] mixed methods studies, [23 30] retrospective analysis of medical records, [26 40] a cohort study, [43] a cross-sectional study, [29] an observational study,[32] a systematic review [12] and action research. [47] Service evaluations [24 27 28] and quality improvement (QI) studies [25 42] were also among the list of included studies.

Types of risks addressed

For peer review only

For peer review only - http://bmjopen.bmj.com/site/about/guidelines.xhtml

Of the 24 included studies, 11 involved interventions designed to reduce falls risk, [23 24 28 30 33 34 38 43-45 47] and ten studies were broadly associated with assuring medication safety through reconciliation and review, surveillance and monitoring [25 29 31 32 35 36 39 40 42 46]. Three studies undertook a series of approaches to reduce adverse events more broadly such as identifying medications that increase the risk of a fall in older people and an assessment of an intervention designed to reduce the rate of hip fracture. [26 27] The systematic review included studies that involved interventions to reduce adverse events including ADRs. [12]

Intervention approaches

Setting

The studies included a range of intervention settings. A total of 13 studies described interventions implemented in a single setting; the home setting [27 30 31 34 38 39 43 44] and the remainder in hospitals, [26 28 32] community health centres, [25] and primary care. [46] In the remaining studies, a care pathway was established either across several settings [12 24 42 47] or just two settings; between the hospital and home setting [33 35 45] or hospital and primary care.[23 29 36 40]

Level of care system

Various interventions were implemented at different levels of the care system; micro, meso and macro. Ten studies were at the micro level, [26 30 32-34 38 39 43-45] five at the meso level [12 27 28 36 46] and nine at the macro level [23-25 29 31 35 40 42 47]. Broadly, these interventions can be categorised into four types; i) MDT approach to care delivery in community (home) or primary care setting, ii) care pathway, iii) educational intervention and iv) integrated digital record (accessible to different care providers).

Micro level

At the micro level, interventions were characterised by the delivery of person-centred coordinated care for people with LTC involving the home (community) setting or primary care setting alone or as

For peer review only - http://bmjopen.bmj.com/site/about/guidelines.xhtml

For peer review only

part of a care pathway with secondary care settings. Care was provided by a range of health and care professionals working together. Three studies outlined multidisciplinary approaches to prevent and reduce falls, primarily in the home setting. [30 34 44] Interventions included exercise therapy, home based assessments for hazards with accompanying modifications, vision assessment, home medication review and a 24 hour call service staffed by a home intervention team.

A further five studies described interventions implemented across different parts of a care pathway to reduce falls, although *Wallace et al* focussed on the development of a multi-disciplinary care pathway following a hip fracture. [26 33 38 43 45] Interventions comprised multi-disciplinary outpatient clinics (often located in hospitals) where patients were assessed and monitored, with further follow-up care provided by community services staff in the home setting. [33 38 43 45] Follow up care included assistance with daily living, medication review, balance and cognition, [33 43 45] as well as promotion of patient/user independence through home hazard (home environment risk) assessments, [38 43 45] exercise to improve mobility and strength, [33 38 43] and patient education on use of assisted devices to support individuals to navigate their home environment. [33]

Three studies involved medicines reviews to reduce the risk of adverse drug events (ADE) including the reduction of falls. [27 39 46]

Meso-level

At the meso-level interventions were mainly focused on multi-professional management approaches to planning and designing services across at least two different types of organisation, primarily hospitals and community care. Interventions tackled areas with overarching safety risks such as poor communication or dispersed leadership. All five studies used different intervention models.

Three studies aimed to improve medication safety for older people. One study employed an Integrated Medicines Management (IMM) model (spanning hospitals and primary care) which comprised medicines reconciliation and review, patient counselling on medicines usage during

For peer review only - http://bmjopen.bmj.com/site/about/guidelines.xhtml

hospital stay and greater provision of information and communication with primary care at discharge and post-discharge. [36] Another study described an intervention model in primary care involving managers, pharmacists and nurses who engaged in self-assessment, peer review, feedback and agreement for change to improve medicines safety management. [46] *Willis et al* described an MDT approach involving medication reviews, home fall risk assessments and blood pressure monitoring. [27]

A further study, outlined a multidisciplinary falls committee (within a newly created ICS) whose role was to provide leadership, direction and coordination to frontline staff promoting new evidence based practices and quality improvement support to prevent falls. [28] The systematic review described interventions such as education and training, organisational/culture interventions (e.g. discharge protocol and planning, medication reconciliation etc.), or patient/family orientated interventions concerning awareness and empowerment for patients. [12]

Macro level

At the macro level, interventions were characterised by system-wide approaches to manage medication safety and reduce falls risk. Interventions included key infrastructure developments such as integrated care records, training and education initiatives spanning several organisations within a system and creating care pathways involving multiple organisations. Interventions were generally implemented across a wider geography e.g. county, state or region.

Three studies described digital approaches to medication safety monitoring such as a digital database for patients transitioning from secondary to primary care with indicators to alert practitioners to potential ADE [29] and a digital measurement tool designed to assess transitional safety incidents affecting older people including ADE. [40] A further study described how an e-message system for nurses in hospital and community settings could support information exchange - minimising ADE. [35]

For peer review only - http://bmjopen.bmj.com/site/about/guidelines.xhtml

Two studies described educational interventions comprising training of inter-professional teams (including social workers) from a range of settings on developing strategies for preventing falls in accordance with national evidence-based guidelines. [23,24]

The remaining studies described multi-component intervention models such as quality improvement collaborative approaches, or other models comprising multi-disciplinary working, care coordination and strategies to strengthen leadership to promote patient safety and reduce hospital admission or readmission. The Quality improvement collaborative aimed to reduce the risk of ADE and involved MDTs with professionals from primary and acute care who adopted a change package involving clinical pharmacy services [25] and a quality improvement approach based on an adaptation of the Institute of Healthcare Improvement Trigger Tool method. [42]

Profile of professionals involved in the interventions

Of the 23 empirical studies, the majority included a multidisciplinary approach to care provision involving different health professional groups. Four of the interventions also included social workers as part of the team. [23,24,26,28] In eight studies, the interventions included health and social care professionals such as nurses, paramedics, managers, social workers, and allied health professionals; physiotherapists and occupational therapists, pharmacists and optometrists but doctors were not involved. [24,27,28,31-33,35] Many of these studies involved interventions implemented in the community (home setting) or where the home was included as a part of a care pathway.

Measures for assessing impact of the intervention

There was heterogeneity in the approaches to measuring safety. We categorised measures as process, outcome or qualitative.

Two studies included process measures alone. The first involved the monitoring and surveillance of medication safety including the proportion of older patients prescribed falls risk medication, and the proportion of medicines prescribed with have a high potential for major drug interaction. [27] The

For peer review only

For peer review only - http://bmjopen.bmj.com/site/about/guidelines.xhtml

second study identified measures associated with educational outcomes of health professionals participating in a falls training workshop including subsequent changes to practice. This included workshop participants self-reporting their undertaking of assessments of older patients at higher risk of falls and screening of falls rates. [24]

In 11 studies, outcome measures alone were mentioned. Four of these studies stated falls incidence during follow up over a period of 6-12 months [33 34 45] as a key measure. [47] In three further studies, falls and falls related injuries such as hip fracture, and hospital length of stay were key outcomes [26 43 44]. In four studies, ADE were stated as outcome measures including preventable ADE or potentially preventable ADE, [29 42] proportion of ADE detected in a specified population [25], and ADEs leading to unplanned hospital admission. [40]

Six studies included both process and outcome measures. Process measures associated with falls included adherence to falls prevention strategies, proportion of falls risk assessments undertaken, documenting of falls and medical history risk of falling, and level of independence in activities of daily living. [23 36 38] Outcome measures in these studies included decrease in falls rates, time between the first and second falls, falls related subsequent emergency department visits and hospitalisation.

Process measures associated with medication safety comprised medication appropriateness and use of harmful medications. [28 39] Outcome measures in these studies were medication related readmissions, rate of hip fractures and clinically significant drug interactions. The systematic review described a range of process and outcome measures across the included studies, all associated with adverse events including ADE. [12]

The remaining studies described qualitative outcomes for the integrative approaches i.e. based on the perceptions of study participants. These included assessing interventions designed to prevent falls such as the perceived usefulness of a MDT home-based fall prevention programme. [30] A further four studies described qualitative outcomes in terms of assessing the quality of medication safety management practices. These included the extent of professional learning experiences to support

For peer review only - http://bmjopen.bmj.com/site/about/guidelines.xhtml

medicines management, [31] the quality of service delivery of a domiciliary hospitalisation unit and its impact on assuring drug safety, [46] the usefulness of a self-assessment tool in identifying areas of improvement for medication safety [32] and the ability of an E-message system to support information exchange to assure medication safety. [35]

Discussion

This scoping review brings together the published literature on approaches to improve safety in integrated care for community-dwelling people with LTC and demonstrates the range of interventions employed in safety improvement strategies for care across boundaries within several countries. The interventions identified in the included studies focus primarily on two common patient safety targets for harm, falls and ADE through falls prevention and medication safety programmes spanning micro, meso and macro levels of a health and care system. Just over a third of the included studies involved the implementation system-wide approaches (macro level) such as digital shared care records, training/educational initiatives and the strengthening of care pathways between providers. A higher proportion (40%) of studies described micro-level interventions, primarily MDT delivering care to older people in the home setting alone or as part of a care pathway involving hospitals and primary care. The review also highlighted that the evaluation of interventions indicated a perspective of safety focused primarily on measurement - changes in rates of adverse events or contributory factors at individual patient level.

A key strength of this study was the highlighting of how key components of integrated care can also improve safety across care boundaries particularly in reducing falls and medication safety management. Instituting integrated care practices at the different levels of a care system e.g. digital shared care records (macro), strengthening of care pathways (macro, meso and micro) and promoting MDT working (meso and micro) will strengthen safety approaches and reduce the risk of harm across an ICS. Furthermore, the study identified a range of measures to assess safety across systems suggesting that ICS will need to develop a range of indicators to capture the variation in

For peer review only - http://bmjopen.bmj.com/site/about/guidelines.xhtml

performance of key processes that lead to desired safety outcomes. Another strength of the study was the rigorous approach to scoping and selecting the literature including the comprehensive searching of several academic and grey literature databases and sources. In comparison to the existing literature on this topic, our study builds upon previous work on safety in care transitions but extends beyond the spaces and pathways between organisations at the micro level to consider system-wide (meso and macro) approaches to improving safety.

The study was not without limitations. Articulating a precise definition for patient safety was challenging, as, in several studies, the term was associated with preventing hospitalisation. Hence, we selected the WHO definition which is broad ranging and encompasses the notion of errors of commission and omission. Nonetheless, the identification of relevant studies was hindered by the lack of consistency and interchangeability of the terms 'integrated care and patient safety' and their use in the literature. We may have screened for word choice rather than presence or absence of a similar term or its description. Despite including a range of search terms associated with safety some studies may have been missed. Our search strategy did not include social care as a specific search term, although integrated care including care coordination and multidisciplinary working is increasingly involving social care professionals. While the review was able to identify the different types of safety interventions (scope) it was more challenging to describe their scale across care boundaries.

Integrated care initiatives identified in this study involved the design of a range of new structures and processes to support the delivery of safer care, although further work is required to establish their effectiveness in improving patient safety. Integrated digital care records have been cited as a key enabler for structural integration in ICS as they promote sharing of information thus minimising the risk of care-related information being overlooked as people (particularly older and more frail) move along a care pathway. [48] Training and educational initiatives delivered to inter-professional teams are also considered to be a means of improving the quality of integrated care delivery,

For peer review only - http://bmjopen.bmj.com/site/about/guidelines.xhtml

supporting the development of social and working relationships thereby promoting partnership working. [49] At all levels, several interventions were implemented to strengthen the care pathway on discharge from hospital and provide tailored care in the home setting to reduce the risk of adverse events. These initiatives had several goals associated with integration, to deliver care closer to home, reduce duplication, minimise the risk of hospital admission and provide personalised care. [3] Each of these goals are notable processes of integration which, if successfully implemented, could also improve safety outcomes. These focused approaches do not negate the need for further efforts to develop safety systems within ICS including safety governance at all levels, system-wide mechanisms to identify, review and learn from safety incidents, enhanced capacity and capability for measurement and improvement, and creating learning cultures that promote openness and transparency. [15 50]

The levels of the system (micro, meso and macro) approach to identifying safety interventions presented in this study provides a useful means of understanding how organisations can implement change, improvement and innovation in an across an ICS. At the macro level, the implementation of integrated care records to collate information and support communication alongside the promotion of a learning culture supported by inter-professional training and education is required. At the meso level, the focus would be on organisations jointly planning and designing multi-disciplinary approaches to develop seamless care pathways, minimising the risk of harm as people transition between providers. At the micro-level, a range of multi-disciplinary working practices to improve safety could be deployed from near real time safety monitoring within 'community huddles' to protected time sessions to compare and reflect on performance across teams promoting longer term changes. [51] These approaches are potential solutions to the fragmentation of health and care systems (which continues despite efforts to integrate care) and will be integral to improving outcomes for increasing populations of people with LTC as they receive care closer to home. [15]

For peer review only - http://bmjopen.bmj.com/site/about/guidelines.xhtml

The studies included in this review evaluated falls reduction and medicines safety interventions using clinical process and outcome measures with several authors looking at associations between these outcomes and wider health system outcomes e.g. hospital (re) admissions and length of stay. System outcome measures have been widely used as performance indicators to assess the impact of integrated care. [3 52] While structural or relational measures of integration such as collaborative and co-terminus working [53-55] or the aforementioned system outcomes dominate the discourse of the effects of integrated care, less attention has been paid to measures related to patient or carer experience. [56] None of the studies in this review collected data on the experience of patients and carers alongside clinical outcomes. The understanding of experiences of patients in receipt of services may support service improvement to deliver better quality care. [57 58]

A wide range of process and outcome measures were used across the studies predominantly to capture patient harm and contributory factors. Expanding measurement to incorporate the capture of the variation in performance of key processes that lead to desired outcomes, would not only identify the most effective interventions for improving patient safety but also open up a wider range of intervention options to support building resilience across ICS. [59] A standardised set of safety indicators would support comparisons between different integrated care approaches to safety.

Safety interventions in this review were mainly focused on medication safety management and falls prevention which is unsurprising as both account for a notable proportion of unplanned hospital admissions. In England, in 2018 falls accounted for a total of 220,160 hospital admissions in those aged over 65. A study of medication errors in the UK estimated that ADE accounted for 27,362 hospital admissions annually, relating to 136,811 bed days at a cost of £83.7 million to the NHS. [60 61] While falls and medication safety are justifiably key safety priorities for integrated care, there is scope to broaden the focus to other common safety issues. For example, a study in the Netherlands found that of 106 admissions to hospital in patients aged over 65 over a period of 12 months were

For peer review only - http://bmjopen.bmj.com/site/about/guidelines.xhtml

due to an adverse event with 45% were due to procedural related infections, community acquired infection such as pneumonia with/without signs of sepsis and diabetes related hypoglycaemia. [61 62]

Future research should focus on the role of other patient safety targets (not just falls and medication related safety) that increase the risk of harm for people with LTC especially in the community setting. Furthermore, research must establish the effectiveness of the structural and relational aspects of integrated care such as care coordination as well as the approaches identified in this review e.g. integrated care records and education and training, in terms of their impact on reducing the risk of harm across care pathways. This would provide the evidence for policymakers and system leaders to implement the necessary changes to support integration, safety governance and service improvement.

Conclusion

In order to maximise the potential for ICS to fulfil their promise of improving care for people with LTC, interventions such as integrated care records, allocating resources for training and education, greater efforts to form robust and risk-limited care pathways as well as support for MDT working, especially in the community setting will have to be key priorities for improving safety across systems. They also align to the goals of ICS thereby reducing fragmentation and enhancing personalisation of care.

Data availability statement

Data sharing not applicable as no datasets were generated and/or analysed for this study. All data relevant to the study are included in the article or uploaded as online supplemental information.

Ethics approval statement

No ethical approval was needed because data from previous published studies are being used.

For peer review only - http://bmjopen.bmj.com/site/about/guidelines.xhtml

Funding

This research is funded by the National Institute for Health Research (NIHR) Policy Research Programme, conducted through the Quality, Safety and Outcomes Policy Research Unit, PR-PRU-1217–20702. The views expressed are those of the author(s) and not necessarily those of the NIHR or the Department of Health and Social Care.

Competing interests

None.

Author Contributions

ML conceived and designed the study. All authors contributed to several aspects of the review process i.e. stages 1-5 described in the manuscript under the methods section. ML drafted the manuscript. All authors approved the final version of the manuscript.

References

1. Armitage GD, Suter E, Oelke ND, et al. Health systems integration: state of the evidence. *Int J Integr Care* 2009;9(2):e82. doi: 10.5334/ijic.316 [published Online First: 2009/07/11]
2. Stange KC. The problem of fragmentation and the need for integrative solutions. *Annals of family medicine* 2009;7(2):100-03. doi: 10.1370/afm.971
3. Baxter S, Johnson M, Chambers D, et al. The effects of integrated care: a systematic review of UK and international evidence. *BMC health services research* 2018;18(1):350. doi: 10.1186/s12913-018-3161-3
4. Ham C, Dixon J, Chantler C. Clinically integrated systems: the future of NHS reform in England? *BMJ (Clinical research ed)* 2011;342:d905. doi: 10.1136/bmj.d905 [published Online First: 2011/03/30]
5. Waring J, Marshall F, Bishop S. Understanding the occupational and organizational boundaries to safe hospital discharge. *Journal of Health Services Research and Policy* 2015;20(1 Suppl):35-44. doi: 10.1177/1355819614552512 [published Online First: 2014/12/05]
6. Glasby J. If Integration Is the Answer, What Was the Question? What next for English Health and Social Care Partnerships? *Int J Integr Care* 2016;16(4):11. doi: 10.5334/ijic.2535 [published Online First: 2017/03/21]
7. Waring J, Bishop S. Health States of Exception: unsafe non-care and the (inadvertent) production of 'bare life' in complex care transitions. *Sociology of Health and Illness* 2020;42(1):171-90. doi: 10.1111/1467-9566.12993 [published Online First: 2019/09/19]
8. Shaw S, Rosen R, Rumbold B. What is Integrated Care? London: Nuffield Trust, 2011:1-23.
9. WHO. Patient safety: World Health Organisation; 2021 [Available from: https://www.who.int/teams/integrated-health-services/patient-safety.
10. Illingworth J. Is the NHS getting safer? London, UK: The Health Foundation, 2015.
11. Vincent C, Burnett S, Carthey J. The measurement and monitoring of safety. London: The Health Foundation, 2013.

For peer review only - http://bmjopen.bmj.com/site/about/guidelines.xhtml

12. Laugaland K, Aase K, Barach P. Interventions to improve patient safety in transitional care--a review of the evidence. *Work (Reading, Mass)* 2012;41 Suppl 1:2915-24. doi: 10.3233/WOR-2012-0544-2915 [published Online First: 2012/02/10]
13. Aase K, Schibevaag L, Waring J. Crossing boundaries: quality in care transitions. Researching Quality in Care Transitions: Springer 2017:3-29.
14. King BJ, Gilmore-Bykovskyi AL, Roiland RA, et al. The consequences of poor communication during transitions from hospital to skilled nursing facility: a qualitative study. *Journal of the American Geriatrics Society* 2013;61(7):1095-102. doi: 10.1111/jgs.12328 [published Online First: 2013/06/03]
15. Auraaen A, Saar K, Klazinga N. System governance towards improved patient safety: OECD, 2020.
16. Curry N, Ham C. Clinical and service integration: The King's Fund, 2010.
17. Tricco AC, Lillie E, Zarin W, et al. PRISMA Extension for Scoping Reviews (PRISMA-ScR): Checklist and Explanation. *Annals of internal medicine* 2018;169(7):467-73. doi: 10.7326/m18-0850 [published Online First: 2018/09/05]
18. Peters MD, Godfrey CM, Khalil H, et al. Guidance for conducting systematic scoping reviews. *International Journal of Evidence Based Healthcare* 2015;13(3):141-6. doi: 10.1097/xeb.0000000000000050 [published Online First: 2015/07/03]
19. Improvement N. The how-to guide for measurement for improvement: NHS Improvement, 2020.
20. Donabedian A. Evaluating the quality of medical care. *The Milbank memorial fund quarterly* 1966;44(3):166-206.
21. Duhn L, Godfrey C, Medves J. Scoping review of patients' attitudes about their role and behaviours to ensure safe care at the direct care level. *Health Expectations* 2020;23(5):979-91. doi: https://doi.org/10.1111/hex.13117
22. Cheraghi-Sohi S, Panagioti M, Daker-White G, et al. Patient safety in marginalised groups: a narrative scoping review. *International journal for equity in health* 2020;19(1):26-26. doi: 10.1186/s12939-019-1103-2
23. Eckstrom E, Neal MB, Cotrell V, et al. An Interprofessional Approach to Reducing the Risk of Falls Through Enhanced Collaborative Practice. *Journal of the American Geriatrics Society* 2016;64(8):1701-7. doi: 10.1111/jgs.14178 [published Online First: 2016/07/29]
24. McKenzie G, Lasater K, Delander GE, et al. Falls prevention education: Interprofessional training to enhance collaborative practice. *Gerontology & Geriatrics Education* 2017;38(2):232-43. doi: 10.1080/02701960.2015.1127809 [published Online First: 2016/02/18]
25. Robbins CM, Stillwell T, Johnson D, et al. Integrating patient safety and clinical pharmacy services into the care of a high-risk, ambulatory population: a collaborative approach. *Journal of patient safety* 2013;9(2):110-7. doi: 10.1097/PTS.0b013e318281b879 [published Online First: 2013/05/24]
26. Wallace R, Angus LDG, Munnangi S, et al. Improved outcomes following implementation of a multidisciplinary care pathway for elderly hip fractures. *Aging clinical and experimental research* 2019;31(2):273-78. doi: 10.1007/s40520-018-0952-7 [published Online First: 2018/04/25]
27. Willis JS, Hoy RH, Jenkins WD. In-home medication reviews: a novel approach to improving patient care through coordination of care. *Journal of community health* 2011;36(6):1027-31. doi: 10.1007/s10900-011-9405-3 [published Online First: 2011/04/19]
28. McQuaid-Bascon K, Royal M, Sinno M, et al. Evolving a multi-factorial, data driven, interprofessional approach to prevent falls and associated injuries during a system-level integration. *Journal of Interprofessional Education* 2018;12:8-12.
29. Akbarov A, Kontopantelis E, Sperrin M, et al. Primary Care Medication Safety Surveillance with Integrated Primary and Secondary Care Electronic Health Records: A Cross-Sectional Study. *Drug safety* 2015;38(7):671-82. doi: 10.1007/s40264-015-0304-x [published Online First: 2015/06/24]

For peer review only - http://bmjopen.bmj.com/site/about/guidelines.xhtml

30. Amacher AE, Nast I, Zindel B, et al. Experiences of general practitioners, home care nurses, physiotherapists and seniors involved in a multidisciplinary home-based fall prevention programme: a mixed method study. *BMC health services research* 2016;16 doi: 10.1186/s12913-016-1719-5

31. Bell HT, Granas AG, Enmarker I, et al. Nurses' and pharmacists' learning experiences from participating in interprofessional medication reviews for elderly in primary health care - a qualitative study. *BMC family practice* 2017;18(1):30. doi: 10.1186/s12875-017-0598-0 [published Online First: 2017/03/01]

32. Brito AM, Simoes AM, Alcobia A, et al. Optimising patient safety using pharmaceutical intervention in domiciliary hospitalization. *International journal of clinical pharmacy* 2017;39(5):980-84. doi: 10.1007/s11096-017-0512-9 [published Online First: 2017/08/26]

33. Di Monaco M, Vallero F, De Toma E, et al. A single home visit by an occupational therapist reduces the risk of falling after hip fracture in elderly women: A quasi-randomized controlled trial. *Journal of Rehabilitation Medicine* 2008;40(6):446-50. doi: 10.2340/16501977-0206

34. Di Pollina L, Guessous I, Petoud V, et al. Integrated care at home reduces unnecessary hospitalizations of community-dwelling frail older adults: a prospective controlled trial. *BMC geriatrics* 2017;17(1):53-53. doi: 10.1186/s12877-017-0449-9

35. Foged S, Norholm V, Andersen O, et al. Nurses' perspectives on how an e-message system supports cross-sectoral communication in relation to medication administration: A qualitative study. *Journal of clinical nursing* 2018;27(3-4):795-806. doi: 10.1111/jocn.14109 [published Online First: 2017/10/20]

36. Johansen JS, Havnes K, Halvorsen KH, et al. Interdisciplinary collaboration across secondary and primary care to improve medication safety in the elderly (IMMENSE study): study protocol for a randomised controlled trial. *BMJ open* 2018;8(1):e020106. doi: 10.1136/bmjopen-2017-020106 [published Online First: 2018/01/25]

37. Lenander C, Bondesson A, Midlov P, et al. Healthcare system intervention for safer use of medicines in elderly patients in primary care-a qualitative study of the participants' perceptions of self-assessment, peer review, feedback and agreement for change. *BMC family practice* 2015;16(1):117. doi: 10.1186/s12875-015-0334-6 [published Online First: 2015/09/05]

38. Peeters GM, de Vries OJ, Elders PJ, et al. Prevention of fall incidents in patients with a high risk of falling: design of a randomised controlled trial with an economic evaluation of the effect of multidisciplinary transmural care. *BMC geriatrics* 2007;7:15. doi: 10.1186/1471-2318-7-15 [published Online First: 2007/07/04]

39. Toivo T, Airaksinen M, Dimitrow M, et al. Enhanced coordination of care to reduce medication risks in older home care clients in primary care: a randomized controlled trial. *BMC geriatrics* 2019;19(1):332. doi: 10.1186/s12877-019-1353-2 [published Online First: 2019/11/30]

40. van Melle MA, Zwart DLM, Poldervaart JM, et al. Validity and reliability of a medical record review method identifying transitional patient safety incidents in merged primary and secondary care patients' records. *BMJ open* 2018;8(8):e018576. doi: 10.1136/bmjopen-2017-018576 [published Online First: 2018/08/15]

41. van Melle MA, van Stel HKF, Poldervaart JM, et al. Measurement tools and outcome measures used in transitional patient safety; a systematic review. *PloS one* 2018;13(6) doi: 10.1371/journal.pone.0197312

42. Khoo AL, Teng M, Lim BP, et al. A multicenter, multidisciplinary, high-alert medication collaborative to improve patient safety: the Singapore experience. *Joint Commission journal on quality and patient safety* 2013;39(5):205-12. doi: 10.1016/s1553-7250(13)39027-8 [published Online First: 2013/06/12]

43. Sze PC, Cheung WH, Lam PS, et al. The efficacy of a multidisciplinary falls prevention clinic with an extended step-down community program. *Archives of Physical Medicine and*

For peer review only - http://bmjopen.bmj.com/site/about/guidelines.xhtml

Rehabilitation 2008;89(7):1329-34. doi: 10.1016/j.apmr.2007.11.044 [published Online First: 2008/07/01]

44. Mikolaizak AS, Lord SR, Tiedemann A, et al. A multidisciplinary intervention to prevent subsequent falls and health service use following fall-related paramedic care: a randomised controlled trial. *Age and ageing* 2016;46(2):200-07. doi: 10.1093/ageing/afw190
45. Hendriks MR, Bleijlevens MH, van Haastregt JC, et al. Lack of effectiveness of a multidisciplinary fall-prevention program in elderly people at risk: a randomized, controlled trial. *Journal of the American Geriatrics Society* 2008;56(8):1390-7. doi: 10.1111/j.1532-5415.2008.01803.x [published Online First: 2008/07/30]
46. Lenander C, Bondesson Å, Viberg N, et al. Effects of an intervention (SÄKLÄK) on prescription of potentially inappropriate medication in elderly patients. *Family practice* 2017;34(2):213-18. doi: 10.1093/fampra/cmw121
47. Noor H, et al. The development, implementation and evaluation of a transitional care programme to improve outcomes of frail older patients after hospitalisation. *Age and ageing* 2016;45(5):643-51.
48. Murray R, Mckenna H. The Next Steps Towards Integrated Care: The King's Fund, 2020.
49. Jeffs L, Abramovich IA, Hayes C, et al. Implementing an interprofessional patient safety learning initiative: insights from participants, project leads and steering committee members. *BMJ quality & safety* 2013;22(11):923-30. doi: 10.1136/bmjqs-2012-001720 [published Online First: 2013/06/19]
50. OECD. Measuring Patient Safety: Opening the Black Box. France, 2018.
51. Montague J, Crosswaite K, Lamming L, et al. Sustaining the commitment to patient safety huddles: insights from eight acute hospital ward teams. *British Journal of Nursing* 2019;28(20):1316-24.
52. Kelly L, Harlock J, Peters M, et al. Measures for the integration of health and social care services for long-term health conditions: a systematic review of reviews. *BMC health services research* 2020;20(1):358. doi: 10.1186/s12913-020-05206-5
53. Kirst M, Im J, Burns T, et al. What works in implementation of integrated care programs for older adults with complex needs? A realist review. *International Journal for Quality in Health Care* 2017;29(5):612-24.
54. Lalani M, Marshall M. Co-location, an enabler for service integration? Lessons from an evaluation of integrated community care teams in East London. *Health & social care in the community* 2022;30(2):e388-e96. doi: 10.1111/hsc.13211 [published Online First: 2020/11/06]
55. Bussu S, Marshall M. Organisational development to support integrated care in East London: the perspective of clinicians and social workers on the ground. *Journal of health organization and management* 2020;34(5):603-19. doi: 10.1108/jhom-10-2019-0294 [published Online First: 2020/07/19]
56. Crocker H, Kelly L, Harlock J, et al. Measuring the benefits of the integration of health and social care: qualitative interviews with professional stakeholders and patient representatives. *BMC health services research* 2020;20(1):515. doi: 10.1186/s12913-020-05374-4
57. Robert G, Donetto S. Whose data is it anyway? Patient experience and service improvement. 2020;25(3):139-41.
58. Fund TKs. Understanding integration: how to listen to and learn from people and communities The King's Fund, 2021.
59. Smith KM, Valenta AL. Safety I to Safety II: A Paradigm Shift or More Work as Imagined?: Comment on" False Dawns and New Horizons in Patient Safety Research and Practice". *International Journal of Health Policy and Management* 2018;7(7):671.
60. England PH. Falls: Applying All Our Health: Public Health England, 2020.
61. Elliott R, Camacho E, Campbell F, et al. Prevalence and economic burden of medication errors in the NHS in England. 2018

For peer review only

For peer review only - http://bmjopen.bmj.com/site/about/guidelines.xhtml

586 62. Magdelijns FJH, van Avesaath REM, Pijpers E, et al. Health-care-related adverse events leading to admission in older individuals: incidence, predictive factors and consequences. *European journal of public health* 2016;26(5):743-48. doi: 10.1093/eurpub/ckw021

For peer review only

For peer review only - http://bmjopen.bmj.com/site/about/guidelines.xhtml

Figure 1: Flowchart of study selection

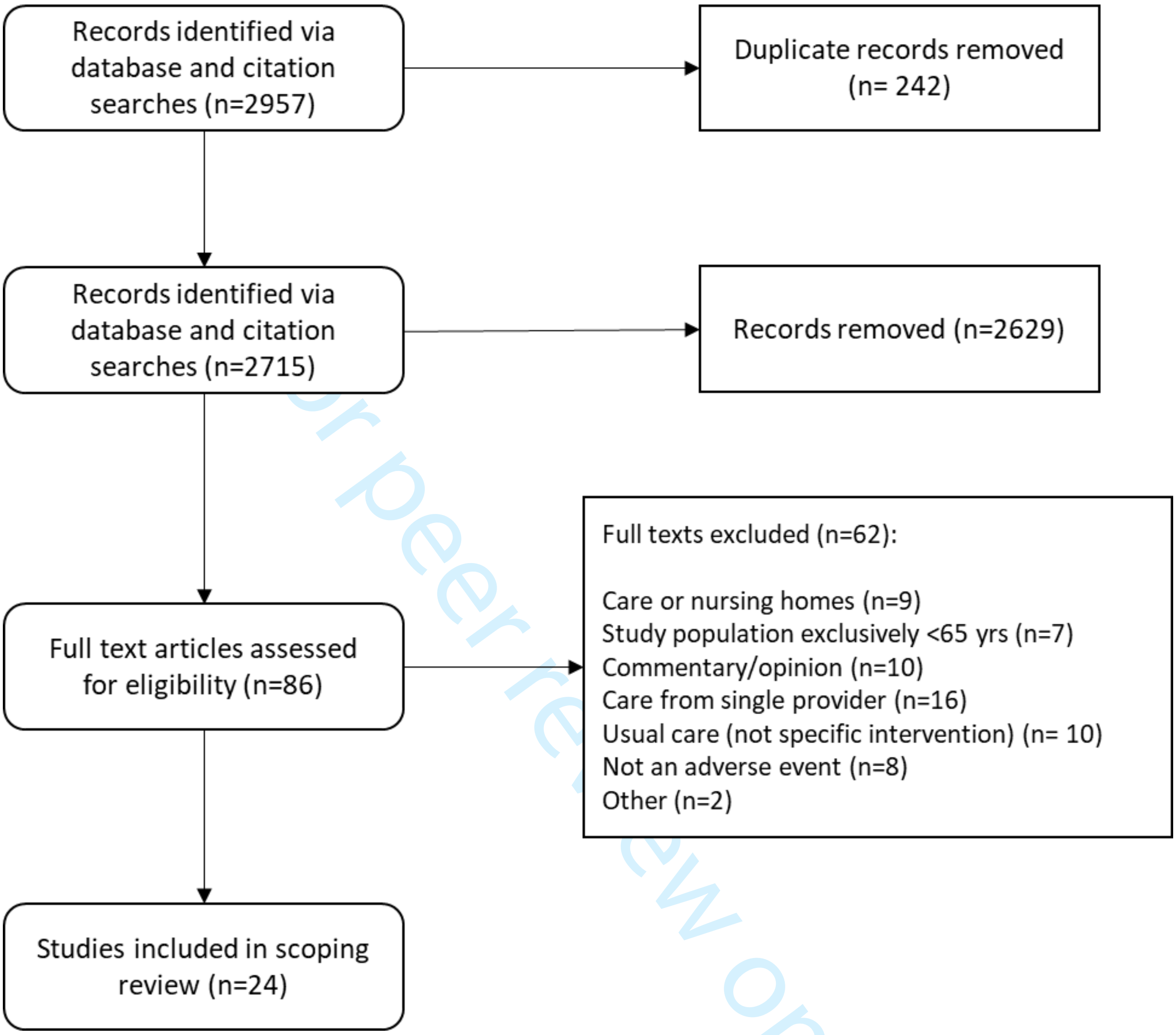

Additional File 1 – search strategy

Search terms (including MeSH) used were:

elderly (elder*) OR older OR older adults OR geriatric OR aged

vulnerable adults

mental health OR disorder

learning disabilities (*disability)

(-term OR long term) conditions

complex care

chronic conditions

multi-morbidity

AND

integrated care

care pathways

multidisciplinary OR multi-disciplinary

multiprofessional OR multi-professional

interprofessional OR inter-professional

transitional (W#) care (transitio*)

care (W#) coordination

AND

patient safety

preventable (w#) harm

risk reduction

adverse events

For peer review only

Supplementary table: Characteristics and description of included studies

Author and year	Country	Aim	Population	Study design	Intervention approaches				Approaches to measuring safety
					**Type of intervention**	**Description of intervention**	**Setting**	**Level**	
**Akbarov 2015 (36)**	England	Investigate the feasibility of wide ranging medication safety surveillance using a linked database	Adults with prescribing hazards	Cross-sectional study	Integrated digital record	Linked records of patients served by one hospital system and primary care centres - prevalence of 22 medication safety indicators, investigation of associations with patient and practice characteristics, and variation between primary care practices	Hospitals and primary care centres	Macro	Outcome measure: Proportion of patients with at least one medication safety hazard
**Amacher 2016 (37)**	Switzerland	Explore the perceived benefits and barriers of an evidence-based, home-based pilot falls prevention programme	Elderly	Mixed methods	MDT approach to care delivery in community (home) or primary care setting	Multi-factorial falls prevention programme (FPP)	Community (home)	Micro	Qualitative outcome: Usefulness of MDT home-based fall prevention programme
**Bell 2017 (38)**	Norway	Investigate nurses' and pharmacists' perceived learning experience after participating in an Integrated Medicines Management Review in primary health care	Elderly	Qualitative study	MDT approach to care delivery in community (home) or primary care setting	Integrated Medicines Management model – centred on inter-professional medication reviews	Community (home based services and nursing homes)	Macro	Qualitative outcome: Extent of professional learning experiences to support medicines management

For peer review only

**Brito 2017 (39)**	Portugal	Managing medication, in a patient-centred process and multidisciplinary context, by gathering information about the drug therapy of patients before admission, during hospitalization and after hospital discharge	Elderly	Observation study	Care pathway	Pharmacist working within domiciliary hospitalisation unit (DHU) alongside a multi-disciplinary team – medication reconciliation and review	Community (home) – domiciliary hospitalisation	Micro	Qualitative outcome: Perceived quality of DHU delivery and its impact on drug safety
**Di Monaco 2008 (40)**	Italy	Assessment of the effectiveness of a follow up home visit by an occupational therapist after a fall. MDT prevention intervention to reduce falls risk after hip fracture in elderly women	60 years or older	Quasi-RCT	Care pathway	MDT approach to prevent falls; exercise, advice and training on use of assistive devices, and activities of daily living	Hospital and community (home)	Micro	Outcome measure: Occurrence of falls in six months post-discharge from hospital
**Di Pollina 2017 (41)**	Switzerland	Testing the efficacy of providing integrated care at home to reduce unnecessary hospitalisations, emergency room visits, institutionalisation, and mortality in community	Frail and dependent people over 60 years	Prospective RCT	MDT approach to care delivery in community (home) or primary care setting	Integrated in-home geriatric assessments, long-term coordinated follow-up, and availability of a round-the-clock geriatric call service	Community (home)	Micro	Outcome measure: Falls over 3 years as a secondary outcome (stated as reason for hospitalisation)

For peer review only

		dwelling frail older adults							
**Eckstrom 2016 (30)**	US	Establishing changes in team behaviours after the intervention communicate and enhance adherence to Geriatrics Society Falls Prevention Guidelines by inter-professional teams in practice) and highlight lessons learned during the practice change process	Elderly patients	Mixed methods	Educational intervention	Falls prevention strategy training workshops to clinical inter-professional teams – content based on guidelines developed by American Geriatric Society and British Geriatric Society	Hospitals and community or primary care clinics	Macro	Process measure: Adherence to falls prevention strategies<br><br>Outcome measure: Rate of falls, emergency department visits for falls, and hospitalisations for falls over a three month period
**Foged 2018 (42)**	Denmark	Describe nurse perspectives on how an e-message system supports communication on medication administration between hospital and home care nurses	Elderly	Qualitative	Integrated digital record	E-message system - admission report, care plan, discharge report, discharge notification and a message platform to bolster communication between hospital nurses and nurses providing home-based care	Hospital and community (home)	Macro	Qualitative outcome: The ability of an E-message system to support information exchange to assure medication safety
**Hendriks 2008 (52)**	Netherlands	Assess whether a MDT fall-prevention program was more effective than standard care in preventing new falls and functional decline in elderly community-dwelling people	Adults, 65 years and over	RCT	Care pathway	MDT in hospital providing a falls risk assessment, with referral to GP depending on risk and occupational therapy visits for a short period at home	Hospital and community (home)	Micro	Outcome, Primary measure: number of falls experienced in 12 month follow up period

For peer review only

		who attended an emergency department after a fall							
**Hiem 2016 (54)**	Netherlands	Decrease the risk of functional decline and loss of independence after hospitalisation in frail older patients	Elderly patients	Action research	Care pathway	Various interventions across primary and acute care to support frail patients. Interventions address (i) improved risk management; (ii) delivery of integrated, function-oriented care; (iii) specific geriatric interventions; and (iv) optimisation of transfers	Primary care, nursing home, rehabilitation centres and hospitals	Macro	Outcome: Incidence of adverse outcomes including falls in the last six months
**Johansen 2018 (43)**	Norway	Explore the effects of the intervention on healthcare use, health-related quality of life and medication appropriateness in elderly patients	70 yrs and over	Protocol RCT	Care pathway	Integrated medicines management model adapted to improve both medication safety in hospital and communication across primary and secondary care. Medication reconciliation (admission), medication review (during hospital stay), patient counselling (medicines use), medication list with explanations in discharge summary and post-discharge phone calls to primary care	Secondary and primary care	Meso	Secondary process measure: medication appropriateness, medication changes,<br><br>Outcome measure: hip fracture rate, medication-related readmissions

For peer review only

**Khoo 2013 (49)**	Singapore	Reduce adverse drug events resulting from high-alert medication	Adults on 'high risk of adverse events' medications	QI case study	Care pathway	Multicentre multidisciplinary collaborative focused on improving safe use of high-alert medication. QI approach with tracking of improvement conducted with an adapted IHI Trigger Tool method	Primary and acute care institutions	Macro	Outcome measure: Preventable or potentially preventable adverse drug events
**Laugaland 2012 (16)**	Global	Identify and evaluate the effects of the interventions in terms of effectiveness and efficiency of care processes in the context of effects of discharge interventions on patient safety	Patients 65 years and over	Review	Care pathway	Several studies that employed MDT approaches to transitional care to improve patient safety	Hospital, community and primary care	Meso	Process and outcome measures associated with adverse events
**Lenander 2015 (53)**	Sweden	Evaluate participants' perceptions of the SÄKLÄK project, which aims to enhance medication safety, especially for elderly patients, in primary care	Elderly	Qualitative study	MDT approach to care delivery in community (home) or primary care setting	SÄKLÄK intervention model involves a multidisciplinary group with self-assessment, peer review, feedback and agreement for change	Primary care	Meso	Qualitative outcome: Usefulness of self-assessment tool in identifying areas of improvement for medication safety

For peer review only

**Mckenzie 2017 (31)**	US	Describe an inter-professional team-based training approach to falls prevention for older adults and the related educational outcomes	Older adults	Service evaluation	Educational intervention	Project that involves an inter-professional teaching team to support practice teams to reduce falls in older adults via implementation of evidence-based practice guidelines	Ambulatory, long term care, hospital, and home health practice	Macro	Process measure: Associated with educational outcomes resulting from falls training workshops; knowledge, learner confidence, intention for collaborative practice change and change to practice (falls screening rates and falls risk assessments)
**McQuaid-Bascon 2018 (35)**	Canada	An overview of a recently integrated health care system's experience in evolving their multi-component inter –professional approach to falls prevention	Older patients susceptible to falls	Service evaluation	Care pathway	Establishment of multidisciplinary falls committee to provide leadership, direction and coordination in the inter-professional advancement of quality improvement, evidence-informed practices and research associated with falls prevention and injury minimisation	Hospital	Meso	Process measure: i) sustain falls risk assessment and falls communication bundle across all areas; ii) monitor and report falls assessment rates and bundle implementation on inpatient areas; and iii) explore patient video monitoring and soft cell flooring<br><br>Outcome measure: improve falls with injury rates by 25%;
**Mikolaizak 2016 (51)**	Australia	Determine whether an approach involving timely assessment and tailored interventions, resulted in a meaningful reduction in subsequent falls, fall-related injuries and associated emergency health service use in older people who were	Older adults 65 years and over	RCT	MDT approach to care delivery in community (home) or primary care setting	Targeted identified risk factors and used existing services to implement physiotherapy, occupational therapy, geriatric assessment, optometry and medication management interventions to prevent falls	Community (home)	Micro	Outcome measure: Rate of falls and fall-related injuries during the 12-month follow-up period

		not transported to hospital following a fall							
**Peeters 2007 (45)**	Netherlands	Reduce the fall risk in older persons with a high risk of falling. The intervention consists of a systematic assessment of the putative causes of falling and subsequent targeted individualised preventive measures	Older adults >65	RCT	Care pathway	Assessment by geriatric outpatient clinic for falls risk factors in collaboration with a MDT that provides multifactorial treatment that may include physiotherapy, ophthalmic assessment and medicines management	Community (home)	Micro	Process measure: proportion assessed for; risk of recurrent falling, fall history, medical history, medication use, independence in activities of daily living Outcome measure: Time to first and second fall
**Robbins 2013 (32)**	US	To describe the process followed and outcomes achieved from participating in a pharmacy clinical services and patient safety collaborative and integrating pharmacy services into a high-risk, ambulatory population	Adults	QI case study	Care pathway	Patient Safety and Clinical Pharmacy Services Collaborative change package involving a MDT	Health centre	Macro	Outcome measure: Percentage of adverse drug events detected in the population of focus that received clinical pharmacy services during the month of follow up
**Sze 2008 (50)**	Hong Kong	To investigate the efficacy of a falls prevention clinic and a community step-down programme in	Elderly patients	Prospective cohort	MDT approach to care delivery in community (home) or	12 week falls clinic provided by a MDT - including fall evaluation, balance training, home hazard	Community (home)	Micro	Primary outcome measure: Number of falls per person per year<br><br>Secondary outcome measure: Fall-related injuries

For peer review only

		reducing the number of falls among community-dwelling elderly at high risk of a fall			primary care setting	management program, and medical referrals. Followed by a community step-down (nine months) program, including falls prevention education, a weekly exercise class, and two home visitations			
**Toivo 2019 (46)**	Finland	To assess the impact of a care coordination intervention on medication risks identified in drug regimens of older home care clients over a one-year period	Elderly patients	Cluster RCT	MDT approach to care delivery in community (home) or primary care setting	Coordinated medication risk management (CoMM) model which involves a multi-stage intervention; triage, medication risk screening and reviews, actions plans delivered by a MDT	Community (home)	Micro	Process measure: Several including use of harmful medications<br><br>Outcome: clinically significant drug interactions
**Van Melle 2018 (47)**	Netherlands	To pilot a review of medical records to identify transitional safety incidents (TSIs) for use in a large intervention study and assess its reliability and validity.	Adults under cardiology and gastro service care	Retrospective medical record review study	Integrated digital record	Transitional medical record system: establish inter-rater reliability of a measurement tool designed to assess transitional safety incidents	Hospital and primary care	Macro	Outcome measures: Transitional safety incidents which include but not restricted to adverse drug events leading to unplanned hospital admission
**Wallace 2018 (33)**	US	To evaluate the effects of a multidisciplinary hip fracture care pathway on the outcomes of the elderly patients	Elderly	Retrospective analysis	Care pathway	Multidisciplinary care pathway for reduce hip fractures in the elderly	Hospital (Trauma centre)	Micro	Outcome measures: Several – includes mechanism of injury (such as falls), hip fracture characteristics and hospital length of stay

For peer review only

**Willis 2011 (34)**	US	To determine if trained undergraduate students, in conjunction with pharmacists, could provide in home medication reviews, and demonstrate benefit to the health and welfare of a senior population affiliated Patient-Centred Medical Home	Patients aged 65 years and over	Service evaluation	MDT approach to care delivery in community (home) or primary care setting	Patient-Centred Medical Home model - In-home medication reviews, assessing a home for fall risk, and performing blood pressures	Community (home)	Meso	Process measures: Proportion of patients prescribed falls risk medication and medicines that have a high potential for major drug interaction. Proportion of patients that have had a medication change made to their clinical medication list

For peer review only

Preferred Reporting Items for Systematic reviews and Meta-Analyses extension for Scoping Reviews (PRISMA-ScR) Checklist

SECTION	ITEM	PRISMA-ScR CHECKLIST ITEM	REPORTED ON PAGE #
**TITLE**			
Title	1	Identify the report as a scoping review.	
**ABSTRACT**			
Structured summary	2	Provide a structured summary that includes (as applicable): background, objectives, eligibility criteria, sources of evidence, charting methods, results, and conclusions that relate to the review questions and objectives.	
**INTRODUCTION**			
Rationale	3	Describe the rationale for the review in the context of what is already known. Explain why the review questions/objectives lend themselves to a scoping review approach.	
Objectives	4	Provide an explicit statement of the questions and objectives being addressed with reference to their key elements (e.g., population or participants, concepts, and context) or other relevant key elements used to conceptualize the review questions and/or objectives.	
**METHODS**			
Protocol and registration	5	Indicate whether a review protocol exists; state if and where it can be accessed (e.g., a Web address); and if available, provide registration information, including the registration number.	
Eligibility criteria	6	Specify characteristics of the sources of evidence used as eligibility criteria (e.g., years considered, language, and publication status), and provide a rationale.	
Information sources*	7	Describe all information sources in the search (e.g., databases with dates of coverage and contact with authors to identify additional sources), as well as the date the most recent search was executed.	
Search	8	Present the full electronic search strategy for at least 1 database, including any limits used, such that it could be repeated.	
Selection of sources of evidence†	9	State the process for selecting sources of evidence (i.e., screening and eligibility) included in the scoping review.	
Data charting process‡	10	Describe the methods of charting data from the included sources of evidence (e.g., calibrated forms or forms that have been tested by the team before their use, and whether data charting was done independently or in duplicate) and any processes for obtaining and confirming data from investigators.	
Data items	11	List and define all variables for which data were sought and any assumptions and simplifications made.	
Critical appraisal of individual sources of evidence§	12	If done, provide a rationale for conducting a critical appraisal of included sources of evidence; describe the methods used and how this information was used in any data synthesis (if appropriate).	
Synthesis of results	13	Describe the methods of handling and summarizing the data that were charted.	

For peer review only - http://bmjopen.bmj.com/site/about/guidelines.xhtml

SECTION	ITEM	PRISMA-ScR CHECKLIST ITEM	REPORTED ON PAGE #
**RESULTS**			
Selection of sources of evidence	14	Give numbers of sources of evidence screened, assessed for eligibility, and included in the review, with reasons for exclusions at each stage, ideally using a flow diagram.	
Characteristics of sources of evidence	15	For each source of evidence, present characteristics for which data were charted and provide the citations.	
Critical appraisal within sources of evidence	16	If done, present data on critical appraisal of included sources of evidence (see item 12).	
Results of individual sources of evidence	17	For each included source of evidence, present the relevant data that were charted that relate to the review questions and objectives.	
Synthesis of results	18	Summarize and/or present the charting results as they relate to the review questions and objectives.	
**DISCUSSION**			
Summary of evidence	19	Summarize the main results (including an overview of concepts, themes, and types of evidence available), link to the review questions and objectives, and consider the relevance to key groups.	
Limitations	20	Discuss the limitations of the scoping review process.	
Conclusions	21	Provide a general interpretation of the results with respect to the review questions and objectives, as well as potential implications and/or next steps.	
**FUNDING**			
Funding	22	Describe sources of funding for the included sources of evidence, as well as sources of funding for the scoping review. Describe the role of the funders of the scoping review.	

JBI = Joanna Briggs Institute; PRISMA-ScR = Preferred Reporting Items for Systematic reviews and Meta-Analyses extension for Scoping Reviews.
* Where *sources of evidence* (see second footnote) are compiled from, such as bibliographic databases, social media platforms, and Web sites.
† A more inclusive/heterogeneous term used to account for the different types of evidence or data sources (e.g., quantitative and/or qualitative research, expert opinion, and policy documents) that may be eligible in a scoping review as opposed to only studies. This is not to be confused with *information sources* (see first footnote).
‡ The frameworks by Arksey and O'Malley (6) and Levac and colleagues (7) and the JBI guidance (4, 5) refer to the process of data extraction in a scoping review as data charting.
§ The process of systematically examining research evidence to assess its validity, results, and relevance before using it to inform a decision. This term is used for items 12 and 19 instead of "risk of bias" (which is more applicable to systematic reviews of interventions) to include and acknowledge the various sources of evidence that may be used in a scoping review (e.g., quantitative and/or qualitative research, expert opinion, and policy document).

From: Tricco AC, Lillie E, Zarin W, O'Brien KK, Colquhoun H, Levac D, et al. PRISMA Extension for Scoping Reviews (PRISMAScR): Checklist and Explanation. Ann Intern Med. 2018;169:467–473. doi: 10.7326/M18-0850.

For peer review only - http://bmjopen.bmj.com/site/about/guidelines.xhtml

BMJ Open

Approaches to improving patient safety in integrated care: a scoping review

Journal:	*BMJ Open*
Manuscript ID	bmjopen-2022-067441.R1
Article Type:	Original research
Date Submitted by the Author:	17-Jan-2023
Complete List of Authors:	Lalani, Mirza ; London School of Hygiene and Tropical Medicine Department of Health Services Research and Policy<br>Wytrykowski, Sarah; Medical Research Network<br>Hogan, Helen; London School of Hygiene & Tropical Medicine, Health Services Research and Policy
**Primary Subject Heading**:	Health services research
Secondary Subject Heading:	Health services research
Keywords:	Organisation of health services < HEALTH SERVICES ADMINISTRATION & MANAGEMENT, Quality in health care < HEALTH SERVICES ADMINISTRATION & MANAGEMENT, Health & safety < HEALTH SERVICES ADMINISTRATION & MANAGEMENT

SCHOLARONE™
Manuscripts

I, the Submitting Author has the right to grant and does grant on behalf of all authors of the Work (as defined in the below author licence), an exclusive licence and/or a non-exclusive licence for contributions from authors who are: i) UK Crown employees; ii) where BMJ has agreed a CC-BY licence shall apply, and/or iii) in accordance with the terms applicable for US Federal Government officers or employees acting as part of their official duties; on a worldwide, perpetual, irrevocable, royalty-free basis to BMJ Publishing Group Ltd ("BMJ") its licensees and where the relevant Journal is co-owned by BMJ to the co-owners of the Journal, to publish the Work in this journal and any other BMJ products and to exploit all rights, as set out in our licence.

The Submitting Author accepts and understands that any supply made under these terms is made by BMJ to the Submitting Author unless you are acting as an employee on behalf of your employer or a postgraduate student of an affiliated institution which is paying any applicable article publishing charge ("APC") for Open Access articles. Where the Submitting Author wishes to make the Work available on an Open Access basis (and intends to pay the relevant APC), the terms of reuse of such Open Access shall be governed by a Creative Commons licence – details of these licences and which Creative Commons licence will apply to this Work are set out in our licence referred to above.

Other than as permitted in any relevant BMJ Author's Self Archiving Policies, I confirm this Work has not been accepted for publication elsewhere, is not being considered for publication elsewhere and does not duplicate material already published. I confirm all authors consent to publication of this Work and authorise the granting of this licence.

For peer review only

Approaches to improving patient safety in integrated care: a scoping review

Mirza Lalani,[1*] Sarah Wytrykowski,[2] Helen Hogan[1]

[1] Department of Health Services Research and Policy, London School of Hygiene and Tropical Medicine

[2] Medical Research Network

*Corresponding author: Mirza Lalani, mirza.lalani@lshtm.ac.uk

Abstract

Objectives: This scoping review aimed to establish the approaches employed to improving patient safety in integrated care for community dwelling adults with long term conditions

Design: Scoping Review

Setting: All care settings

Search strategy: Systematic searches of seven academic and grey literature databases for studies published between 2000-2021. At the full text review stage both the first and second reviewer (SW) independently assessed full texts against the eligibility criteria and any discrepancies were discussed

Results: Overall, 24 studies were included in the review. Two key priorities for safety across care boundaries for adults with long term conditions were falls and medication safety. Approaches for these priorities were implemented at different levels of an integrated care system.At the micro level, approaches involved patient-centred care primarily in the home setting provided by multi-disciplinary teams. At the meso-level, the focus was on planning and designing approaches at the managerial/organisational level to deliver multi-disciplinary care. At the macro level, system-wide approaches included integrated care records, training and education and the development of care pathways involving multiple organisations. Across the included studies evaluation of these approaches was undertaken using a wide range of process and outcome measures to capture patient harm and contributory factors associated with falls and medication safety

For peer review only

Conclusions:. For integrated care initiatives to fulfil their promise of improving care for adults with long term conditions, approaches to improve patient safety need to be instituted across the system, at all levels to support the structural and relational aspects of integrated care as well as specific risk-related safety improvements.

Keywords: Patient safety, integrated care, falls, medication safety

Word count: 4736

Strengths and limitations:

- Highlighting how key components of integrated care can also improve patient safety across care boundaries particularly in reducing falls and medication safety management
- Another strength of the study was the rigorous approach to scoping and selecting the literature including the comprehensive searching of several academic and grey literature databases and sources
- Nonetheless, the identification of relevant studies was possibly hindered by the lack of consistency and interchangeability of the terms ‘integrated care and patient safety’ and their use in the literature.
- While the review was able to identify the different types of patient safety approaches (scope) it was more challenging to describe their scale across care boundaries.

For peer review only - http://bmjopen.bmj.com/site/about/guidelines.xhtml

Introduction

Health and social care systems globally are facing the challenges of an ageing population with complex co-morbidities that require multi-faceted care provision to better manage their health and care needs. [1] Care provision for adults (particularly older and frail) with long term conditions can be fragmented, impacting the quality of care, with a greater potential for adverse outcomes. [2] The integration of health and social care systems and services is seen as integral to tackling these challenges. [3] The term 'integration' broadly represents a 'joining up' of traditional silos of care across (horizontal) and within (vertical) systems, organisations and services. Integrated care transcends longstanding professional, organisational and regulatory boundaries to provide more coordinated, collaborative and person-centred care. [4-6] Many high-income countries have taken steps to deliver integrated care through integrated care systems which involve the collaboration of sectors and care providers, bringing together primary and secondary healthcare, social care, community and mental health services and voluntary organisations. [7]

The World Health Organisation (WHO) defines patient safety as 'the absence of preventable harm to a patient during the process of health care and reduction of risk of unnecessary harm associated with health care to an acceptable minimum.'[8] Risks to patient safety can be mitigated within integrated care systems by strengthening some of the key structural and relational aspects of service provision such as communication, partnership working, care coordination and person-centred care as well as intervening to reduce specific harms. [6]

A handful of conceptual models of integrated care have been described in the literature. One such model suggests integrated care has a supporting role at all levels of the system, micro (services, meso (organisations) and macro (systems). [9] Recent approaches to better management, assurance and improvement of patient safety have advocated for developing safety governance through a whole systems approach addressing safety issues at the all levels of a care system. [10] An overview of

For peer review only - http://bmjopen.bmj.com/site/about/guidelines.xhtml

the role of integrated care and patient safety at the different levels of a system is presented in table 1 below.

Table 1: The role of integrated care and patient safety at different levels of the care system. Overview of conceptual models developed by Valentijn et al [9] and Auraaen et al.[10]

Level of system	Integrated care	Patient safety
Micro level (clinical or service)	Clinical integration in practice is a disease focussed approach to coordinating care. There has been a gradual transition to a more person-centred approach in the provision of services. e.g. multi-disciplinary team (MDT) meetings focussed on a defined group of patients.	Approaches spanning organisations but managed within a clinical microsystem. Includes fostering closer working between patients/carers, administrative staff and practitioners to minimise risk e.g. surgical safety checklist.
Meso level (professional or organisation)	Professional or organisational integration involves inter-professional, partnership working (e.g. nurses and social workers) representing organisations from different sectors (e.g. acute and social care) with a collective responsibility for improving the health of the populations they serve. e.g. integrated community care teams providing care to primarily community dwelling adults with complex care needs	Approaches aimed at particular clinical area or patient type but implemented across an entire organisation. e.g. clinical incident reporting
Macro level (systems)	Systems come together to develop key governance, managerial and administrative structures required for integration with the aim of combining vertical and horizontal integration to create partnerships that provide a continuum of care across care boundaries. e.g. integrated care record	Initiatives implemented across an entire system that requires legislative or high-level policy levers. Benefit gained from public engagement. e.g. pay for performance schemes

Integrated care can contribute to improving the safety of care for people with long term conditions at different levels, particularly by addressing the risks posed by multiple referrals, handovers and discharges that arise as the responsibility for care crosses care boundaries and involves different practitioners, teams, organisations and systems. [11] Even so, integrated care initiatives themselves pose a risk to patient safety as care shifts away from hospitals and closer to home. Adverse events in the community setting cause between 8-12% of hospital admissions, around a half of which are thought to be preventable. [12 13] Harm in this setting may result from medication mismanagement, poor infection control, failure to ensure safe mobilisation or a lack of prevention of and care for pressure sores/ulcers. [14] Studies have also highlighted that the 'spaces' between care settings are

For peer review only

For peer review only - http://bmjopen.bmj.com/site/about/guidelines.xhtml

prone to problems of communication and coordination which affect the safety of care as patients transition across care boundaries and receive care from different professionals. [15,16]

Initial scoping of the literature on how safety is improved for adults with long term conditions in integrated care revealed a limited evidence based on the scale and scope of safety approaches implemented in integrated care systems. As the relevant literature to address this issue was likely to be extensive, complex and potentially heterogeneous, a scoping review was selected over a systematic review. [17] This scoping review aims to address this gap by establishing the approaches employed to improve patient safety in integrated care for community dwelling adults with long term conditions and how these are evaluated. Such assessments will gauge current key priorities for safety within integrated care and the types of patients safety measures that may be useful for ongoing monitoring of safety at different levels of a system.

Methods

This scoping review was conducted in accordance with the PRISMA extension for scoping reviews (PRISMA-ScR) guidance.[18] A protocol was developed but not published. We used the Arksey and O'Malley's framework in undertaking this scoping review; (i) identifying the research question; (ii) identifying relevant studies; (iii) selecting studies; (iv) charting the data; (v) collating, summarising and reporting the results.

Stage 1: Identifying the research questions

The scoping review explored the breadth of literature to answer the research question, 'What type of integrated care approaches exist at different system levels to improve patient safety for community-dwelling adults with long term conditions and how have they been assessed?'

For peer review only - http://bmjopen.bmj.com/site/about/guidelines.xhtml

Stage 2: Identifying relevant studies

Free text search terms were developed based on our research question. The search term strategy (supplementary file 1) was broadly based on three blocks of terms; 1) population, 2) approach (associated with integrated care), and c) outcome (related to patient safety).

A comprehensive search strategy was employed to include both the peer-reviewed and grey literature. Seven electronic databases were searched including MEDLINE, Social Policy and Practice, Web of Science, CINAHL, Google Scholar, Proquest and OpenGrey. These were supplemented by forward and ancestry citation searches using reference lists of several relevant studies.

Stage 3: Selecting studies

Study selection was based on the inclusion and exclusion criteria listed in table 2.

Table 2: Overview of exclusion and inclusion criteria

**Inclusion criteria**	
Organisational setting	All types i.e. acute, primary, community, mental health and social care
Population	Adults over 18 with long term conditions
Approach	Involved at least two different types of organisations e.g. hospital and community i.e the initiative under assessment spanned different sectors
Outcome	Associated with patient safety as defined by the WHO
Study design	Primary and/or secondary data (research studies, quality improvement project reports, service evaluations and reviews)
Time interval	2000-2021 - We selected 2000 as a start date because it coincides with a proliferation in safety-related research stimulated by the publication of the seminal report; To Err is Human: Building a Safer Health System. (21)
	Studies published in English only
**Exclusion criteria**	
Organisational setting	Transitions of care within the same sector e.g. hospital only<br>Studies in which it is challenging to discern whether staff in an MDT were employed by different providers and therefore were part of an integrated care team
Type of study	We also excluded commentaries and editorials.

The first reviewer (ML) undertook initial searches and based on information in the title and abstract selected those meeting criteria for full text review. At this stage, any studies that the first reviewer was uncertain about in terms of meeting eligibility criteria were checked by the second reviewer. At

For peer review only

the full text review stage both the first and second reviewer (SW) independently assessed full texts against the eligibility criteria and any discrepancies were discussed.

Stage 4: Charting the data

The first reviewer extracted and tabulated the data from the included studies in Microsoft Excel. Details of the studies extracted included the overall aim, population, study design, level of system, integrated care approach, setting and approach to measuring safety. The second reviewer reviewed and verified the data extraction table. Descriptions of the levels of the system i.e. micro, meso and macro and the approach to measuring safety (outcome, process and qualitative) [19] are outlined in box 1. The nomenclature for different levels of a system (micro, meso and macro) can be ascribed to both integrated care and patient safety system governance. [10 20] The Donebedian framework for measuring quality in health care applies to identifying measures for safety i.e. outcome and process, in the case of this review. [21] We have also added a further measure, 'qualitative' to capture perceptions of study participants regarding service improvements associated with patient safety through implementation of approaches.

For peer review only - http://bmjopen.bmj.com/site/about/guidelines.xhtml

Box 1: Description of the different levels of a care system

Levels of care system:

Micro-level: Typically comprises an approach at the patient level where multiple professionals from different types of organisations provide care commonly in the home setting but sometimes in primary care alone or in primary and secondary care if linked by a common pathway.**Meso level:** Approaches at organisation level that involve multi-professionals in planning or designing local structures and services that span two or more different types of organisations.

Macro level: System wide approaches that may involve several providers with a typical focus on strategy, infrastructure development and other wide-ranging cultural and educational initiatives. Approacehs implemented over a wider a geography within a country e.g. region, county, state etc.

Approaches to measuring safety:

Outcome measures: Indicate the impact of a health care service or approach on the patient's health status.

Process measures: Practices delivered in order to achieve the desired outcome of an approach.

Qualitative outcomes: Perspectives of study participants on the implementation and/or impact of an approach (primarily from qualitative studies only).

Quality assessment

Due to the heterogeneity of the studies in terms of their methodological design, settings in which an approach was implemented as well the design and scope of the approach itself, and in line with similar scoping reviews, formal quality assessment was not undertaken. [22 23]

Patient and Public Involvement

No patients or members of the public were directly involved in this work. However, this study is part of a wider programme of research for which there is a research advisory group whose membership includes patients/public representatives. In the conceptualisation phase of the study, the research advisory group provided advice and guidance on the study's aims and purpose.

For peer review only - http://bmjopen.bmj.com/site/about/guidelines.xhtml

Results

Stage five: collation, summarising and reporting

The original searches yielded 2957 records. After removing duplicate records and screening of titles and abstracts, 86 were allocated for full text review. After application of eligibility criteria, 24 studies were included in the review. The flow of studies from initial identification to final inclusion are presented in figure 1.

Figure 1: Flowchart of study selection

General characteristics of included studies

All included studies were published between 2007 and 2019. We identified one review [14] and 23 empirical studies. A breakdown of the study characteristics is provided in the supplementary table. In terms of geographical distribution, empirical studies were undertaken in North America [24-29], Europe [30-42] Asia [43 44] and Australia [45]. All studies involved adult participants described as elderly, older adults or adults over the age of 65 in the main, although four studies also included other adults aged over 18. In all studies, the population in receipt of an approach were reported to have long-term, chronic physical or mental health conditions. However, in a few of these studies, a health condition(s) was not specified and instead participants subject to the approach were described as frail or vulnerable. Empirical studies comprised the majority of study types and included randomised control trials (RCT), [34 35 37 39 40 45 46] qualitative studies, [32 33 36 47] mixed methods studies, [24 31] retrospective analysis of medical records, [27 41] a cohort study, [44] a cross-sectional study, [30] an observational study,[33] a systematic review [14] and action research. [48] Service evaluations [25 28 29] and quality improvement (QI) studies [26 43] were also among the list of included studies.

Types of risks addressed

For peer review only

Of the 24 included studies, 11 involved approaches designed to reduce falls risk, [24 25 29 31 34 35 39 44-46 48] and ten studies were broadly associated with assuring medication safety through reconciliation and review, surveillance and monitoring [26 30 32 33 36 37 40 41 43 47]. Three studies undertook a series of approaches to reduce adverse events more broadly such as identifying medications that increase the risk of a fall in older people and an assessment of an approach designed to reduce the rate of hip fracture. [27 28] The systematic review included studies that involved approaches to reduce adverse events including adverse drug reactions. [14]

Integrated care approaches

Setting

The studies included a range of settings. A total of 13 studies described approaches implemented in a single setting; the home setting [28 31 32 35 39 40 44 45] and the remainder in hospitals, [27 29 33] community health centres, [26] and primary care. [47] In the remaining studies, a care pathway was established either across several settings [14 25 43 48] or just two settings; between the hospital and home setting [34 36 46] or hospital and primary care.[24 30 37 41] Studies based in a single setting included approaches that involved professionals from different sectors across social, acute, primary and community working together (often in the same team) to provide care.

Level of care system

Various approaches were implemented at different levels of the care system; micro, meso and macro. Ten studies were at the micro level, [27 31 33-35 39 40 44-46] five at the meso level [14 28 29 37 47] and nine at the macro level [24-26 30 32 36 41 43 48]. Broadly, these approaches can be categorised into four types; i) MDT approach to care delivery in community (home) or primary care setting, ii) care pathway, iii) educational initiative and iv) integrated digital record (accessible to different care providers).

Micro level

For peer review only - http://bmjopen.bmj.com/site/about/guidelines.xhtml

For peer review only

196 At the micro level, approaches were characterised by the delivery of person-centred coordinated care for adults with long term conditions involving the home (community) setting or primary care setting alone or as part of a care pathway with secondary care settings. Care was provided by a range of health and care professionals working together. Three studies outlined multidisciplinary approaches to prevent and reduce falls, primarily in the home setting such as exercise therapy, home based assessments for hazards with accompanying modifications, vision assessment, home medication review and a 24 hour call service staffed by a home intervention team.[31 35 45]

203 A further five studies described approaches implemented across different parts of a care pathway to reduce falls, although *Wallace et al* focussed on the development of a multi-disciplinary care pathway following a hip fracture. [27 34 39 44 46] Approaches comprised multi-disciplinary outpatient clinics (often located in hospitals) where patients were assessed and monitored, with further follow-up care provided by community services staff in the home setting. [34 39 44 46] Follow up care included assistance with daily living, medication review, balance and cognition, [34 44 46] as well as promotion of patient/user independence through home hazard (home environment risk) assessments, [39 44 46] exercise to improve mobility and strength, [34 39 44] and patient education on use of assisted devices to support individuals to navigate their home environment. [34]

212 Three studies involved medicines reviews to reduce the risk of adverse drug events (ADE) including the reduction of falls. [28 40 47]

214 <u>Meso-level</u>

215 At the meso-level approaches were mainly focused on multi-professional management to plan and design services across at least two different types of organisation, primarily hospitals and community care. Approaches tackled areas with overarching safety risks such as poor communication or dispersed leadership. All five studies used different approaches.

For peer review only - http://bmjopen.bmj.com/site/about/guidelines.xhtml

Three studies aimed to improve medication safety for older people. One study employed an Integrated Medicines Management (IMM) model (spanning hospitals and primary care) which comprised medicines reconciliation and review, patient counselling on medicines usage during hospital stay and greater provision of information and communication with primary care at discharge and post-discharge. [37] Another study described an approach in primary care involving managers, pharmacists and nurses who engaged in self-assessment, peer review, feedback and agreement for change to improve medicines safety management. [47] *Willis et al* described an MDT approach involving medication reviews, home fall risk assessments and blood pressure monitoring. [28]

A further study, outlined a multidisciplinary falls committee (within a newly created ICS) whose role was to provide leadership, direction and coordination to frontline staff promoting new evidence based practices and quality improvement support to prevent falls. [29] The systematic review described approaches such as education and training, organisational/culture interventions (e.g. discharge protocol and planning, medication reconciliation etc.), or patient/family orientated approaches concerning awareness and empowerment for patients. [14]

Macro level

At the macro level, system-wide approaches centred on managing medication safety and reducing falls risk such as key infrastructure developments - integrated care records, training and education initiatives spanning several organisations within a system and creating care pathways involving multiple organisations. Approaches were generally implemented across a wider geography e.g. county, state or region.

Three studies described digital approaches to medication safety monitoring such as a digital database for patients transitioning from secondary to primary care with indicators to alert practitioners to potential ADE [30] and a digital measurement tool designed to assess transitional safety incidents affecting older people including ADE. [41] A further study described how an e-message

For peer review only - http://bmjopen.bmj.com/site/about/guidelines.xhtml

For peer review only

system for nurses in hospital and community settings could support information exchange, minimising ADE. [36]

Two studies described educational approaches comprising training of inter-professional teams (including social workers) from a range of settings on developing strategies for preventing falls in accordance with national evidence-based guidelines. [24 25]

The remaining studies described multi-component models such as quality improvement collaborative approaches, or other models comprising multi-disciplinary working, care coordination and strategies to strengthen leadership to promote patient safety and reduce hospital admission or readmission. The Quality improvement collaborative aimed to reduce the risk of ADE and involved MDTs with professionals from primary and acute care who adopted a change package involving clinical pharmacy services [26] and a quality improvement approach based on an adaptation of the Institute of Healthcare Improvement Trigger Tool method. [43]

Profile of professionals involved in the approaches

Of the 23 empirical studies, the majority included a multidisciplinary approach to care provision involving different health professional groups. Four of the approaches also included social workers as part of the team. [24 25 27 29] In eight studies, the approaches included health and social care professionals such as nurses, paramedics, managers, social workers, and allied health professionals; physiotherapists and occupational therapists, pharmacists and optometrists but doctors were not involved. [25 28 29 32-34 36] Many of these studies involved approaches implemented in the community (home setting) or where the home was included as a part of a care pathway.

Measures for assessing impact of the approach

There was heterogeneity in the approaches to measuring safety. We categorised measures as process, outcome or qualitative.

For peer review only - http://bmjopen.bmj.com/site/about/guidelines.xhtml

Two studies included process measures alone. The first involved the monitoring and surveillance of medication safety including the proportion of older patients prescribed falls risk medication, and the proportion of medicines prescribed with have a high potential for major drug interaction. [28] The second study identified measures associated with educational outcomes of health professionals participating in a falls training workshop including subsequent changes to practice. This included workshop participants self-reporting their undertaking of assessments of older patients at higher risk of falls and screening of falls rates. [25]

In 11 studies, outcome measures alone were mentioned. Four of these studies stated falls incidence during follow up over a period of 6-12 months [34 35 46] as a key measure. [48] In three further studies, falls and falls related injuries such as hip fracture, and hospital length of stay were key outcomes [27 44 45]. In four studies, ADE were stated as outcome measures including preventable ADE or potentially preventable ADE, [30 43] proportion of ADE detected in a specified population [26], and ADEs leading to unplanned hospital admission. [41]

Six studies included both process and outcome measures. Process measures associated with falls included adherence to falls prevention strategies, proportion of falls risk assessments undertaken, documenting of falls and medical history risk of falling, and level of independence in activities of daily living. [24 37 39] Outcome measures in these studies included a decrease in falls rates, time between the first and second falls, falls related subsequent emergency department visits and hospitalisation.

Process measures associated with medication safety comprised medication appropriateness and use of harmful medications. [29 40] Outcome measures in these studies were medication related readmissions, rate of hip fractures and clinically significant drug interactions. The systematic review described a range of process and outcome measures across the included studies, all associated with adverse events including ADE. [14]

For peer review only - http://bmjopen.bmj.com/site/about/guidelines.xhtml

291 The remaining studies described qualitative outcomes for the integrative approaches i.e. based on
292 the perceptions of study participants. These included assessing approaches designed to prevent falls
293 such as the perceived usefulness of a MDT home-based fall prevention programme. [31] A further four
294 studies described qualitative outcomes in terms of assessing the quality of medication safety
295 management practices. These included the extent of professional learning experiences to support
296 medicines management, [32] the quality of service delivery of a domiciliary hospitalisation unit and its
297 impact on assuring drug safety, [47] the usefulness of a self-assessment tool in identifying areas of
298 improvement for medication safety [33] and the ability of an E-message system to support information
299 exchange to assure medication safety. [36]

300 Discussion

301 This scoping review brings together the published literature on approaches to improve patient safety
302 in integrated care for community-dwelling adults with long term condtions and demonstrates the
303 range of approaches employed in safety improvement strategies for care across boundaries within
304 several countries. The approached identified in the included studies focus primarily on two common
305 patient safety targets for harm, falls and ADE through falls prevention and medication safety
306 programmes spanning micro, meso and macro levels of a health and care system. Just over a third of
307 the included studies involved the implementation of system-wide approaches (macro level) such as
308 digital shared care records, training/educational initiatives and the strengthening of care pathways
309 between providers. A higher proportion (40%) of studies described micro-level approaches, primarily
310 MDT delivering care to older people in the home setting alone or as part of a care pathway involving
311 hospitals and primary care. The review also highlighted that the evaluation of approaches indicated
312 a perspective of safety focused primarily on measurement - changes in rates of adverse events or
313 contributory factors at individual patient level.

314 A key strength of this study was the highlighting of how key components of integrated care can also
315 improve safety across care boundaries particularly in reducing falls and medication safety

For peer review only - http://bmjopen.bmj.com/site/about/guidelines.xhtml

management. Instituting integrated care practices at the different levels of a care system e.g. digital shared care records (macro), strengthening of care pathways (macro, meso and micro) and promoting MDT working (meso and micro) will strengthen safety approaches and reduce the risk of harm across an ICS. The study findings also highlight how in some cases MDT working, particularly in community care does not directly involve doctors. This reflects an increasing trend in some Western European countries where integrated community care teams (without doctors) are primarily responsible for care provision in the home setting for older and/or frail patients with complex care needs, partly promulgated by the extension of roles of community care professionals and a shortage of primary care doctors. [49 50] The study also identified a range of measures to assess patient safety across systems suggesting that ICS will need to develop a range of indicators to capture the variation in performance of key processes that lead to desired safety outcomes. Another strength of the study was the rigorous approach to scoping and selecting the literature including the comprehensive searching of several academic and grey literature databases and sources. In comparison to the existing literature on this topic, our study builds upon previous work on safety in care transitions but extends beyond the spaces and pathways between organisations at the micro level to consider system-wide (meso and macro) approaches to improving safety.

The study was not without limitations. Articulating a precise definition for patient safety was challenging, as, in several studies, the term was associated with preventing hospitalisation. Hence, we selected the WHO definition which is broad ranging and encompasses the notion of errors of commission and omission. Nonetheless, the identification of relevant studies was hindered by the lack of consistency and interchangeability of the terms ‘integrated care and patient safety’ and their use in the literature. We may have screened for word choice rather than presence or absence of a similar term or its description. Despite including a range of search terms associated with patient safety some studies may have been missed. Our search strategy did not include social care as a specific search term, although integrated care including care coordination and multidisciplinary working increasingly involves social care professionals. We also did not search for person-centred

For peer review only - http://bmjopen.bmj.com/site/about/guidelines.xhtml

care which has emerged as a key tenet for integrated care approaches. This may explain why we were unable to identify any studies reporting the experience of patients/carers. While the review was able to identify the different types of patient safety approaches (scope) it was more challenging to describe their scale across care boundaries.

Integrated care initiatives identified in this study involved the design of a range of new structures and processes to support the delivery of safer care, although further work is required to establish their effectiveness in improving patient safety. Integrated digital care records have been cited as a key enabler for structural integration in ICS as they promote sharing of information thus minimising the risk of care-related information being overlooked as people (particularly older and more frail) move along a care pathway. [51] Training and educational initiatives delivered to inter-professional teams are also considered to be a means of improving the quality of integrated care delivery, supporting the development of social and working relationships thereby promoting partnership working. [52] At all levels, several approaches were implemented to strengthen the care pathway on discharge from hospital and provide tailored care in the home setting to reduce the risk of adverse events. These initiatives had several goals associated with integration, to deliver care closer to home, reduce duplication, minimise the risk of hospital admission and provide personalised care. [3] Each of these goals are notable processes of integration which, if successfully implemented, could also improve patient safety outcomes. These focused approaches do not negate the need for further efforts to develop patient safety within ICS including safety governance at all levels, system-wide mechanisms to identify, review and learn from safety incidents, enhanced capacity and capability for measurement and improvement, and creating learning cultures that promote openness and transparency. [10 53]

The levels of the system (micro, meso and macro) approach to identifying integrated care and patient safety approaches presented in this study provides a useful means of understanding how organisations can implement change, improvement and innovation across an integrated care

For peer review only - http://bmjopen.bmj.com/site/about/guidelines.xhtml

367 system. At the macro level, the implementation of integrated care records to collate information and support communication alongside the promotion of a learning culture supported by inter-professional training and education is required. At the meso level, the focus would be on organisations jointly planning and designing multi-disciplinary approaches to develop seamless care pathways, minimising the risk of harm as people transition between providers. At the micro-level, a range of multi-disciplinary working practices to improve safety could be deployed from near real time safety monitoring within 'community huddles' to protected time sessions to compare and reflect on performance across teams promoting longer term changes. [54] These approaches are potential solutions to the fragmentation of health and care systems (which continues despite efforts to integrate care) and will be integral to improving quality and safety outcomes for increasing populations of people with long term conditions as they receive care closer to home. [10]

378 The studies included in this review evaluated falls reduction and medicines safety approaches using clinical process and outcome measures with several authors looking at associations between these outcomes and wider health system outcomes e.g. hospital (re) admissions and length of stay. System outcome measures have been widely used as performance indicators to assess the impact of integrated care. [3 55] While structural or relational measures of integration such as collaborative and co-terminus working [49 56 57] or the aforementioned system outcomes dominate the discourse of the effects of integrated care, less attention has been paid to measures related to patient or carer experience. [58] None of the studies in this review collected data on the experience of patients and carers alongside clinical outcomes. The understanding of experiences of patients in receipt of services may support service improvement to deliver better quality care. [59 60]

388 A wide range of process and outcome measures were used across the studies predominantly to capture patient harm and contributory factors. Expanding measurement to incorporate the capture of the variation in performance of key processes that lead to desired outcomes, would not only identify the most effective approaches for improving patient safety but also open up a wider range

For peer review only - http://bmjopen.bmj.com/site/about/guidelines.xhtml

392 of options to support building resilience across ICS.[61] A standardised set of safety indicators would
393 support comparisons between different integrated care approaches to safety.

394 Safety approaches in this review were mainly focused on medication safety management and falls
395 prevention which is unsurprising as both account for a notable proportion of unplanned hospital
396 admissions. In England, in 2018 falls accounted for a total of 220,160 hospital admissions in those
397 aged over 65. A study of medication errors in the UK estimated that ADE accounted for 27,362
398 hospital admissions annually, relating to 136,811 bed days at a cost of £83.7 million to the NHS.[62 63]
399 While falls and medication safety are justifiably key safety priorities for integrated care, there is
400 scope to broaden the focus to other common safety issues. For example, a study in the Netherlands
401 found that of 106 admissions to hospital in patients aged over 65 over a period of 12 months were
402 due to an adverse event with 45% were due to procedural related infections, community acquired
403 infection such as pneumonia with/without signs of sepsis and diabetes related hypoglycaemia.[63 64]

404 Future research should focus on the role of other patient safety targets (not just falls and medication
405 related safety) such as healthcare associated infections, community acquired infections and pressure
406 ulcers/sores which also increase the risk of harm for people with long term conditions especially in
407 the community setting. Furthermore, research must establish the effectiveness of the structural and
408 relational aspects of integrated care such as care coordination as well as the approaches identified in
409 this review e.g. integrated care records and education and training, in terms of their impact on
410 reducing the risk of harm across care pathways. This would provide the evidence for policymakers
411 and system leaders to implement the necessary changes to support integration, safety governance
412 and service improvement.

413 Conclusion

414 In order to maximise the potential for ICS to fulfil their promise of improving care for people with
415 long term conditions, approaches such as integrated care records, allocating resources for training

For peer review only - http://bmjopen.bmj.com/site/about/guidelines.xhtml

and education, greater efforts to form robust and risk-limited care pathways as well as support for MDT working, especially in the community setting will have to be key priorities for improving safety across systems. They also align to the goals of ICS thereby reducing fragmentation and enhancing personalisation of care.

Data availability statement

Data sharing not applicable as no datasets were generated and/or analysed for this study. All data relevant to the study are included in the article or uploaded as online supplemental information.

Ethics approval statement

No ethical approval was needed because data from previous published studies are being used.

Funding

This research is funded by the National Institute for Health Research (NIHR) Policy Research Programme, conducted through the Quality, Safety and Outcomes Policy Research Unit, PR-PRU-1217–20702. The views expressed are those of the author(s) and not necessarily those of the NIHR or the Department of Health and Social Care.

Competing interests

None.

Author Contributions

ML conceived and designed the study. All authors contributed to several aspects of the review process i.e. stages 1-5 described in the manuscript under the methods section. ML and HH identified the research question. ML developed the search strategy (with support from HH) and identified relevant studies. ML and SW were involved study selection, charting of the data from included studies and results collation, reporting and summarising of the data. ML drafted the manuscript. All authors approved the final version of the manuscript.

For peer review only - http://bmjopen.bmj.com/site/about/guidelines.xhtml

References

1. Armitage GD, Suter E, Oelke ND, et al. Health systems integration: state of the evidence. *Int J Integr Care* 2009;9(2):e82. doi: 10.5334/ijic.316 [published Online First: 2009/07/11]
2. Stange KC. The problem of fragmentation and the need for integrative solutions. *Annals of family medicine* 2009;7(2):100-03. doi: 10.1370/afm.971
3. Baxter S, Johnson M, Chambers D, et al. The effects of integrated care: a systematic review of UK and international evidence. *BMC health services research* 2018;18(1):350. doi: 10.1186/s12913-018-3161-3
4. Ham C, Dixon J, Chantler C. Clinically integrated systems: the future of NHS reform in England? *BMJ (Clinical research ed)* 2011;342:d905. doi: 10.1136/bmj.d905 [published Online First: 2011/03/30]
5. Waring J, Marshall F, Bishop S. Understanding the occupational and organizational boundaries to safe hospital discharge. *Journal of Health Services Research and Policy* 2015;20(1 Suppl):35-44. doi: 10.1177/1355819614552512 [published Online First: 2014/12/05]
6. Glasby J. If Integration Is the Answer, What Was the Question? What next for English Health and Social Care Partnerships? *Int J Integr Care* 2016;16(4):11. doi: 10.5334/ijic.2535 [published Online First: 2017/03/21]
7. Shaw S, Rosen R, Rumbold B. What is Integrated Care? London: Nuffield Trust, 2011:1-23.
8. WHO. Patient safety: World Health Organisation; 2021 [Available from: https://www.who.int/teams/integrated-health-services/patient-safety.
9. Valentijn PP, Schepman SM, Opheij W, et al. Understanding integrated care: a comprehensive conceptual framework based on the integrative functions of primary care. *Int J Integr Care* 2013;13:e010. doi: 10.5334/ijic.886 [published Online First: 2013/05/21]
10. Auraaen A, Saar K, Klazinga N. System governance towards improved patient safety: OECD, 2020.

For peer review only - http://bmjopen.bmj.com/site/about/guidelines.xhtml

11. Waring J, Bishop S. Health States of Exception: unsafe non-care and the (inadvertent) production of 'bare life' in complex care transitions. *Sociology of Health and Illness* 2020;42(1):171-90. doi: 10.1111/1467-9566.12993 [published Online First: 2019/09/19]
12. Illingworth J. Is the NHS getting safer? London, UK: The Health Foundation, 2015.
13. Vincent C, Burnett S, Carthey J. The measurement and monitoring of safety. London: The Health Foundation, 2013.
14. Laugaland K, Aase K, Barach P. Interventions to improve patient safety in transitional care--a review of the evidence. *Work (Reading, Mass)* 2012;41 Suppl 1:2915-24. doi: 10.3233/WOR-2012-0544-2915 [published Online First: 2012/02/10]
15. Aase K, Schibevaag L, Waring J. Crossing boundaries: quality in care transitions. Researching Quality in Care Transitions: Springer 2017:3-29.
16. King BJ, Gilmore-Bykovskyi AL, Roiland RA, et al. The consequences of poor communication during transitions from hospital to skilled nursing facility: a qualitative study. *Journal of the American Geriatrics Society* 2013;61(7):1095-102. doi: 10.1111/jgs.12328 [published Online First: 2013/06/03]
17. Peters MD, Godfrey CM, Khalil H, et al. Guidance for conducting systematic scoping reviews. *International Journal of Evidence Based Healthcare* 2015;13(3):141-6. doi: 10.1097/xeb.0000000000000050 [published Online First: 2015/07/03]
18. Tricco AC, Lillie E, Zarin W, et al. PRISMA Extension for Scoping Reviews (PRISMA-ScR): Checklist and Explanation. *Annals of internal medicine* 2018;169(7):467-73. doi: 10.7326/m18-0850 [published Online First: 2018/09/05]
19. Improvement N. The how-to guide for measurement for improvement: NHS Improvement, 2020.
20. Curry N, Ham C. Clinical and service integration: The King's Fund, 2010.
21. Donabedian A. Evaluating the quality of medical care. *The Milbank memorial fund quarterly* 1966;44(3):166-206.
22. Duhn L, Godfrey C, Medves J. Scoping review of patients' attitudes about their role and behaviours to ensure safe care at the direct care level. *Health Expectations* 2020;23(5):979-91. doi: https://doi.org/10.1111/hex.13117
23. Cheraghi-Sohi S, Panagioti M, Daker-White G, et al. Patient safety in marginalised groups: a narrative scoping review. *International journal for equity in health* 2020;19(1):26-26. doi: 10.1186/s12939-019-1103-2
24. Eckstrom E, Neal MB, Cotrell V, et al. An Interprofessional Approach to Reducing the Risk of Falls Through Enhanced Collaborative Practice. *Journal of the American Geriatrics Society* 2016;64(8):1701-7. doi: 10.1111/jgs.14178 [published Online First: 2016/07/29]
25. McKenzie G, Lasater K, Delander GE, et al. Falls prevention education: Interprofessional training to enhance collaborative practice. *Gerontology & Geriatrics Education* 2017;38(2):232-43. doi: 10.1080/02701960.2015.1127809 [published Online First: 2016/02/18]
26. Robbins CM, Stillwell T, Johnson D, et al. Integrating patient safety and clinical pharmacy services into the care of a high-risk, ambulatory population: a collaborative approach. *Journal of patient safety* 2013;9(2):110-7. doi: 10.1097/PTS.0b013e318281b879 [published Online First: 2013/05/24]
27. Wallace R, Angus LDG, Munnangi S, et al. Improved outcomes following implementation of a multidisciplinary care pathway for elderly hip fractures. *Aging clinical and experimental research* 2019;31(2):273-78. doi: 10.1007/s40520-018-0952-7 [published Online First: 2018/04/25]
28. Willis JS, Hoy RH, Jenkins WD. In-home medication reviews: a novel approach to improving patient care through coordination of care. *Journal of community health* 2011;36(6):1027-31. doi: 10.1007/s10900-011-9405-3 [published Online First: 2011/04/19]
29. McQuaid-Bascon K, Royal M, Sinno M, et al. Evolving a multi-factorial, data driven, interprofessional approach to prevent falls and associated injuries during a system-level integration. *Journal of Interprofessional Education* 2018;12:8-12.

For peer review only - http://bmjopen.bmj.com/site/about/guidelines.xhtml

30. Akbarov A, Kontopantelis E, Sperrin M, et al. Primary Care Medication Safety Surveillance with Integrated Primary and Secondary Care Electronic Health Records: A Cross-Sectional Study. *Drug safety* 2015;38(7):671-82. doi: 10.1007/s40264-015-0304-x [published Online First: 2015/06/24]
31. Amacher AE, Nast I, Zindel B, et al. Experiences of general practitioners, home care nurses, physiotherapists and seniors involved in a multidisciplinary home-based fall prevention programme: a mixed method study. *BMC health services research* 2016;16 doi: 10.1186/s12913-016-1719-5
32. Bell HT, Granas AG, Enmarker I, et al. Nurses' and pharmacists' learning experiences from participating in interprofessional medication reviews for elderly in primary health care - a qualitative study. *BMC family practice* 2017;18(1):30. doi: 10.1186/s12875-017-0598-0 [published Online First: 2017/03/01]
33. Brito AM, Simoes AM, Alcobia A, et al. Optimising patient safety using pharmaceutical intervention in domiciliary hospitalization. *International journal of clinical pharmacy* 2017;39(5):980-84. doi: 10.1007/s11096-017-0512-9 [published Online First: 2017/08/26]
34. Di Monaco M, Vallero F, De Toma E, et al. A single home visit by an occupational therapist reduces the risk of falling after hip fracture in elderly women: A quasi-randomized controlled trial. *Journal of Rehabilitation Medicine* 2008;40(6):446-50. doi: 10.2340/16501977-0206
35. Di Pollina L, Guessous I, Petoud V, et al. Integrated care at home reduces unnecessary hospitalizations of community-dwelling frail older adults: a prospective controlled trial. *BMC geriatrics* 2017;17(1):53-53. doi: 10.1186/s12877-017-0449-9
36. Foged S, Norholm V, Andersen O, et al. Nurses' perspectives on how an e-message system supports cross-sectoral communication in relation to medication administration: A qualitative study. *Journal of clinical nursing* 2018;27(3-4):795-806. doi: 10.1111/jocn.14109 [published Online First: 2017/10/20]
37. Johansen JS, Havnes K, Halvorsen KH, et al. Interdisciplinary collaboration across secondary and primary care to improve medication safety in the elderly (IMMENSE study): study protocol for a randomised controlled trial. *BMJ open* 2018;8(1):e020106. doi: 10.1136/bmjopen-2017-020106 [published Online First: 2018/01/25]
38. Lenander C, Bondesson A, Midlov P, et al. Healthcare system intervention for safer use of medicines in elderly patients in primary care-a qualitative study of the participants' perceptions of self-assessment, peer review, feedback and agreement for change. *BMC family practice* 2015;16(1):117. doi: 10.1186/s12875-015-0334-6 [published Online First: 2015/09/05]
39. Peeters GM, de Vries OJ, Elders PJ, et al. Prevention of fall incidents in patients with a high risk of falling: design of a randomised controlled trial with an economic evaluation of the effect of multidisciplinary transmural care. *BMC geriatrics* 2007;7:15. doi: 10.1186/1471-2318-7-15 [published Online First: 2007/07/04]
40. Toivo T, Airaksinen M, Dimitrow M, et al. Enhanced coordination of care to reduce medication risks in older home care clients in primary care: a randomized controlled trial. *BMC geriatrics* 2019;19(1):332. doi: 10.1186/s12877-019-1353-2 [published Online First: 2019/11/30]
41. van Melle MA, Zwart DLM, Poldervaart JM, et al. Validity and reliability of a medical record review method identifying transitional patient safety incidents in merged primary and secondary care patients' records. *BMJ open* 2018;8(8):e018576. doi: 10.1136/bmjopen-2017-018576 [published Online First: 2018/08/15]
42. van Melle MA, van Stel HKF, Poldervaart JM, et al. Measurement tools and outcome measures used in transitional patient safety; a systematic review. *PloS one* 2018;13(6) doi: 10.1371/journal.pone.0197312
43. Khoo AL, Teng M, Lim BP, et al. A multicenter, multidisciplinary, high-alert medication collaborative to improve patient safety: the Singapore experience. *Joint Commission journal*

For peer review only

on quality and patient safety 2013;39(5):205-12. doi: 10.1016/s1553-7250(13)39027-8 [published Online First: 2013/06/12]
44. Sze PC, Cheung WH, Lam PS, et al. The efficacy of a multidisciplinary falls prevention clinic with an extended step-down community program. *Archives of Physical Medicine and Rehabilitation* 2008;89(7):1329-34. doi: 10.1016/j.apmr.2007.11.044 [published Online First: 2008/07/01]
45. Mikolaizak AS, Lord SR, Tiedemann A, et al. A multidisciplinary intervention to prevent subsequent falls and health service use following fall-related paramedic care: a randomised controlled trial. *Age and ageing* 2016;46(2):200-07. doi: 10.1093/ageing/afw190
46. Hendriks MR, Bleijlevens MH, van Haastregt JC, et al. Lack of effectiveness of a multidisciplinary fall-prevention program in elderly people at risk: a randomized, controlled trial. *Journal of the American Geriatrics Society* 2008;56(8):1390-7. doi: 10.1111/j.1532-5415.2008.01803.x [published Online First: 2008/07/30]
47. Lenander C, Bondesson Å, Viberg N, et al. Effects of an intervention (SÄKLÄK) on prescription of potentially inappropriate medication in elderly patients. *Family practice* 2017;34(2):213-18. doi: 10.1093/fampra/cmw121
48. Noor H, et al. The development, implementation and evaluation of a transitional care programme to improve outcomes of frail older patients after hospitalisation. *Age and ageing* 2016;45(5):643-51.
49. Lalani M, Marshall M. Co-location, an enabler for service integration? Lessons from an evaluation of integrated community care teams in East London. *Health & social care in the community* 2022;30(2):e388-e96. doi: 10.1111/hsc.13211 [published Online First: 2020/11/06]
50. Drennan VM, Calestani M, Ross F, et al. Tackling the workforce crisis in district nursing: can the Dutch Buurtzorg model offer a solution and a better patient experience? A mixed methods case study. *BMJ open* 2018;8(6)
51. Murray R, Mckenna H. The Next Steps Towards Integrated Care: The King's Fund, 2020.
52. Jeffs L, Abramovich IA, Hayes C, et al. Implementing an interprofessional patient safety learning initiative: insights from participants, project leads and steering committee members. *BMJ quality & safety* 2013;22(11):923-30. doi: 10.1136/bmjqs-2012-001720 [published Online First: 2013/06/19]
53. OECD. Measuring Patient Safety: Opening the Black Box. France, 2018.
54. Montague J, Crosswaite K, Lamming L, et al. Sustaining the commitment to patient safety huddles: insights from eight acute hospital ward teams. *British Journal of Nursing* 2019;28(20):1316-24.
55. Kelly L, Harlock J, Peters M, et al. Measures for the integration of health and social care services for long-term health conditions: a systematic review of reviews. *BMC health services research* 2020;20(1):358. doi: 10.1186/s12913-020-05206-5
56. Kirst M, Im J, Burns T, et al. What works in implementation of integrated care programs for older adults with complex needs? A realist review. *International Journal for Quality in Health Care* 2017;29(5):612-24.
57. Bussu S, Marshall M. Organisational development to support integrated care in East London: the perspective of clinicians and social workers on the ground. *Journal of health organization and management* 2020;34(5):603-19. doi: 10.1108/jhom-10-2019-0294 [published Online First: 2020/07/19]
58. Crocker H, Kelly L, Harlock J, et al. Measuring the benefits of the integration of health and social care: qualitative interviews with professional stakeholders and patient representatives. *BMC health services research* 2020;20(1):515. doi: 10.1186/s12913-020-05374-4
59. Robert G, Donetto S. Whose data is it anyway? Patient experience and service improvement. 2020;25(3):139-41.
60. Thorstensen-Woll C. Understanding integration: how to listen to and learn from people and communities The King's Fund, 2021.

For peer review only - http://bmjopen.bmj.com/site/about/guidelines.xhtml

61. Smith KM, Valenta AL. Safety I to Safety II: A Paradigm Shift or More Work as Imagined?: Comment on" False Dawns and New Horizons in Patient Safety Research and Practice". *International Journal of Health Policy and Management* 2018;7(7):671.
62. England PH. Falls: Applying All Our Health: Public Health England, 2020.
63. Elliott R, Camacho E, Campbell F, et al. Prevalence and economic burden of medication errors in the NHS in England. 2018
64. Magdelijns FJH, van Avesaath REM, Pijpers E, et al. Health-care-related adverse events leading to admission in older individuals: incidence, predictive factors and consequences. *European journal of public health* 2016;26(5):743-48. doi: 10.1093/eurpub/ckw021

For peer review only

For peer review only - http://bmjopen.bmj.com/site/about/guidelines.xhtml

Figure 1: Flowchart of study selection

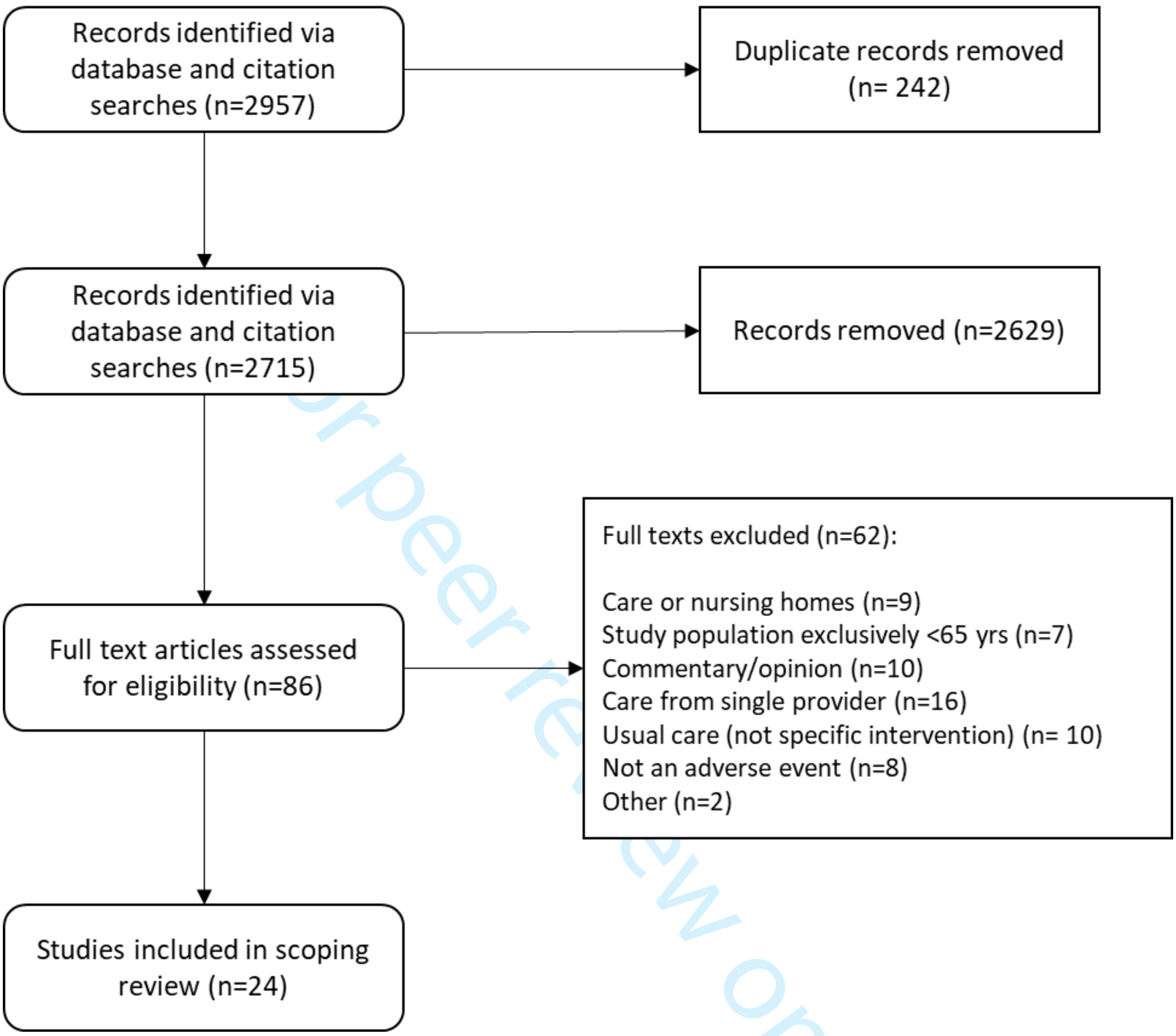

Additional File 1 – search strategy

Search terms (including MeSH) used were:

elderly (elder*) OR older OR older adults OR geriatric OR aged

vulnerable adults

mental health OR disorder

learning disabilities (*disability)

(-term OR long term) conditions

complex care

chronic conditions

multi-morbidity

AND

integrated care

care pathways

multidisciplinary OR multi-disciplinary

multiprofessional OR multi-professional

interprofessional OR inter-professional

transitional (W#) care (transitio*)

care (W#) coordination

AND

patient safety

preventable (w#) harm

risk reduction

adverse events

For peer review only

Supplementary table: Characteristics and description of included studies

Author and year	Country	Aim	Population	Study design	Integrated care approach				Approaches to measuring safety
					Type of approach	Description of approach	Setting	Level	
**Akbarov 2015 (36)**	England	Investigate the feasibility of wide ranging medication safety surveillance using a linked database	Adults with prescribing hazards	Cross-sectional study	Integrated digital record	Linked records of patients served by one hospital system and primary care centres - prevalence of 22 medication safety indicators, investigation of associations with patient and practice characteristics, and variation between primary care practices	Hospitals and primary care centres	Macro	Outcome measure: Proportion of patients with at least one medication safety hazard
**Amacher 2016 (37)**	Switzerland	Explore the perceived benefits and barriers of an evidence-based, home-based pilot falls prevention programme	Elderly	Mixed methods	MDT approach to care delivery in community (home) or primary care setting	Multi-factorial falls prevention programme (FPP)	Community (home)	Micro	Qualitative outcome: Usefulness of MDT home-based fall prevention programme
**Bell 2017 (38)**	Norway	Investigate nurses' and pharmacists' perceived learning experience after participating in an Integrated Medicines Management Review in primary health care	Elderly	Qualitative study	MDT approach to care delivery in community (home) or primary care setting	Integrated Medicines Management model – centred on inter-professional medication reviews	Community (home based services and nursing homes)	Macro	Qualitative outcome: Extent of professional learning experiences to support medicines management

For peer review only

**Brito 2017 (39)**	Portugal	Managing medication, in a patient-centred process and multidisciplinary context, by gathering information about the drug therapy of patients before admission, during hospitalization and after hospital discharge	Elderly	Observation study	Care pathway	Pharmacist working within domiciliary hospitalisation unit (DHU) alongside a multi-disciplinary team – medication reconciliation and review	Community (home) – domiciliary hospitalisatio n	Micro	Qualitative outcome: Perceived quality of DHU delivery and its impact on drug safety
**Di Monaco 2008 (40)**	Italy	Assessment of the effectiveness of a follow up home visit by an occupational therapist after a fall. MDT prevention intervention to reduce falls risk after hip fracture in elderly women	60 years or older	Quasi-RCT	Care pathway	MDT approach to prevent falls; exercise, advice and training on use of assistive devices, and activities of daily living	Hospital and community (home)	Micro	Outcome measure: Occurrence of falls in six months post-discharge from hospital
**Di Pollina 2017 (41)**	Switzerland	Testing the efficacy of providing integrated care at home to reduce unnecessary hospitalisations, emergency room visits, institutionalisation, and mortality in community	Frail and dependent people over 60 years	Prospective RCT	MDT approach to care delivery in community (home) or primary care setting	Integrated in-home geriatric assessments, long-term coordinated follow-up, and availability of a round-the-clock geriatric call service	Community (home)	Micro	Outcome measure: Falls over 3 years as a secondary outcome (stated as reason for hospitalisation)

For peer review only

		dwelling frail older adults							
**Eckstrom 2016 (30)**	US	Establishing changes in team behaviours after the intervention communicate and enhance adherence to Geriatrics Society Falls Prevention Guidelines by inter-professional teams in practice) and highlight lessons learned during the practice change process	Elderly patients	Mixed methods	Educational approach	Falls prevention strategy training workshops to clinical inter-professional teams – content based on guidelines developed by American Geriatric Society and British Geriatric Society	Hospitals and community or primary care clinics	Macro	Process measure: Adherence to falls prevention strategies<br><br>Outcome measure: Rate of falls, emergency department visits for falls, and hospitalisations for falls over a three month period
**Foged 2018 (42)**	Denmark	Describe nurse perspectives on how an e-message system supports communication on medication administration between hospital and home care nurses	Elderly	Qualitative	Integrated digital record	E-message system - admission report, care plan, discharge report, discharge notification and a message platform to bolster communication between hospital nurses and nurses providing home-based care	Hospital and community (home)	Macro	Qualitative outcome: The ability of an E-message system to support information exchange to assure medication safety
**Hendriks 2008 (52)**	Netherlands	Assess whether a MDT fall-prevention program was more effective than standard care in preventing new falls and functional decline in elderly community-dwelling people	Adults, 65 years and over	RCT	Care pathway	MDT in hospital providing a falls risk assessment, with referral to GP depending on risk and occupational therapy visits for a short period at home	Hospital and community (home)	Micro	Outcome, Primary measure: number of falls experienced in 12 month follow up period

For peer review only

		who attended an emergency department after a fall							
**Hiem 2016 (54)**	Netherlands	Decrease the risk of functional decline and loss of independence after hospitalisation in frail older patients	Elderly patients	Action research	Care pathway	Various approaches across primary and acute care to support frail patients. Approaches address (i) improved risk management; (ii) delivery of integrated, function-oriented care; (iii) specific geriatric approaches; and (iv) optimisation of transfers	Primary care, nursing home, rehabilitation centres and hospitals	Macro	Outcome: Incidence of adverse outcomes including falls in the last six months
**Johansen 2018 (43)**	Norway	Explore the effects of the intervention on healthcare use, health-related quality of life and medication appropriateness in elderly patients	70 yrs and over	Protocol RCT	Care pathway	Integrated medicines management model adapted to improve both medication safety in hospital and communication across primary and secondary care. Medication reconciliation (admission), medication review (during hospital stay), patient counselling (medicines use), medication list with explanations in discharge summary and post-discharge phone calls to primary care	Secondary and primary care	Meso	Secondary process measure: medication appropriateness, medication changes,<br><br>Outcome measure: hip fracture rate, medication-related readmissions

**Khoo 2013 (49)**	Singapore	Reduce adverse drug events resulting from high-alert medication	Adults on 'high risk of adverse events' medications	QI case study	Care pathway	Multicentre multidisciplinary collaborative focused on improving safe use of high-alert medication. QI approach with tracking of improvement conducted with an adapted IHI Trigger Tool method	Primary and acute care institutions	Macro	Outcome measure: Preventable or potentially preventable adverse drug events
**Laugaland 2012 (16)**	Global	Identify and evaluate the effects of the interventions in terms of effectiveness and efficiency of care processes in the context of effects of discharge interventions on patient safety	Patients 65 years and over	Review	Care pathway	Several studies that employed MDT approaches to transitional care to improve patient safety	Hospital, community and primary care	Meso	Process and outcome measures associated with adverse events
**Lenander 2015 (53)**	Sweden	Evaluate participants' perceptions of the SÄKLÄK project, which aims to enhance medication safety, especially for elderly patients, in primary care	Elderly	Qualitative study	MDT approach to care delivery in community (home) or primary care setting	SÄKLÄK intervention model involves a multidisciplinary group with self-assessment, peer review, feedback and agreement for change	Primary care	Meso	Qualitative outcome: Usefulness of self-assessment tool in identifying areas of improvement for medication safety

For peer review only

**Mckenzie 2017 (31)**	US	Describe an inter-professional team-based training approach to falls prevention for older adults and the related educational outcomes	Older adults	Service evaluation	Educational approach	Project that involves an inter-professional teaching team to support practice teams to reduce falls in older adults via implementation of evidence-based practice guidelines	Ambulatory, long term care, hospital, and home health practice	Macro	Process measure: Associated with educational outcomes resulting from falls training workshops; knowledge, learner confidence, intention for collaborative practice change and change to practice (falls screening rates and falls risk assessments)
**McQuaid-Bascon 2018 (35)**	Canada	An overview of a recently integrated health care system's experience in evolving their multi-component inter –professional approach to falls prevention	Older patients susceptible to falls	Service evaluation	Care pathway	Establishment of multidisciplinary falls committee to provide leadership, direction and coordination in the inter-professional advancement of quality improvement, evidence-informed practices and research associated with falls prevention and injury minimisation	Hospital	Meso	Process measure: i) sustain falls risk assessment and falls communication bundle across all areas; ii) monitor and report falls assessment rates and bundle implementation on inpatient areas; and iii) explore patient video monitoring and soft cell flooring<br><br>Outcome measure: improve falls with injury rates by 25%;
**Mikolaizak 2016 (51)**	Australia	Determine whether an approach involving timely assessment and tailored interventions, resulted in a meaningful reduction in subsequent falls, fall-related injuries and associated emergency health service use in older people who were	Older adults 65 years and over	RCT	MDT approach to care delivery in community (home) or primary care setting	Targeted identified risk factors and used existing services to implement physiotherapy, occupational therapy, geriatric assessment, optometry and medication management approaches to prevent falls	Community (home)	Micro	Outcome measure: Rate of falls and fall-related injuries during the 12-month follow-up period

For peer review only

		not transported to hospital following a fall							
**Peeters 2007 (45)**	Netherlands	Reduce the fall risk in older persons with a high risk of falling. The intervention consists of a systematic assessment of the putative causes of falling and subsequent targeted individualised preventive measures	Older adults >65	RCT	Care pathway	Assessment by geriatric outpatient clinic for falls risk factors in collaboration with a MDT that provides multifactorial treatment that may include physiotherapy, ophthalmic assessment and medicines management	Community (home)	Micro	Process measure: proportion assessed for; risk of recurrent falling, fall history, medical history, medication use, independence in activities of daily living<br>Outcome measure: Time to first and second fall
**Robbins 2013 (32)**	US	To describe the process followed and outcomes achieved from participating in a pharmacy clinical services and patient safety collaborative and integrating pharmacy services into a high-risk, ambulatory population	Adults	QI case study	Care pathway	Patient Safety and Clinical Pharmacy Services Collaborative change package involving a MDT	Health centre	Macro	Outcome measure: Percentage of adverse drug events detected in the population of focus that received clinical pharmacy services during the month of follow up
**Sze 2008 (50)**	Hong Kong	To investigate the efficacy of a falls prevention clinic and a community step-down programme in	Elderly patients	Prospective cohort	MDT approach to care delivery in community (home) or	12 week falls clinic provided by a MDT - including fall evaluation, balance training, home hazard	Community (home)	Micro	Primary outcome measure: Number of falls per person per year<br><br>Secondary outcome measure: Fall-related injuries

		reducing the number of falls among community-dwelling elderly at high risk of a fall			primary care setting	management program, and medical referrals. Followed by a community step-down (nine months) program, including falls prevention education, a weekly exercise class, and two home visitations			
**Toivo 2019 (46)**	Finland	To assess the impact of a care coordination intervention on medication risks identified in drug regimens of older home care clients over a one-year period	Elderly patients	Cluster RCT	MDT approach to care delivery in community (home) or primary care setting	Coordinated medication risk management (CoMM) model which involves a multi-stage approach; triage, medication risk screening and reviews, actions plans delivered by a MDT	Community (home)	Micro	Process measure: Several including use of harmful medications<br><br>Outcome: clinically significant drug interactions
**Van Melle 2018 (47)**	Netherlands	To pilot a review of medical records to identify transitional safety incidents (TSIs) for use in a large intervention study and assess its reliability and validity.	Adults under cardiology and gastro service care	Retrospective medical record review study	Integrated digital record	Transitional medical record system: establish inter-rater reliability of a measurement tool designed to assess transitional safety incidents	Hospital and primary care	Macro	Outcome measures: Transitional safety incidents which include but not restricted to adverse drug events leading to unplanned hospital admission
**Wallace 2018 (33)**	US	To evaluate the effects of a multidisciplinary hip fracture care pathway on the outcomes of the elderly patients	Elderly	Retrospective analysis	Care pathway	Multidisciplinary care pathway for reduce hip fractures in the elderly	Hospital (Trauma centre)	Micro	Outcome measures: Several – includes mechanism of injury (such as falls), hip fracture characteristics and hospital length of stay

For peer review only

**Willis 2011 (34)**	US	To determine if trained undergraduate students, in conjunction with pharmacists, could provide in home medication reviews, and demonstrate benefit to the health and welfare of a senior population affiliated Patient-Centred Medical Home	Patients aged 65 years and over	Service evaluation	MDT approach to care delivery in community (home) or primary care setting	Patient-Centred Medical Home model - In-home medication reviews, assessing a home for fall risk, and performing blood pressures	Community (home)	Meso	Process measures: Proportion of patients prescribed falls risk medication and medicines that have a high potential for major drug interaction. Proportion of patients that have had a medication change made to their clinical medication list

For peer review only

Preferred Reporting Items for Systematic reviews and Meta-Analyses extension for Scoping Reviews (PRISMA-ScR) Checklist

SECTION	ITEM	PRISMA-ScR CHECKLIST ITEM	REPORTED ON PAGE #
**TITLE**			
Title	1	Identify the report as a scoping review.	
**ABSTRACT**			
Structured summary	2	Provide a structured summary that includes (as applicable): background, objectives, eligibility criteria, sources of evidence, charting methods, results, and conclusions that relate to the review questions and objectives.	
**INTRODUCTION**			
Rationale	3	Describe the rationale for the review in the context of what is already known. Explain why the review questions/objectives lend themselves to a scoping review approach.	
Objectives	4	Provide an explicit statement of the questions and objectives being addressed with reference to their key elements (e.g., population or participants, concepts, and context) or other relevant key elements used to conceptualize the review questions and/or objectives.	
**METHODS**			
Protocol and registration	5	Indicate whether a review protocol exists; state if and where it can be accessed (e.g., a Web address); and if available, provide registration information, including the registration number.	
Eligibility criteria	6	Specify characteristics of the sources of evidence used as eligibility criteria (e.g., years considered, language, and publication status), and provide a rationale.	
Information sources*	7	Describe all information sources in the search (e.g., databases with dates of coverage and contact with authors to identify additional sources), as well as the date the most recent search was executed.	
Search	8	Present the full electronic search strategy for at least 1 database, including any limits used, such that it could be repeated.	
Selection of sources of evidence†	9	State the process for selecting sources of evidence (i.e., screening and eligibility) included in the scoping review.	
Data charting process‡	10	Describe the methods of charting data from the included sources of evidence (e.g., calibrated forms or forms that have been tested by the team before their use, and whether data charting was done independently or in duplicate) and any processes for obtaining and confirming data from investigators.	
Data items	11	List and define all variables for which data were sought and any assumptions and simplifications made.	
Critical appraisal of individual sources of evidence§	12	If done, provide a rationale for conducting a critical appraisal of included sources of evidence; describe the methods used and how this information was used in any data synthesis (if appropriate).	
Synthesis of results	13	Describe the methods of handling and summarizing the data that were charted.	

For peer review only

St. Michael's
Inspired Care.
Inspiring Science.

For peer review only - http://bmjopen.bmj.com/site/about/guidelines.xhtml

SECTION	ITEM	PRISMA-ScR CHECKLIST ITEM	REPORTED ON PAGE #
**RESULTS**			
Selection of sources of evidence	14	Give numbers of sources of evidence screened, assessed for eligibility, and included in the review, with reasons for exclusions at each stage, ideally using a flow diagram.	
Characteristics of sources of evidence	15	For each source of evidence, present characteristics for which data were charted and provide the citations.	
Critical appraisal within sources of evidence	16	If done, present data on critical appraisal of included sources of evidence (see item 12).	
Results of individual sources of evidence	17	For each included source of evidence, present the relevant data that were charted that relate to the review questions and objectives.	
Synthesis of results	18	Summarize and/or present the charting results as they relate to the review questions and objectives.	
**DISCUSSION**			
Summary of evidence	19	Summarize the main results (including an overview of concepts, themes, and types of evidence available), link to the review questions and objectives, and consider the relevance to key groups.	
Limitations	20	Discuss the limitations of the scoping review process.	
Conclusions	21	Provide a general interpretation of the results with respect to the review questions and objectives, as well as potential implications and/or next steps.	
**FUNDING**			
Funding	22	Describe sources of funding for the included sources of evidence, as well as sources of funding for the scoping review. Describe the role of the funders of the scoping review.	

JBI = Joanna Briggs Institute; PRISMA-ScR = Preferred Reporting Items for Systematic reviews and Meta-Analyses extension for Scoping Reviews.

* Where *sources of evidence* (see second footnote) are compiled from, such as bibliographic databases, social media platforms, and Web sites.

† A more inclusive/heterogeneous term used to account for the different types of evidence or data sources (e.g., quantitative and/or qualitative research, expert opinion, and policy documents) that may be eligible in a scoping review as opposed to only studies. This is not to be confused with *information sources* (see first footnote).

‡ The frameworks by Arksey and O'Malley (6) and Levac and colleagues (7) and the JBI guidance (4, 5) refer to the process of data extraction in a scoping review as data charting.

§ The process of systematically examining research evidence to assess its validity, results, and relevance before using it to inform a decision. This term is used for items 12 and 19 instead of "risk of bias" (which is more applicable to systematic reviews of interventions) to include and acknowledge the various sources of evidence that may be used in a scoping review (e.g., quantitative and/or qualitative research, expert opinion, and policy document).

From: Tricco AC, Lillie E, Zarin W, O'Brien KK, Colquhoun H, Levac D, et al. PRISMA Extension for Scoping Reviews (PRISMAScR): Checklist and Explanation. Ann Intern Med. 2018;169:467–473. doi: 10.7326/M18-0850.

For peer review only - http://bmjopen.bmj.com/site/about/guidelines.xhtml

BMJ Open

Approaches to improving patient safety in integrated care: a scoping review

Journal:	*BMJ Open*
Manuscript ID	bmjopen-2022-067441.R2
Article Type:	Original research
Date Submitted by the Author:	22-Feb-2023
Complete List of Authors:	Lalani, Mirza ; London School of Hygiene and Tropical Medicine Department of Health Services Research and Policy<br>Wytrykowski, Sarah; Medical Research Network<br>Hogan, Helen; London School of Hygiene & Tropical Medicine, Health Services Research and Policy
**Primary Subject Heading**:	Health services research
Secondary Subject Heading:	Health services research
Keywords:	Organisation of health services < HEALTH SERVICES ADMINISTRATION & MANAGEMENT, Quality in health care < HEALTH SERVICES ADMINISTRATION & MANAGEMENT, Health Services for the Aged

SCHOLARONE™
Manuscripts

I, the Submitting Author has the right to grant and does grant on behalf of all authors of the Work (as defined in the below author licence), an exclusive licence and/or a non-exclusive licence for contributions from authors who are: i) UK Crown employees; ii) where BMJ has agreed a CC-BY licence shall apply, and/or iii) in accordance with the terms applicable for US Federal Government officers or employees acting as part of their official duties; on a worldwide, perpetual, irrevocable, royalty-free basis to BMJ Publishing Group Ltd ("BMJ") its licensees and where the relevant Journal is co-owned by BMJ to the co-owners of the Journal, to publish the Work in this journal and any other BMJ products and to exploit all rights, as set out in our licence.

The Submitting Author accepts and understands that any supply made under these terms is made by BMJ to the Submitting Author unless you are acting as an employee on behalf of your employer or a postgraduate student of an affiliated institution which is paying any applicable article publishing charge ("APC") for Open Access articles. Where the Submitting Author wishes to make the Work available on an Open Access basis (and intends to pay the relevant APC), the terms of reuse of such Open Access shall be governed by a Creative Commons licence – details of these licences and which Creative Commons licence will apply to this Work are set out in our licence referred to above.

Other than as permitted in any relevant BMJ Author's Self Archiving Policies, I confirm this Work has not been accepted for publication elsewhere, is not being considered for publication elsewhere and does not duplicate material already published. I confirm all authors consent to publication of this Work and authorise the granting of this licence.

For peer review only

Approaches to improving patient safety in integrated care: a scoping review

Mirza Lalani,[1*] Sarah Wytrykowski,[2] Helen Hogan[1]

[1] Department of Health Services Research and Policy, London School of Hygiene and Tropical Medicine

[2] Medical Research Network

*Corresponding author: Mirza Lalani, mirza.lalani@lshtm.ac.uk

For peer review only

Abstract

Objectives: This scoping review aimed to establish the approaches employed to improving patient safety in integrated care for community dwelling adults with long term conditions

Design: Scoping Review

Setting: All care settings

Search strategy: Systematic searches of seven academic and grey literature databases for studies published between 2000-2021. At the full text review stage both the first and second reviewer (SW) independently assessed full texts against the eligibility criteria and any discrepancies were discussed

Results: Overall, 24 studies were included in the review. Two key priorities for safety across care boundaries for adults with long term conditions were falls and medication safety. Approaches for these priorities were implemented at different levels of an integrated care system. At the micro level, approaches involved care primarily in the home setting provided by multi-disciplinary teams. At the meso-level, the focus was on planning and designing approaches at the managerial/organisational level to deliver multi-disciplinary care. At the macro level, system-wide approaches included integrated care records, training and education and the development of care pathways involving multiple organisations. Across the included studies evaluation of these approaches was undertaken using a wide range of process and outcome measures to capture patient harm and contributory factors associated with falls and medication safety

For peer review only - http://bmjopen.bmj.com/site/about/guidelines.xhtml

Conclusions:. For integrated care initiatives to fulfil their promise of improving care for adults with long term conditions, approaches to improve patient safety need to be instituted across the system, at all levels to support the structural and relational aspects of integrated care as well as specific risk-related safety improvements.

Keywords: Patient safety, integrated care, falls, medication safety

Word count: 4920

Strengths and limitations:

- Highlighting how key components of integrated care can also improve patient safety across care boundaries particularly in reducing falls and medication safety management
- Another strength of the study was the rigorous approach to scoping and selecting the literature including the comprehensive searching of several academic and grey literature databases and sources
- Nonetheless, the identification of relevant studies was possibly hindered by the lack of consistency and interchangeability of the terms 'integrated care and patient safety' and their use in the literature.
- While the review was able to identify the different types of patient safety approaches (scope) it was more challenging to describe their scale across care boundaries.

For peer review only

Introduction

Health and social care systems globally are facing the challenges of an ageing population with complex co-morbidities that require multi-faceted care provision to better manage their health and care needs. [1] Care provision for adults (particularly older and frail) with long term conditions can be fragmented, impacting the quality of care, with a greater potential for adverse outcomes. [2] The integration of health and social care systems and services is seen as integral to tackling these challenges. [3] The term 'integration' broadly represents a 'joining up' of traditional silos of care across (horizontal) and within (vertical) systems, organisations and services. Integrated care transcends longstanding professional, organisational and regulatory boundaries to provide more coordinated, collaborative and person-centred care. [4-6] Many high-income countries have taken steps to deliver integrated care through integrated care systems which involve the collaboration of sectors and care providers, bringing together primary and secondary healthcare, social care, community and mental health services and voluntary organisations. [7]

The World Health Organisation (WHO) defines patient safety as 'the absence of preventable harm to a patient during the process of health care and reduction of risk of unnecessary harm associated with health care to an acceptable minimum.'[8] Safer care, involves robust risk management – early identification, continuous monitoring and swift action to address risks.

Integrated care may contribute to improving the safety of care for people with long term conditions at different levels of the system, particularly by addressing the risks posed by multiple referrals, handovers and discharges that arise as the responsibility for care crosses care boundaries and involves different practitioners, teams, organisations and systems. [9] Integrated care provides health and care professionals with knowledge of an individual person's diverse care needs through shared information systems, enabling service providers to minimise gaps in treatment and care and address conflicts or duplication in care plans. This creates an environment that supports safety practices including proactive care through timely identification and enhanced management of risk.

For peer review only - http://bmjopen.bmj.com/site/about/guidelines.xhtml

For peer review only

Furthermore, person centred care (a key tenet of integrated care) supports people to manage their own care more effectively, ensuring care is tailored to their specific needs and hence, could be regarded as a precursor to safer care as individuals are more aware of the risks of their care activities and their role in mitigating these. [10] With this in mind strengthening some of the key structural and relational aspects of integrated care [11] such as data sharing, communication, partnership working, care coordination and person-centred care may improve patient safety by enhancing risk management. Patient safety does not necessarily arise from integrated care but from the opportunity for enhanced risk management.

Integrated care initiatives encourage care provision closer to home i.e. in the community setting which are safer environments compared to hospitals. [12]However, adverse events in this setting are responsible for between 8-12% of hospital admissions, around a half of which are thought to be preventable. [13 14] Harm in this setting may result from medication mismanagement, poor infection control, failure to ensure safe mobilisation or a lack of prevention of and care for pressure sores/ulcers. [15] Studies have also highlighted that the 'spaces' between care settings are prone to problems of communication and coordination which affect the safety of care as patients transition across care boundaries and receive care from different professionals. [16 17]

A handful of conceptual models of integrated care have been described in the literature. One such model suggests integrated care has a supporting role at all levels of the system, micro (services, meso (organisations) and macro (systems). [18] Recent approaches to better management, assurance and improvement of patient safety have advocated for developing safety governance through a whole systems approach addressing safety issues at the all levels of a care system. [19]

Initial scoping of the literature on how safety is improved for adults with long term conditions in integrated care revealed a limited evidence based on the scale and scope of safety approaches implemented in integrated care systems. As the relevant literature to address this issue was likely to

For peer review only - http://bmjopen.bmj.com/site/about/guidelines.xhtml

be extensive, complex and potentially heterogeneous, a scoping review was selected over a systematic review. [20] This scoping review aims to address this gap by establishing the approaches employed to improve patient safety in integrated care for community dwelling adults with long term conditions and how these are evaluated. Such assessments will gauge current key priorities for safety within integrated care and the types of patients safety measures that may be useful for ongoing monitoring of safety at different levels of a system.

Methods

This scoping review was conducted in accordance with the PRISMA extension for scoping reviews (PRISMA-ScR) guidance.[21] A protocol was developed but not published. We used the Arksey and O'Malley's framework in undertaking this scoping review; (i) identifying the research question; (ii) identifying relevant studies; (iii) selecting studies; (iv) charting the data; (v) collating, summarising and reporting the results.

Stage 1: Identifying the research questions

The scoping review explored the breadth of literature to answer the research question, 'What type of integrated care approaches exist at different system levels to improve patient safety for community-dwelling adults with long term conditions and how have they been assessed?'

Stage 2: Identifying relevant studies

Free text search terms were developed based on our research question. The search term strategy (supplementary file 1) was broadly based on three blocks of terms; 1) population (elderly/vulnerable adults/long-term conditions), 2) approach (associated with integrated care/multidisciplinary working), and c) outcome (related to patient safety/risk reduction/preventable harm).

For peer review only - http://bmjopen.bmj.com/site/about/guidelines.xhtml

A comprehensive search strategy was employed to include both the peer-reviewed and grey literature. Seven electronic databases were searched including MEDLINE, Social Policy and Practice, Web of Science, CINAHL, Google Scholar, Proquest and OpenGrey. These were supplemented by forward and ancestry citation searches using reference lists of several relevant studies.

Stage 3: Selecting studies

Study selection was based on the inclusion and exclusion criteria listed in table 1.

Table 1: Overview of exclusion and inclusion criteria

**Inclusion criteria**	
Organisational setting	All types i.e. acute, primary, community, mental health and social care
Population	Adults over 18 with long term conditions
Approach	Involved a team or group of professionals representing different sectors e.g. acute and social care, often referred to as a multidisciplinary team (MDT)
Outcome	Associated with patient safety as defined by the WHO
Study design	Primary and/or secondary data (research studies, quality improvement project reports, service evaluations and reviews)
Time interval	2000-2021 - We selected 2000 as a start date because it coincides with a proliferation in safety-related research stimulated by the publication of the seminal report; To Err is Human: Building a Safer Health System. (21)
	Studies published in English only
**Exclusion criteria**	
Organisational setting	Transitions of care within the same sector e.g. hospital only<br>Studies in which it is challenging to discern whether staff in an MDT were employed by different providers and therefore were part of an integrated care team
Type of study	We also excluded commentaries and editorials.

The first reviewer (ML) undertook initial searches and based on information in the title and abstract selected those meeting criteria for full text review. At this stage, any studies that the first reviewer was uncertain about in terms of meeting eligibility criteria were checked by the second reviewer. At the full text review stage both the first and second reviewer (SW) independently assessed full texts against the eligibility criteria and any discrepancies were discussed.

Stage 4: Charting the data

The first reviewer extracted and tabulated the data from the included studies in Microsoft Excel. Details of the studies extracted included the overall aim, population, study design, level of system,

For peer review only - http://bmjopen.bmj.com/site/about/guidelines.xhtml

139 integrated care approach, setting and approach to measuring safety. The second reviewer reviewed
140 and verified the data extraction table. Descriptions of the levels of the system i.e. micro, meso and
141 macro and the approach to measuring safety (outcome, process and qualitative) [22] are outlined in
142 box 1. The nomenclature for different levels of a system (micro, meso and macro) can be ascribed to
143 both integrated care and patient safety system governance. [19 23] The Donebedian framework for
144 measuring quality in health care applies to identifying measures for safety i.e. outcome and process,
145 in the case of this review. [24] We have also added a further measure, 'qualitative' to capture
146 perceptions of study participants regarding service improvements associated with patient safety
147 through implementation of approaches.

For peer review only - http://bmjopen.bmj.com/site/about/guidelines.xhtml

Box 1: Description of the different levels of a care system

Levels of care system:

Micro-level: Typically comprises an approach at the patient level where multiple professionals from different types of organisations provide care commonly in the home setting but sometimes in primary care alone or in primary and secondary care if linked by a common pathway.

Meso level: Approaches at organisation level that involve multi-professionals in planning or designing local structures, care pathways and services that span two or more different types of organisations.

Macro level: System wide approaches that may involve several providers with a typical focus on strategy, infrastructure development such as shared care records and other wide-ranging cultural and educational initiatives. Approaches implemented over a wider a geography within a country e.g. region, county, state etc.

Approaches to measuring safety:

Outcome measures: Indicate the impact of a health care service or approach on the patient's health status.

Process measures: Practices delivered in order to achieve the desired outcome of an approach.

Qualitative outcomes: Perspectives of study participants on the implementation and/or impact of an approach (primarily from qualitative studies only).

Quality assessment

Due to the heterogeneity of the studies in terms of their methodological design, settings in which an approach was implemented as well the design and scope of the approach itself, and in line with similar scoping reviews, formal quality assessment was not undertaken. [25 26]

Patient and Public Involvement

No patients or members of the public were directly involved in this work. However, this study is part of a wider programme of research for which there is a research advisory group whose membership includes patients/public representatives. In the conceptualisation phase of the study, the research advisory group provided advice and guidance on the study's aims and purpose.

For peer review only - http://bmjopen.bmj.com/site/about/guidelines.xhtml

Results

Stage five: collation, summarising and reporting

The original searches yielded 2957 records. After removing duplicate records and screening of titles and abstracts, 86 were allocated for full text review. After application of eligibility criteria, 24 studies were included in the review. The flow of studies from initial identification to final inclusion are presented in figure 1.

Figure 1: Flowchart of study selection

General characteristics of included studies

All included studies were published between 2007 and 2019. We identified one review [15] and 23 empirical studies. A breakdown of the study characteristics is provided in the supplementary table. In terms of geographical distribution, empirical studies were undertaken in North America [27-32], Europe [33-45] Asia [46 47] and Australia [48]. All studies involved adult participants described as elderly, older adults or adults over the age of 65 in the main, although four studies also included other adults aged over 18. In all studies, the population in receipt of an approach were reported to have long-term, chronic physical or mental health conditions. However, in a few of these studies, a health condition(s) was not specified and instead participants subject to the approach were described as frail or vulnerable. Empirical studies comprised the majority of study types and included randomised control trials (RCT), [37 38 40 42 43 48 49] qualitative studies, [35 36 39 50] mixed methods studies, [27 34] retrospective analysis of medical records, [30 44] a cohort study, [47] a cross-sectional study, [33] an observational study,[36] a systematic review [15] and action research. [51] Service evaluations [28 31 32] and quality improvement (QI) studies [29 46] were also among the list of included studies.

Types of risks addressed

For peer review only - http://bmjopen.bmj.com/site/about/guidelines.xhtml

Of the 24 included studies, 11 involved approaches designed to reduce falls risk, [27 28 32 34 37 38 42 47-49 51] and ten studies were broadly associated with assuring medication safety through reconciliation and review, surveillance and monitoring [29 33 35 36 39 40 43 44 46 50]. Three studies undertook a series of approaches to reduce adverse events more broadly such as identifying medications that increase the risk of a fall in older people and an assessment of an approach designed to reduce the rate of hip fracture. [30 31] The systematic review included studies that involved approaches to reduce adverse events including adverse drug reactions. [15]

Integrated care approaches

Setting

The studies included a range of settings. A total of 13 studies described approaches implemented in a single setting involving a group or team of professionals from different sectors; the home setting [31 34 35 38 42 43 47 48] and the remainder in hospitals, [30 32 36] community health centres, [29] and primary care. [50] In the remaining studies, a care pathway was established either across several settings [15 28 46 51] or just two settings; between the hospital and home setting [37 39 49] or hospital and primary care.[27 33 40 44]

Level of care system

Various approaches were implemented at different levels of the care system; micro, meso and macro. Ten studies were at the micro level, [30 34 36-38 42 43 47-49] five at the meso level [15 31 32 40 50] and nine at the macro level [27-29 33 35 39 44 46 51]. Broadly, these approaches can be categorised into four types; i) MDT approach to care delivery in community (home) or primary care setting, ii) care pathway, iii) educational initiative and iv) integrated digital record (accessible to different care providers).

<u>Micro level</u>

For peer review only - http://bmjopen.bmj.com/site/about/guidelines.xhtml

At the micro level, approaches were characterised by the delivery of person-centred coordinated care for adults with long term conditions involving the home (community) setting or primary care setting alone or as part of a care pathway with secondary care settings. Care was provided by a range of health and care professionals working together. Three studies outlined multidisciplinary approaches to prevent and reduce falls, primarily in the home setting such as exercise therapy, home based assessments for hazards with accompanying modifications, vision assessment, home medication review and a 24 hour call service staffed by a home intervention team.[34 38 48]

A further five studies described approaches implemented across different parts of a care pathway to reduce falls, although *Wallace et al* focussed on the development of a multi-disciplinary care pathway following a hip fracture.[30 37 42 47 49] Approaches comprised multi-disciplinary outpatient clinics (often located in hospitals) where patients were assessed and monitored, with further follow-up care provided by community services staff in the home setting.[37 42 47 49] Follow up care included assistance with daily living, medication review, balance and cognition,[37 47 49] as well as promotion of patient/user independence through home hazard (home environment risk) assessments,[42 47 49] exercise to improve mobility and strength,[37 42 47] and patient education on use of assisted devices to support individuals to navigate their home environment.[37]

Three studies involved medicines reviews to reduce the risk of adverse drug events (ADE) including the reduction of falls.[31 43 50]

<u>Meso-level</u>

At the meso-level approaches were mainly focused on multi-professional management to plan and design services across at least two different types of organisation, primarily hospitals and community care. Approaches tackled areas with overarching safety risks such as poor communication or dispersed leadership. All five studies used different approaches.

For peer review only - http://bmjopen.bmj.com/site/about/guidelines.xhtml

226 Three studies aimed to improve medication safety for older people. One study employed an Integrated Medicines Management (IMM) model (spanning hospitals and primary care) which comprised medicines reconciliation and review, patient counselling on medicines usage during hospital stay and greater provision of information and communication with primary care at discharge and post-discharge. [40] Another study described an approach in primary care involving managers, pharmacists and nurses who engaged in self-assessment, peer review, feedback and agreement for change to improve medicines safety management. [50] *Willis et al* described an MDT approach involving medication reviews, home fall risk assessments and blood pressure monitoring.[31]

234 A further study, outlined a multidisciplinary falls committee (within a newly created ICS) whose role was to provide leadership, direction and coordination to frontline staff promoting new evidence based practices and quality improvement support to prevent falls. [32] The systematic review described approaches such as education and training, organisational/culture interventions (e.g. discharge protocol and planning, medication reconciliation etc.), or patient/family orientated approaches concerning awareness and empowerment for patients. [15]

240 <u>Macro level</u>

241 At the macro level, system-wide approaches centred on managing medication safety and reducing falls risk such as key infrastructure developments - integrated care records, training and education initiatives spanning several organisations within a system and creating care pathways involving multiple organisations. Approaches were generally implemented across a wider geography e.g. county, state or region.

246 Three studies described digital approaches to medication safety monitoring such as a digital database for patients transitioning from secondary to primary care with indicators to alert practitioners to potential ADE [33] and a digital measurement tool designed to assess transitional safety incidents affecting older people including ADE. [44] A further study described how an e-message

For peer review only - http://bmjopen.bmj.com/site/about/guidelines.xhtml

system for nurses in hospital and community settings could support information exchange, minimising ADE. [39]

Two studies described educational approaches comprising training of inter-professional teams (including social workers) from a range of settings on developing strategies for preventing falls in accordance with national evidence-based guidelines. [27 28]

The remaining studies described multi-component models such as quality improvement collaborative approaches, or other models comprising multi-disciplinary working, care coordination and strategies to strengthen leadership to promote patient safety and reduce hospital admission or readmission. The Quality improvement collaborative aimed to reduce the risk of ADE and involved MDTs with professionals from primary and acute care who adopted a change package involving clinical pharmacy services [29] and a quality improvement approach based on an adaptation of the Institute of Healthcare Improvement Trigger Tool method. [46]

Profile of professionals involved in the approaches

Of the 23 empirical studies, the majority included a multidisciplinary approach to care provision involving different health professional groups. Four of the approaches also included social workers as part of the team. [27 28 30 32] In eight studies, the approaches included health and social care professionals such as nurses, paramedics, managers, social workers, and allied health professionals; physiotherapists and occupational therapists, pharmacists and optometrists but doctors were not involved. [28 31 32 35-37 39] Many of these studies involved approaches implemented in the community (home setting) or where the home was included as a part of a care pathway.

Measures for assessing impact of the approach

There was heterogeneity in the approaches to measuring safety. We categorised measures as process, outcome or qualitative.

For peer review only - http://bmjopen.bmj.com/site/about/guidelines.xhtml

273 Two studies included process measures alone. The first involved the monitoring and surveillance of medication safety including the proportion of older patients prescribed falls risk medication, and the proportion of medicines prescribed with have a high potential for major drug interaction. [31] The second study identified measures associated with educational outcomes of health professionals participating in a falls training workshop including subsequent changes to practice. This included workshop participants self-reporting their undertaking of assessments of older patients at higher risk of falls and screening of falls rates. [28]

280 In 11 studies, outcome measures alone were mentioned. Four of these studies stated falls incidence during follow up over a period of 6-12 months [37 38 49] as a key measure. [51] In three further studies, falls and falls related injuries such as hip fracture, and hospital length of stay were key outcomes [30 47 48]. In four studies, ADE were stated as outcome measures including preventable ADE or potentially preventable ADE, [33 46] proportion of ADE detected in a specified population [29], and ADEs leading to unplanned hospital admission. [44]

286 Six studies included both process and outcome measures. Process measures associated with falls included adherence to falls prevention strategies, proportion of falls risk assessments undertaken, documenting of falls and medical history risk of falling, and level of independence in activities of daily living. [27 40 42] Outcome measures in these studies included a decrease in falls rates, time between the first and second falls, falls related subsequent emergency department visits and hospitalisation.

292 Process measures associated with medication safety comprised medication appropriateness and use of harmful medications. [32 43] Outcome measures in these studies were medication related readmissions, rate of hip fractures and clinically significant drug interactions. The systematic review described a range of process and outcome measures across the included studies, all associated with adverse events including ADE. [15]

For peer review only - http://bmjopen.bmj.com/site/about/guidelines.xhtml

The remaining studies described qualitative outcomes for the integrative approaches i.e. based on the perceptions of study participants. These included assessing approaches designed to prevent falls such as the perceived usefulness of a MDT home-based fall prevention programme. [34] A further four studies described qualitative outcomes in terms of assessing the quality of medication safety management practices. These included the extent of professional learning experiences to support medicines management, [35] the quality of service delivery of a domiciliary hospitalisation unit and its impact on assuring drug safety, [50] the usefulness of a self-assessment tool in identifying areas of improvement for medication safety [36] and the ability of an E-message system to support information exchange to assure medication safety. [39]

Discussion

This scoping review brings together the published literature on approaches to improve patient safety in integrated care for community-dwelling adults with long term conditions and demonstrates the range of approaches employed in safety improvement strategies for care across boundaries within several countries. The approached identified in the included studies focus primarily on two common patient safety targets for harm, falls and ADE through falls prevention and medication safety programmes spanning micro, meso and macro levels of a health and care system. Just over a third of the included studies involved the implementation of system-wide approaches (macro level) such as digital shared care records, training/educational initiatives and the strengthening of care pathways between providers. A higher proportion (40%) of studies described micro-level approaches, primarily MDT delivering care to older people in the home setting alone or as part of a care pathway involving hospitals and primary care. The review also highlighted that the evaluation of approaches indicated a perspective of safety focused primarily on measurement - changes in rates of adverse events or contributory factors at individual patient level.

For peer review only - http://bmjopen.bmj.com/site/about/guidelines.xhtml

A key strength of this study was the highlighting of how key components of integrated care can also improve safety across care boundaries particularly in reducing falls and medication safety management. Instituting integrated care practices at the different levels of a care system e.g. digital shared care records (macro), strengthening of care pathways (macro, meso and micro) and promoting MDT working (meso and micro) will strengthen safety approaches and reduce the risk of harm across an ICS. The study findings also highlight how in some cases MDT working, particularly in community care does not directly involve doctors. This reflects an increasing trend in some Western European countries where integrated community care teams (without doctors) are primarily responsible for care provision in the home setting for older and/or frail patients with complex care needs, partly promulgated by the extension of roles of community care professionals and a shortage of primary care doctors. [52 53] The study also identified a range of measures to assess patient safety across systems suggesting that ICS will need to develop a range of indicators to capture the variation in performance of key processes that lead to desired safety outcomes. Another strength of the study was the rigorous approach to scoping and selecting the literature including the comprehensive searching of several academic and grey literature databases and sources. In comparison to the existing literature on this topic, our study builds upon previous work on safety in care transitions but extends beyond the spaces and pathways between organisations at the micro level to consider system-wide (meso and macro) approaches to improving safety.

The study was not without limitations. Articulating a precise definition for patient safety was challenging, as, in several studies, the term was associated with preventing hospitalisation. Hence, we selected the WHO definition which is broad ranging and encompasses the notion of errors of commission and omission. Nonetheless, the identification of relevant studies was hindered by the lack of consistency and interchangeability of the terms ‘integrated care and patient safety’ and their use in the literature. We may have screened for word choice rather than presence or absence of a similar term or its description. Despite including a range of search terms associated with patient safety some studies may have been missed. Our search strategy did not include social care as a

For peer review only - http://bmjopen.bmj.com/site/about/guidelines.xhtml

specific search term, although integrated care including care coordination and multidisciplinary working increasingly involves social care professionals. We also did not search for 'person-centred care' or 'self-care' both of which are potentially effective risk management strategies. This may explain why we were unable to identify any studies reporting the experience of patients/carers. The nature of our search didn't lead to papers that talked about implementation of structures or their measurement and hence we only included process and outcome measures. While the review was able to identify the different types of patient safety approaches (scope) it was more challenging to describe their scale across care boundaries.

Integrated care initiatives identified in this study involved the design of a range of new structures and processes to support the delivery of safer care, although further work is required to establish their effectiveness in improving patient safety. Integrated digital care records have been cited as a key enabler for structural integration in ICS as they promote sharing of information thus minimising the risk of care-related information being overlooked as people (particularly older and more frail) move along a care pathway. [54] Training and educational initiatives delivered to inter-professional teams are also considered to be a means of improving the quality of integrated care delivery, supporting the development of social and working relationships thereby promoting partnership working. [55] At all levels, several approaches were implemented to strengthen the care pathway on discharge from hospital and provide tailored care in the home setting to reduce the risk of adverse events. These initiatives had several goals associated with integration, to deliver care closer to home, reduce duplication, minimise the risk of hospital admission and provide personalised care. [3] Each of these goals are notable processes of integration which, if successfully implemented, could also improve patient safety outcomes. These focused approaches do not negate the need for further efforts to develop patient safety within ICS including safety governance at all levels, system-wide mechanisms to identify, review and learn from safety incidents, enhanced capacity and capability for measurement and improvement, and creating learning cultures that promote openness and transparency. [19 56]

For peer review only - http://bmjopen.bmj.com/site/about/guidelines.xhtml

The levels of the system (micro, meso and macro) approach to identifying integrated care and patient safety approaches presented in this study provides a useful means of understanding how organisations can implement change, improvement and innovation across an integrated care system. At the macro level, the implementation of integrated care records to collate information and support communication alongside the promotion of a learning culture supported by inter-professional training and education is required. At the meso level, the focus would be on organisations jointly planning and designing multi-disciplinary approaches to develop seamless care pathways, minimising the risk of harm as people transition between providers. At the micro-level, a range of multi-disciplinary working practices to improve safety could be deployed from near real time safety monitoring within 'community huddles' to protected time sessions to compare and reflect on performance across teams promoting longer term changes. [57] These approaches are potential solutions to the fragmentation of health and care systems (which continues despite efforts to integrate care) and will be integral to improving quality and safety outcomes for increasing populations of people with long term conditions as they receive care closer to home. [19]

The studies included in this review evaluated falls reduction and medicines safety approaches using clinical process and outcome measures with several authors looking at associations between these outcomes and wider health system outcomes e.g. hospital (re) admissions and length of stay. System outcome measures have been widely used as performance indicators to assess the impact of integrated care. [3 58] While structural or relational measures of integration such as collaborative and co-terminus working [52 59 60] or the aforementioned system outcomes dominate the discourse of the effects of integrated care, less attention has been paid to measures related to patient or carer experience. [61] None of the studies in this review collected data on the experience of patients and carers alongside clinical outcomes. The understanding of experiences of patients in receipt of services may support service improvement to deliver better quality care. [62 63]

For peer review only

For peer review only - http://bmjopen.bmj.com/site/about/guidelines.xhtml

A wide range of process and outcome measures were used across the studies predominantly to capture patient harm and contributory factors. Expanding measurement to incorporate the capture of the variation in performance of key processes that lead to desired outcomes, would not only identify the most effective approaches for improving patient safety but also open up a wider range of options to support building resilience across ICS. [64] A standardised set of safety indicators would support comparisons between different integrated care approaches to safety.

Safety approaches in this review were mainly focused on medication safety management and falls prevention which is unsurprising as both account for a notable proportion of unplanned hospital admissions. In England, in 2018 falls accounted for a total of 220,160 hospital admissions in those aged over 65. A study of medication errors in the UK estimated that ADE accounted for 27,362 hospital admissions annually, relating to 136,811 bed days at a cost of £83.7 million to the NHS. [65 66] While falls and medication safety are justifiably key safety priorities for integrated care, there is scope to broaden the focus to other common safety issues. For example, a study in the Netherlands found that of 106 admissions to hospital in patients aged over 65 over a period of 12 months were due to an adverse event with 45% were due to procedural related infections, community acquired infection such as pneumonia with/without signs of sepsis and diabetes related hypoglycaemia. [66 67]

Future research should focus on the role of other patient safety targets (not just falls and medication related safety) such as healthcare associated infections, community acquired infections and pressure ulcers/sores which also increase the risk of harm for people with long term conditions especially in the community setting. Furthermore, research must establish the effectiveness of the structural and relational aspects of integrated care such as care coordination as well as the approaches identified in this review e.g. integrated care records and education and training, in terms of their impact on reducing the risk of harm across care pathways. This would provide the evidence for policymakers and system leaders to implement the necessary changes to support integration, safety governance and service improvement.

For peer review only

Conclusion

In order to maximise the potential for ICS to fulfil their promise of improving care for people with long term conditions, approaches such as integrated care records, allocating resources for training and education, greater efforts to form robust and risk-limited care pathways as well as support for MDT working, especially in the community setting will have to be key priorities for improving safety across systems. They also align to the goals of ICS by reducing fragmentation and enhance personalisation of care. Such a focus would ensure that integrated care reaches its full potential as an enabler of safety supporting risk management and personalisation of care for people with long term conditions.

Data availability statement

Data sharing not applicable as no datasets were generated and/or analysed for this study. All data relevant to the study are included in the article or uploaded as online supplemental information.

Ethics approval statement

No ethical approval was needed because data from previous published studies are being used.

Funding

This research is funded by the National Institute for Health Research (NIHR) Policy Research Programme, conducted through the Quality, Safety and Outcomes Policy Research Unit, PR-PRU-1217–20702. The views expressed are those of the author(s) and not necessarily those of the NIHR or the Department of Health and Social Care.

Competing interests

None.

For peer review only

For peer review only - http://bmjopen.bmj.com/site/about/guidelines.xhtml

Author Contributions

ML conceived and designed the study. All authors contributed to several aspects of the review process i.e. stages 1-5 described in the manuscript under the methods section. ML and HH identified the research question. ML developed the search strategy (with support from HH) and identified relevant studies. ML and SW were involved study selection, charting of the data from included studies and results collation, reporting and summarising of the data. ML drafted the manuscript. All authors approved the final version of the manuscript.

For peer review only

For peer review only - http://bmjopen.bmj.com/site/about/guidelines.xhtml

References

1. Armitage GD, Suter E, Oelke ND, et al. Health systems integration: state of the evidence. *Int J Integr Care* 2009;9(2):e82. doi: 10.5334/ijic.316 [published Online First: 2009/07/11]
2. Stange KC. The problem of fragmentation and the need for integrative solutions. *Annals of family medicine* 2009;7(2):100-03. doi: 10.1370/afm.971
3. Baxter S, Johnson M, Chambers D, et al. The effects of integrated care: a systematic review of UK and international evidence. *BMC health services research* 2018;18(1):350. doi: 10.1186/s12913-018-3161-3
4. Ham C, Dixon J, Chantler C. Clinically integrated systems: the future of NHS reform in England? *BMJ (Clinical research ed)* 2011;342:d905. doi: 10.1136/bmj.d905 [published Online First: 2011/03/30]
5. Waring J, Marshall F, Bishop S. Understanding the occupational and organizational boundaries to safe hospital discharge. *Journal of Health Services Research and Policy* 2015;20(1 Suppl):35-44. doi: 10.1177/1355819614552512 [published Online First: 2014/12/05]
6. Glasby J. If Integration Is the Answer, What Was the Question? What next for English Health and Social Care Partnerships? *Int J Integr Care* 2016;16(4):11. doi: 10.5334/ijic.2535 [published Online First: 2017/03/21]
7. Shaw S, Rosen R, Rumbold B. What is Integrated Care? London: Nuffield Trust, 2011:1-23.
8. WHO. Patient safety: World Health Organisation; 2021 [Available from: https://www.who.int/teams/integrated-health-services/patient-safety.
9. Waring J, Bishop S. Health States of Exception: unsafe non-care and the (inadvertent) production of 'bare life' in complex care transitions. *Sociology of Health and Illness* 2020;42(1):171-90. doi: 10.1111/1467-9566.12993 [published Online First: 2019/09/19]
10. Santana MJ, Manalili K, Jolley RJ, et al. How to practice person-centred care: A conceptual framework. *Health Expectations* 2018;21(2):429-40.
11. Lalani M, Bussu S, Marshall M. Understanding integrated care at the frontline using organisational learning theory: A participatory evaluation of multi-professional teams in East London. *Social Science & Medicine* 2020;262:113254. doi: https://doi.org/10.1016/j.socscimed.2020.113254
12. Hammond J, Speed E, Allen P, et al. Autonomy, accountability, and ambiguity in arm's-length meta-governance: the case of NHS England. *Public Management Review* 2019;21(8):1148-69. doi: 10.1080/14719037.2018.1544660
13. Illingworth J. Is the NHS getting safer? London, UK: The Health Foundation, 2015.
14. Vincent C, Burnett S, Carthey J. The measurement and monitoring of safety. London: The Health Foundation, 2013.
15. Laugaland K, Aase K, Barach P. Interventions to improve patient safety in transitional care--a review of the evidence. *Work (Reading, Mass)* 2012;41 Suppl 1:2915-24. doi: 10.3233/WOR-2012-0544-2915 [published Online First: 2012/02/10]
16. Aase K, Schibevaag L, Waring J. Crossing boundaries: quality in care transitions. Researching Quality in Care Transitions: Springer 2017:3-29.
17. King BJ, Gilmore-Bykovskyi AL, Roiland RA, et al. The consequences of poor communication during transitions from hospital to skilled nursing facility: a qualitative study. *Journal of the American Geriatrics Society* 2013;61(7):1095-102. doi: 10.1111/jgs.12328 [published Online First: 2013/06/03]
18. Valentijn PP, Schepman SM, Opheij W, et al. Understanding integrated care: a comprehensive conceptual framework based on the integrative functions of primary care. *Int J Integr Care* 2013;13:e010. doi: 10.5334/ijic.886 [published Online First: 2013/05/21]
19. Auraaen A, Saar K, Klazinga N. System governance towards improved patient safety: OECD, 2020.

For peer review only - http://bmjopen.bmj.com/site/about/guidelines.xhtml

20. Peters MD, Godfrey CM, Khalil H, et al. Guidance for conducting systematic scoping reviews. *International Journal of Evidence Based Healthcare* 2015;13(3):141-6. doi: 10.1097/xeb.0000000000000050 [published Online First: 2015/07/03]
21. Tricco AC, Lillie E, Zarin W, et al. PRISMA Extension for Scoping Reviews (PRISMA-ScR): Checklist and Explanation. *Annals of internal medicine* 2018;169(7):467-73. doi: 10.7326/m18-0850 [published Online First: 2018/09/05]
22. Improvement N. The how-to guide for measurement for improvement: NHS Improvement, 2020.
23. Curry N, Ham C. Clinical and service integration: The King's Fund, 2010.
24. Donabedian A. Evaluating the quality of medical care. *The Milbank memorial fund quarterly* 1966;44(3):166-206.
25. Duhn L, Godfrey C, Medves J. Scoping review of patients' attitudes about their role and behaviours to ensure safe care at the direct care level. *Health Expectations* 2020;23(5):979-91. doi: https://doi.org/10.1111/hex.13117
26. Cheraghi-Sohi S, Panagioti M, Daker-White G, et al. Patient safety in marginalised groups: a narrative scoping review. *International journal for equity in health* 2020;19(1):26-26. doi: 10.1186/s12939-019-1103-2
27. Eckstrom E, Neal MB, Cotrell V, et al. An Interprofessional Approach to Reducing the Risk of Falls Through Enhanced Collaborative Practice. *Journal of the American Geriatrics Society* 2016;64(8):1701-7. doi: 10.1111/jgs.14178 [published Online First: 2016/07/29]
28. McKenzie G, Lasater K, Delander GE, et al. Falls prevention education: Interprofessional training to enhance collaborative practice. *Gerontology & Geriatrics Education* 2017;38(2):232-43. doi: 10.1080/02701960.2015.1127809 [published Online First: 2016/02/18]
29. Robbins CM, Stillwell T, Johnson D, et al. Integrating patient safety and clinical pharmacy services into the care of a high-risk, ambulatory population: a collaborative approach. *Journal of patient safety* 2013;9(2):110-7. doi: 10.1097/PTS.0b013e318281b879 [published Online First: 2013/05/24]
30. Wallace R, Angus LDG, Munnangi S, et al. Improved outcomes following implementation of a multidisciplinary care pathway for elderly hip fractures. *Aging clinical and experimental research* 2019;31(2):273-78. doi: 10.1007/s40520-018-0952-7 [published Online First: 2018/04/25]
31. Willis JS, Hoy RH, Jenkins WD. In-home medication reviews: a novel approach to improving patient care through coordination of care. *Journal of community health* 2011;36(6):1027-31. doi: 10.1007/s10900-011-9405-3 [published Online First: 2011/04/19]
32. McQuaid-Bascon K, Royal M, Sinno M, et al. Evolving a multi-factorial, data driven, interprofessional approach to prevent falls and associated injuries during a system-level integration. *Journal of Interprofessional Education* 2018;12:8-12.
33. Akbarov A, Kontopantelis E, Sperrin M, et al. Primary Care Medication Safety Surveillance with Integrated Primary and Secondary Care Electronic Health Records: A Cross-Sectional Study. *Drug safety* 2015;38(7):671-82. doi: 10.1007/s40264-015-0304-x [published Online First: 2015/06/24]
34. Amacher AE, Nast I, Zindel B, et al. Experiences of general practitioners, home care nurses, physiotherapists and seniors involved in a multidisciplinary home-based fall prevention programme: a mixed method study. *BMC health services research* 2016;16 doi: 10.1186/s12913-016-1719-5
35. Bell HT, Granas AG, Enmarker I, et al. Nurses' and pharmacists' learning experiences from participating in interprofessional medication reviews for elderly in primary health care - a qualitative study. *BMC family practice* 2017;18(1):30. doi: 10.1186/s12875-017-0598-0 [published Online First: 2017/03/01]
36. Brito AM, Simoes AM, Alcobia A, et al. Optimising patient safety using pharmaceutical intervention in domiciliary hospitalization. *International journal of clinical pharmacy* 2017;39(5):980-84. doi: 10.1007/s11096-017-0512-9 [published Online First: 2017/08/26]

For peer review only - http://bmjopen.bmj.com/site/about/guidelines.xhtml

37. Di Monaco M, Vallero F, De Toma E, et al. A single home visit by an occupational therapist reduces the risk of falling after hip fracture in elderly women: A quasi-randomized controlled trial. *Journal of Rehabilitation Medicine* 2008;40(6):446-50. doi: 10.2340/16501977-0206
38. Di Pollina L, Guessous I, Petoud V, et al. Integrated care at home reduces unnecessary hospitalizations of community-dwelling frail older adults: a prospective controlled trial. *BMC geriatrics* 2017;17(1):53-53. doi: 10.1186/s12877-017-0449-9
39. Foged S, Norholm V, Andersen O, et al. Nurses' perspectives on how an e-message system supports cross-sectoral communication in relation to medication administration: A qualitative study. *Journal of clinical nursing* 2018;27(3-4):795-806. doi: 10.1111/jocn.14109 [published Online First: 2017/10/20]
40. Johansen JS, Havnes K, Halvorsen KH, et al. Interdisciplinary collaboration across secondary and primary care to improve medication safety in the elderly (IMMENSE study): study protocol for a randomised controlled trial. *BMJ open* 2018;8(1):e020106. doi: 10.1136/bmjopen-2017-020106 [published Online First: 2018/01/25]
41. Lenander C, Bondesson A, Midlov P, et al. Healthcare system intervention for safer use of medicines in elderly patients in primary care-a qualitative study of the participants' perceptions of self-assessment, peer review, feedback and agreement for change. *BMC family practice* 2015;16(1):117. doi: 10.1186/s12875-015-0334-6 [published Online First: 2015/09/05]
42. Peeters GM, de Vries OJ, Elders PJ, et al. Prevention of fall incidents in patients with a high risk of falling: design of a randomised controlled trial with an economic evaluation of the effect of multidisciplinary transmural care. *BMC geriatrics* 2007;7:15. doi: 10.1186/1471-2318-7-15 [published Online First: 2007/07/04]
43. Toivo T, Airaksinen M, Dimitrow M, et al. Enhanced coordination of care to reduce medication risks in older home care clients in primary care: a randomized controlled trial. *BMC geriatrics* 2019;19(1):332. doi: 10.1186/s12877-019-1353-2 [published Online First: 2019/11/30]
44. van Melle MA, Zwart DLM, Poldervaart JM, et al. Validity and reliability of a medical record review method identifying transitional patient safety incidents in merged primary and secondary care patients' records. *BMJ open* 2018;8(8):e018576. doi: 10.1136/bmjopen-2017-018576 [published Online First: 2018/08/15]
45. van Melle MA, van Stel HKF, Poldervaart JM, et al. Measurement tools and outcome measures used in transitional patient safety; a systematic review. *PloS one* 2018;13(6) doi: 10.1371/journal.pone.0197312
46. Khoo AL, Teng M, Lim BP, et al. A multicenter, multidisciplinary, high-alert medication collaborative to improve patient safety: the Singapore experience. *Joint Commission journal on quality and patient safety* 2013;39(5):205-12. doi: 10.1016/s1553-7250(13)39027-8 [published Online First: 2013/06/12]
47. Sze PC, Cheung WH, Lam PS, et al. The efficacy of a multidisciplinary falls prevention clinic with an extended step-down community program. *Archives of Physical Medicine and Rehabilitation* 2008;89(7):1329-34. doi: 10.1016/j.apmr.2007.11.044 [published Online First: 2008/07/01]
48. Mikolaizak AS, Lord SR, Tiedemann A, et al. A multidisciplinary intervention to prevent subsequent falls and health service use following fall-related paramedic care: a randomised controlled trial. *Age and ageing* 2016;46(2):200-07. doi: 10.1093/ageing/afw190
49. Hendriks MR, Bleijlevens MH, van Haastregt JC, et al. Lack of effectiveness of a multidisciplinary fall-prevention program in elderly people at risk: a randomized, controlled trial. *Journal of the American Geriatrics Society* 2008;56(8):1390-7. doi: 10.1111/j.1532-5415.2008.01803.x [published Online First: 2008/07/30]
50. Lenander C, Bondesson Å, Viberg N, et al. Effects of an intervention (SÄKLÄK) on prescription of potentially inappropriate medication in elderly patients. *Family practice* 2017;34(2):213-18. doi: 10.1093/fampra/cmw121

For peer review only - http://bmjopen.bmj.com/site/about/guidelines.xhtml

51. Noor H, et al. The development, implementation and evaluation of a transitional care programme to improve outcomes of frail older patients after hospitalisation. *Age and ageing* 2016;45(5):643-51.
52. Lalani M, Marshall M. Co-location, an enabler for service integration? Lessons from an evaluation of integrated community care teams in East London. *Health & social care in the community* 2022;30(2):e388-e96. doi: 10.1111/hsc.13211 [published Online First: 2020/11/06]
53. Drennan VM, Calestani M, Ross F, et al. Tackling the workforce crisis in district nursing: can the Dutch Buurtzorg model offer a solution and a better patient experience? A mixed methods case study. *BMJ open* 2018;8(6)
54. Murray R, Mckenna H. The Next Steps Towards Integrated Care: The King's Fund, 2020.
55. Jeffs L, Abramovich IA, Hayes C, et al. Implementing an interprofessional patient safety learning initiative: insights from participants, project leads and steering committee members. *BMJ quality & safety* 2013;22(11):923-30. doi: 10.1136/bmjqs-2012-001720 [published Online First: 2013/06/19]
56. OECD. Measuring Patient Safety: Opening the Black Box. France, 2018.
57. Montague J, Crosswaite K, Lamming L, et al. Sustaining the commitment to patient safety huddles: insights from eight acute hospital ward teams. *British Journal of Nursing* 2019;28(20):1316-24.
58. Kelly L, Harlock J, Peters M, et al. Measures for the integration of health and social care services for long-term health conditions: a systematic review of reviews. *BMC health services research* 2020;20(1):358. doi: 10.1186/s12913-020-05206-5
59. Kirst M, Im J, Burns T, et al. What works in implementation of integrated care programs for older adults with complex needs? A realist review. *International Journal for Quality in Health Care* 2017;29(5):612-24.
60. Bussu S, Marshall M. Organisational development to support integrated care in East London: the perspective of clinicians and social workers on the ground. *Journal of health organization and management* 2020;34(5):603-19. doi: 10.1108/jhom-10-2019-0294 [published Online First: 2020/07/19]
61. Crocker H, Kelly L, Harlock J, et al. Measuring the benefits of the integration of health and social care: qualitative interviews with professional stakeholders and patient representatives. *BMC health services research* 2020;20(1):515. doi: 10.1186/s12913-020-05374-4
62. Robert G, Donetto S. Whose data is it anyway? Patient experience and service improvement. 2020;25(3):139-41.
63. Thorstensen-Woll C. Understanding integration: how to listen to and learn from people and communities The King's Fund, 2021.
64. Smith KM, Valenta AL. Safety I to Safety II: A Paradigm Shift or More Work as Imagined?: Comment on" False Dawns and New Horizons in Patient Safety Research and Practice". *International Journal of Health Policy and Management* 2018;7(7):671.
65. England PH. Falls: Applying All Our Health: Public Health England, 2020.
66. Elliott R, Camacho E, Campbell F, et al. Prevalence and economic burden of medication errors in the NHS in England. 2018
67. Magdelijns FJH, van Avesaath REM, Pijpers E, et al. Health-care-related adverse events leading to admission in older individuals: incidence, predictive factors and consequences. *European journal of public health* 2016;26(5):743-48. doi: 10.1093/eurpub/ckw021

For peer review only - http://bmjopen.bmj.com/site/about/guidelines.xhtml

For peer review only

For peer review only - http://bmjopen.bmj.com/site/about/guidelines.xhtml

Figure 1: Flowchart of study selection

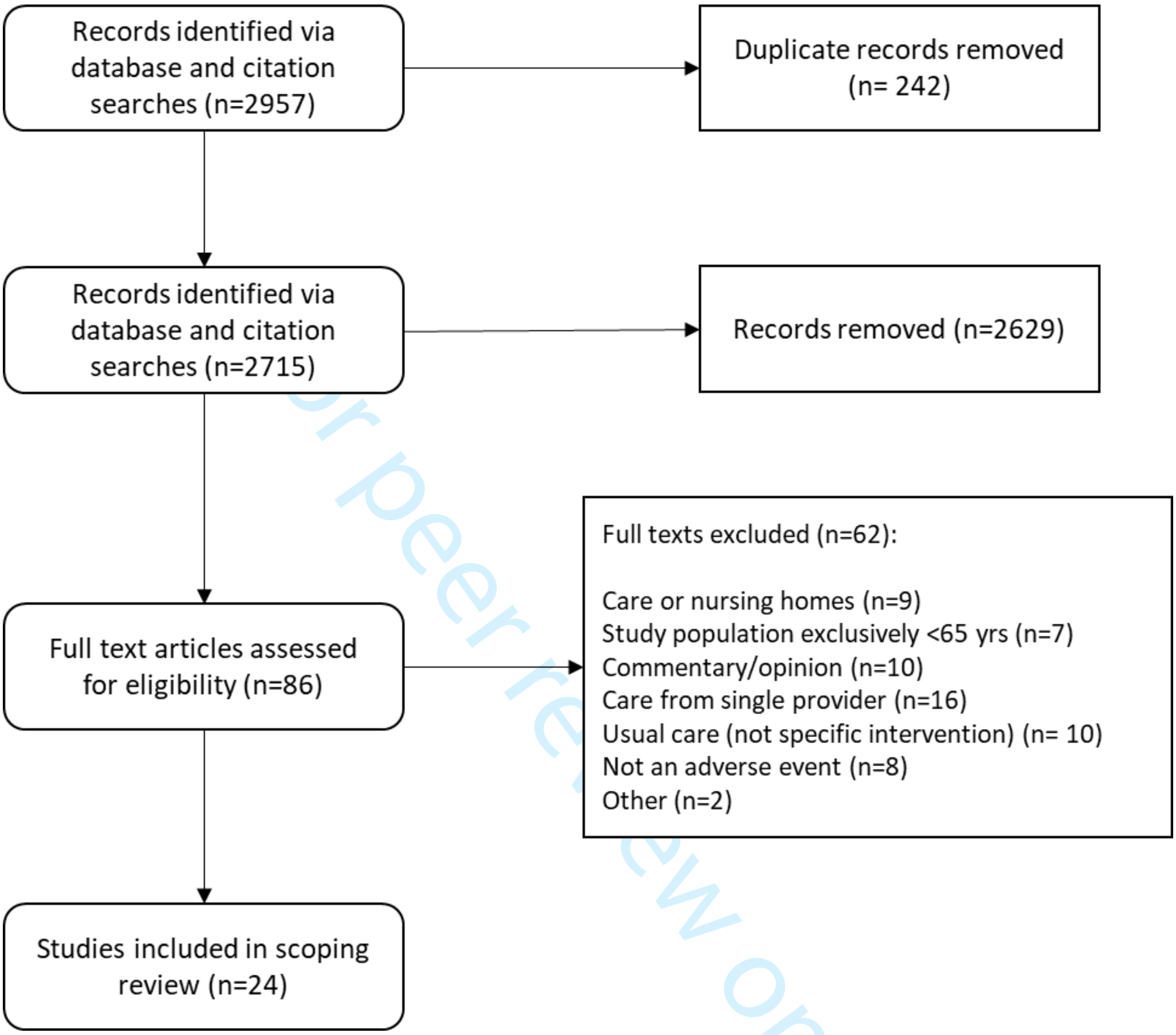

Additional File 1 – search strategy

Search terms (including MeSH) used were:

elderly (elder*) OR older OR older adults OR geriatric OR aged

vulnerable adults

mental health OR disorder

learning disabilities (*disability)

(-term OR long term) conditions

complex care

chronic conditions

multi-morbidity

AND

integrated care

care pathways

multidisciplinary OR multi-disciplinary

multiprofessional OR multi-professional

interprofessional OR inter-professional

transitional (W#) care (transitio*)

care (W#) coordination

AND

patient safety

preventable (w#) harm

risk reduction

adverse events

For peer review only

Supplementary table: Characteristics and description of included studies

Author and year	Country	Aim	Population	Study design	Integrated care approach				Approaches to measuring safety
					Type of approach	Description of approach	Setting	Level	
**Akbarov 2015 (36)**	England	Investigate the feasibility of wide ranging medication safety surveillance using a linked database	Adults with prescribing hazards	Cross-sectional study	Integrated digital record	Linked records of patients served by one hospital system and primary care centres - prevalence of 22 medication safety indicators, investigation of associations with patient and practice characteristics, and variation between primary care practices	Hospitals and primary care centres	Macro	Outcome measure: Proportion of patients with at least one medication safety hazard
**Amacher 2016 (37)**	Switzerland	Explore the perceived benefits and barriers of an evidence-based, home-based pilot falls prevention programme	Elderly	Mixed methods	MDT approach to care delivery in community (home) or primary care setting	Multi-factorial falls prevention programme (FPP)	Community (home)	Micro	Qualitative outcome: Usefulness of MDT home-based fall prevention programme
**Bell 2017 (38)**	Norway	Investigate nurses' and pharmacists' perceived learning experience after participating in an Integrated Medicines Management Review in primary health care	Elderly	Qualitative study	MDT approach to care delivery in community (home) or primary care setting	Integrated Medicines Management model – centred on inter-professional medication reviews	Community (home based services and nursing homes)	Macro	Qualitative outcome: Extent of professional learning experiences to support medicines management

For peer review only

**Brito 2017 (39)**	Portugal	Managing medication, in a patient-centred process and multidisciplinary context, by gathering information about the drug therapy of patients before admission, during hospitalization and after hospital discharge	Elderly	Observation study	Care pathway	Pharmacist working within domiciliary hospitalisation unit (DHU) alongside a multi-disciplinary team – medication reconciliation and review	Community (home) – domiciliary hospitalisatio n	Micro	Qualitative outcome: Perceived quality of DHU delivery and its impact on drug safety
**Di Monaco 2008 (40)**	Italy	Assessment of the effectiveness of a follow up home visit by an occupational therapist after a fall. MDT prevention intervention to reduce falls risk after hip fracture in elderly women	60 years or older	Quasi-RCT	Care pathway	MDT approach to prevent falls; exercise, advice and training on use of assistive devices, and activities of daily living	Hospital and community (home)	Micro	Outcome measure: Occurrence of falls in six months post-discharge from hospital
**Di Pollina 2017 (41)**	Switzerland	Testing the efficacy of providing integrated care at home to reduce unnecessary hospitalisations, emergency room visits, institutionalisation, and mortality in community	Frail and dependent people over 60 years	Prospective RCT	MDT approach to care delivery in community (home) or primary care setting	Integrated in-home geriatric assessments, long-term coordinated follow-up, and availability of a round-the-clock geriatric call service	Community (home)	Micro	Outcome measure: Falls over 3 years as a secondary outcome (stated as reason for hospitalisation)

For peer review only

		dwelling frail older adults							
**Eckstrom 2016 (30)**	US	Establishing changes in team behaviours after the intervention communicate and enhance adherence to Geriatrics Society Falls Prevention Guidelines by inter-professional teams in practice) and highlight lessons learned during the practice change process	Elderly patients	Mixed methods	Educational approach	Falls prevention strategy training workshops to clinical inter-professional teams – content based on guidelines developed by American Geriatric Society and British Geriatric Society	Hospitals and community or primary care clinics	Macro	Process measure: Adherence to falls prevention strategies<br><br>Outcome measure: Rate of falls, emergency department visits for falls, and hospitalisations for falls over a three month period
**Foged 2018 (42)**	Denmark	Describe nurse perspectives on how an e-message system supports communication on medication administration between hospital and home care nurses	Elderly	Qualitative	Integrated digital record	E-message system - admission report, care plan, discharge report, discharge notification and a message platform to bolster communication between hospital nurses and nurses providing home-based care	Hospital and community (home)	Macro	Qualitative outcome: The ability of an E-message system to support information exchange to assure medication safety
**Hendriks 2008 (52)**	Netherlands	Assess whether a MDT fall-prevention program was more effective than standard care in preventing new falls and functional decline in elderly community-dwelling people	Adults, 65 years and over	RCT	Care pathway	MDT in hospital providing a falls risk assessment, with referral to GP depending on risk and occupational therapy visits for a short period at home	Hospital and community (home)	Micro	Outcome, Primary measure: number of falls experienced in 12 month follow up period

For peer review only

		who attended an emergency department after a fall							
**Hiem 2016 (54)**	Netherlands	Decrease the risk of functional decline and loss of independence after hospitalisation in frail older patients	Elderly patients	Action research	Care pathway	Various approaches across primary and acute care to support frail patients. Approaches address (i) improved risk management; (ii) delivery of integrated, function-oriented care; (iii) specific geriatric approaches; and (iv) optimisation of transfers	Primary care, nursing home, rehabilitation centres and hospitals	Macro	Outcome: Incidence of adverse outcomes including falls in the last six months
**Johansen 2018 (43)**	Norway	Explore the effects of the intervention on healthcare use, health-related quality of life and medication appropriateness in elderly patients	70 yrs and over	Protocol RCT	Care pathway	Integrated medicines management model adapted to improve both medication safety in hospital and communication across primary and secondary care. Medication reconciliation (admission), medication review (during hospital stay), patient counselling (medicines use), medication list with explanations in discharge summary and post-discharge phone calls to primary care	Secondary and primary care	Meso	Secondary process measure: medication appropriateness, medication changes,<br><br>Outcome measure: hip fracture rate, medication-related readmissions

For peer review only

**Khoo 2013 (49)**	Singapore	Reduce adverse drug events resulting from high-alert medication	Adults on 'high risk of adverse events' medications	QI case study	Care pathway	Multicentre multidisciplinary collaborative focused on improving safe use of high-alert medication. QI approach with tracking of improvement conducted with an adapted IHI Trigger Tool method	Primary and acute care institutions	Macro	Outcome measure: Preventable or potentially preventable adverse drug events
**Laugaland 2012 (16)**	Global	Identify and evaluate the effects of the interventions in terms of effectiveness and efficiency of care processes in the context of effects of discharge interventions on patient safety	Patients 65 years and over	Review	Care pathway	Several studies that employed MDT approaches to transitional care to improve patient safety	Hospital, community and primary care	Meso	Process and outcome measures associated with adverse events
**Lenander 2015 (53)**	Sweden	Evaluate participants' perceptions of the SÄKLÄK project, which aims to enhance medication safety, especially for elderly patients, in primary care	Elderly	Qualitative study	MDT approach to care delivery in community (home) or primary care setting	SÄKLÄK intervention model involves a multidisciplinary group with self-assessment, peer review, feedback and agreement for change	Primary care	Meso	Qualitative outcome: Usefulness of self-assessment tool in identifying areas of improvement for medication safety

For peer review only

**Mckenzie 2017 (31)**	US	Describe an inter-professional team-based training approach to falls prevention for older adults and the related educational outcomes	Older adults	Service evaluation	Educational approach	Project that involves an inter-professional teaching team to support practice teams to reduce falls in older adults via implementation of evidence-based practice guidelines	Ambulatory, long term care, hospital, and home health practice	Macro	Process measure: Associated with educational outcomes resulting from falls training workshops; knowledge, learner confidence, intention for collaborative practice change and change to practice (falls screening rates and falls risk assessments)
**McQuaid-Bascon 2018 (35)**	Canada	An overview of a recently integrated health care system's experience in evolving their multi-component inter –professional approach to falls prevention	Older patients susceptible to falls	Service evaluation	Care pathway	Establishment of multidisciplinary falls committee to provide leadership, direction and coordination in the inter-professional advancement of quality improvement, evidence-informed practices and research associated with falls prevention and injury minimisation	Hospital	Meso	Process measure: i) sustain falls risk assessment and falls communication bundle across all areas; ii) monitor and report falls assessment rates and bundle implementation on inpatient areas; and iii) explore patient video monitoring and soft cell flooring<br><br>Outcome measure: improve falls with injury rates by 25%;
**Mikolaizak 2016 (51)**	Australia	Determine whether an approach involving timely assessment and tailored interventions, resulted in a meaningful reduction in subsequent falls, fall-related injuries and associated emergency health service use in older people who were	Older adults 65 years and over	RCT	MDT approach to care delivery in community (home) or primary care setting	Targeted identified risk factors and used existing services to implement physiotherapy, occupational therapy, geriatric assessment, optometry and medication management approaches to prevent falls	Community (home)	Micro	Outcome measure: Rate of falls and fall-related injuries during the 12-month follow-up period

For peer review only

		not transported to hospital following a fall							
**Peeters 2007 (45)**	Netherlands	Reduce the fall risk in older persons with a high risk of falling. The intervention consists of a systematic assessment of the putative causes of falling and subsequent targeted individualised preventive measures	Older adults >65	RCT	Care pathway	Assessment by geriatric outpatient clinic for falls risk factors in collaboration with a MDT that provides multifactorial treatment that may include physiotherapy, ophthalmic assessment and medicines management	Community (home)	Micro	Process measure: proportion assessed for; risk of recurrent falling, fall history, medical history, medication use, independence in activities of daily living<br>Outcome measure: Time to first and second fall
**Robbins 2013 (32)**	US	To describe the process followed and outcomes achieved from participating in a pharmacy clinical services and patient safety collaborative and integrating pharmacy services into a high-risk, ambulatory population	Adults	QI case study	Care pathway	Patient Safety and Clinical Pharmacy Services Collaborative change package involving a MDT	Health centre	Macro	Outcome measure: Percentage of adverse drug events detected in the population of focus that received clinical pharmacy services during the month of follow up
**Sze 2008 (50)**	Hong Kong	To investigate the efficacy of a falls prevention clinic and a community step-down programme in	Elderly patients	Prospective cohort	MDT approach to care delivery in community (home) or	12 week falls clinic provided by a MDT - including fall evaluation, balance training, home hazard	Community (home)	Micro	Primary outcome measure: Number of falls per person per year<br><br>Secondary outcome measure: Fall-related injuries

For peer review only

		reducing the number of falls among community-dwelling elderly at high risk of a fall			primary care setting	management program, and medical referrals. Followed by a community step-down (nine months) program, including falls prevention education, a weekly exercise class, and two home visitations			
**Toivo 2019 (46)**	Finland	To assess the impact of a care coordination intervention on medication risks identified in drug regimens of older home care clients over a one-year period	Elderly patients	Cluster RCT	MDT approach to care delivery in community (home) or primary care setting	Coordinated medication risk management (CoMM) model which involves a multi-stage approach; triage, medication risk screening and reviews, actions plans delivered by a MDT	Community (home)	Micro	Process measure: Several including use of harmful medications<br><br>Outcome: clinically significant drug interactions
**Van Melle 2018 (47)**	Netherlands	To pilot a review of medical records to identify transitional safety incidents (TSIs) for use in a large intervention study and assess its reliability and validity.	Adults under cardiology and gastro service care	Retrospective medical record review study	Integrated digital record	Transitional medical record system: establish inter-rater reliability of a measurement tool designed to assess transitional safety incidents	Hospital and primary care	Macro	Outcome measures: Transitional safety incidents which include but not restricted to adverse drug events leading to unplanned hospital admission
**Wallace 2018 (33)**	US	To evaluate the effects of a multidisciplinary hip fracture care pathway on the outcomes of the elderly patients	Elderly	Retrospective analysis	Care pathway	Multidisciplinary care pathway for reduce hip fractures in the elderly	Hospital (Trauma centre)	Micro	Outcome measures: Several – includes mechanism of injury (such as falls), hip fracture characteristics and hospital length of stay

For peer review only

**Willis 2011 (34)**	US	To determine if trained undergraduate students, in conjunction with pharmacists, could provide in home medication reviews, and demonstrate benefit to the health and welfare of a senior population affiliated Patient-Centred Medical Home	Patients aged 65 years and over	Service evaluation	MDT approach to care delivery in community (home) or primary care setting	Patient-Centred Medical Home model - In-home medication reviews, assessing a home for fall risk, and performing blood pressures	Community (home)	Meso	Process measures: Proportion of patients prescribed falls risk medication and medicines that have a high potential for major drug interaction. Proportion of patients that have had a medication change made to their clinical medication list

For peer review only

Preferred Reporting Items for Systematic reviews and Meta-Analyses extension for Scoping Reviews (PRISMA-ScR) Checklist

SECTION	ITEM	PRISMA-ScR CHECKLIST ITEM	REPORTED ON PAGE #
**TITLE**			
Title	1	Identify the report as a scoping review.	
**ABSTRACT**			
Structured summary	2	Provide a structured summary that includes (as applicable): background, objectives, eligibility criteria, sources of evidence, charting methods, results, and conclusions that relate to the review questions and objectives.	
**INTRODUCTION**			
Rationale	3	Describe the rationale for the review in the context of what is already known. Explain why the review questions/objectives lend themselves to a scoping review approach.	
Objectives	4	Provide an explicit statement of the questions and objectives being addressed with reference to their key elements (e.g., population or participants, concepts, and context) or other relevant key elements used to conceptualize the review questions and/or objectives.	
**METHODS**			
Protocol and registration	5	Indicate whether a review protocol exists; state if and where it can be accessed (e.g., a Web address); and if available, provide registration information, including the registration number.	
Eligibility criteria	6	Specify characteristics of the sources of evidence used as eligibility criteria (e.g., years considered, language, and publication status), and provide a rationale.	
Information sources*	7	Describe all information sources in the search (e.g., databases with dates of coverage and contact with authors to identify additional sources), as well as the date the most recent search was executed.	
Search	8	Present the full electronic search strategy for at least 1 database, including any limits used, such that it could be repeated.	
Selection of sources of evidence†	9	State the process for selecting sources of evidence (i.e., screening and eligibility) included in the scoping review.	
Data charting process‡	10	Describe the methods of charting data from the included sources of evidence (e.g., calibrated forms or forms that have been tested by the team before their use, and whether data charting was done independently or in duplicate) and any processes for obtaining and confirming data from investigators.	
Data items	11	List and define all variables for which data were sought and any assumptions and simplifications made.	
Critical appraisal of individual sources of evidence§	12	If done, provide a rationale for conducting a critical appraisal of included sources of evidence; describe the methods used and how this information was used in any data synthesis (if appropriate).	
Synthesis of results	13	Describe the methods of handling and summarizing the data that were charted.	

For peer review only

SECTION	ITEM	PRISMA-ScR CHECKLIST ITEM	REPORTED ON PAGE #
**RESULTS**			
Selection of sources of evidence	14	Give numbers of sources of evidence screened, assessed for eligibility, and included in the review, with reasons for exclusions at each stage, ideally using a flow diagram.	
Characteristics of sources of evidence	15	For each source of evidence, present characteristics for which data were charted and provide the citations.	
Critical appraisal within sources of evidence	16	If done, present data on critical appraisal of included sources of evidence (see item 12).	
Results of individual sources of evidence	17	For each included source of evidence, present the relevant data that were charted that relate to the review questions and objectives.	
Synthesis of results	18	Summarize and/or present the charting results as they relate to the review questions and objectives.	
**DISCUSSION**			
Summary of evidence	19	Summarize the main results (including an overview of concepts, themes, and types of evidence available), link to the review questions and objectives, and consider the relevance to key groups.	
Limitations	20	Discuss the limitations of the scoping review process.	
Conclusions	21	Provide a general interpretation of the results with respect to the review questions and objectives, as well as potential implications and/or next steps.	
**FUNDING**			
Funding	22	Describe sources of funding for the included sources of evidence, as well as sources of funding for the scoping review. Describe the role of the funders of the scoping review.	

JBI = Joanna Briggs Institute; PRISMA-ScR = Preferred Reporting Items for Systematic reviews and Meta-Analyses extension for Scoping Reviews.

* Where *sources of evidence* (see second footnote) are compiled from, such as bibliographic databases, social media platforms, and Web sites.

† A more inclusive/heterogeneous term used to account for the different types of evidence or data sources (e.g., quantitative and/or qualitative research, expert opinion, and policy documents) that may be eligible in a scoping review as opposed to only studies. This is not to be confused with *information sources* (see first footnote).

‡ The frameworks by Arksey and O'Malley (6) and Levac and colleagues (7) and the JBI guidance (4, 5) refer to the process of data extraction in a scoping review as data charting.

§ The process of systematically examining research evidence to assess its validity, results, and relevance before using it to inform a decision. This term is used for items 12 and 19 instead of "risk of bias" (which is more applicable to systematic reviews of interventions) to include and acknowledge the various sources of evidence that may be used in a scoping review (e.g., quantitative and/or qualitative research, expert opinion, and policy document).

From: Tricco AC, Lillie E, Zarin W, O'Brien KK, Colquhoun H, Levac D, et al. PRISMA Extension for Scoping Reviews (PRISMAScR): Checklist and Explanation. Ann Intern Med. 2018;169:467–473. doi: 10.7326/M18-0850.

For peer review only - http://bmjopen.bmj.com/site/about/guidelines.xhtml
